# Behavioral analyses of a forebrain glutamatergic neuron specific *Ywhae* conditional knockout mouse model

**DOI:** 10.1371/journal.pone.0335427

**Published:** 2025-11-11

**Authors:** Meaghan Navarrete-Mathews, Gloria S. Lee, Angel Walerio, Dylan Horne, Yuying Wu, Yi Zhou

**Affiliations:** Department of Biomedical Sciences, Florida State University College of Medicine, Tallahassee, Florida, United States of America; University of Nebraska Medical Center College of Medicine, UNITED STATES OF AMERICA

## Abstract

The seven mammalian isoforms of 14-3-3 are each encoded by a unique gene and function as phosphorylation dependent protein modulators. Because 14-3-3 proteins have particularly high expression in the brain, they have been implicated in a variety of neuronal functions. Recently, we showed that functional knockout of all 14-3-3 isoforms in forebrain glutamatergic neurons of mice is sufficient to induce schizophrenia-like endophenotypes. Human and animal studies have linked mutations in *Ywhae* and 14-3-3ε expression changes to certain neurodevelopmental and psychiatric diseases. In this study, we conditionally knocked out 14-3-3ε from forebrain glutamatergic neurons by crossing *Ywhae*^*flox/flox*^ mice with CaMKIIα-Cre mice. *Ywhae*^*flox/flox*^ *Cre*^*+*^ (conditional knockout -CKO) mice and their *Ywhae*^*flox/flox*^ *Cre*^*-*^ (double-flox control - dFlC) littermates were put through a battery of behavioral tests to assess their behavioral endophenotypes. *Ywhae* CKO mice exhibited significant differences from dFlC mice in some of the behaviors examined. We also found several significant sex differences within our model. Furthermore, we compared two viral 14-3-3 knockout methods and found that CaMKIIα promoter driven difopein expression in wildtype mice is more efficient than Cre/loxP driven difopein expression in CaMKIIα-Cre mice. Collectively our results indicate that knocking out 14-3-3ε in glutamatergic forebrain neurons via this strategy is not sufficient to induce schizophrenia-like behavioral alterations. In the future, using different mouse line or knockout scheme may help further elucidate the isoform specific role of 14-3-3ε in the forebrain.

## Introduction

The 14-3-3 protein family is highly conserved and ubiquitous among eukaryotes [1]. In mammals, there are seven distinct 14-3-3 protein isoforms, each of which is encoded by a different gene: *YWHAB* (β), *YWHAG* (γ), *YWHAE* (ε), *YWHAZ* (ζ), *YWHAH* (η), *YWHAQ* (θ), and *YWHAS* (σ) [2]. The crystal structure of 14-3-3 proteins reveals that two highly helical L shaped 14-3-3 protein monomers form a cup-shaped hetero or homodimer [3,4]. The concave face of the 14-3-3 dimer is composed of mostly conserved amino acids that form two amphipathic ligand binding grooves which interact with phosphoserine and phosphothreonine containing motifs, such as RSXpSXP and RXY/FXpSXP [5,6]. Evidence also suggests that contacts with the variable convex region of the 14-3-3 dimer can influence binding affinity in an isoform-dependent manner. The unique structure of the 14-3-3 dimer thus allows a diverse range of hundreds of different ligands to bind in a phosphorylation-dependent manner, resulting in the modulation of the subcellular location or function of its binding partners. By doing so, the 14-3-3 proteins play an important modulatory role in many different cellular functions like intracellular signaling, apoptosis, ion channel function, and cell surface expression [7,8].

The functions of 14-3-3 proteins in the nervous system have been of particular interest to researchers, as they make up approximately 1% of the total soluble proteins in the brain [9]. Accumulating evidence demonstrates that 14-3-3 proteins play important roles in neuronal functions like neural differentiation and migration, receptor trafficking, and neurotransmitter release [7]. Mouse models have been used to study behavioral, synaptic, molecular, and anatomical changes associated with 14-3-3 protein knockout (S1 Table). In addition, human studies have identified genetic alterations in the 14-3-3 genes and changes in 14-3-3 mRNA or protein levels in neurological disorders such as schizophrenia [10,11].

We recently generated a transgenic functional knockout (FKO) mouse model, in which all seven 14-3-3 isoforms are functionally inhibited in the forebrain by a dimeric 14-3-3 peptide inhibitor (difopein), expressed under the neuron-specific Thy-1 promoter. This results in behavioral, molecular, and electrophysiological phenotypes reminiscent of those observed in human schizophrenia patients [10,12–14]. The 14-3-3 family is known to play a role in the normal function of glutamatergic signaling; including modulating pre-synaptic ion-channels, post-synaptic glutamatergic receptors, and synaptogenesis [15]. Therefore, we created a region and cell type specific model in which the 14-3-3 proteins are functionally inhibited in the glutamatergic neurons of the dorsal CA1 region of the hippocampus (dCA1) via a difopein-expressing virus driven by the calcium/calmodulin-dependent protein kinase II alpha (CaMKIIα) promoter [16,17]. Interestingly, we found that functional inhibition of the 14-3-3 proteins within this specific population causes reduced dendritic complexity and increased intrinsic excitability of these neurons [18]. Dysfunction of the glutamatergic system and hippocampal abnormalities have long been associated with schizophrenia [19,20]. In particular, an imbalance in the excitatory to inhibitory (E/I) signaling ratio in the forebrain is thought to underlie the pathophysiology of schizophrenia [21]. However, research in this area has largely focused on the role of decreased inhibitory signaling from GABAergic interneurons and altered N-methyl-D-aspartate (NMDA) receptor functions [22–24]. The findings from our transgenic and viral 14-3-3 FKO models are consistent with the E/I imbalance hypothesis and highlight the understudied role of glutamatergic neurons in the maintenance and dysfunction of the E/I balance. Further, our results suggest that the manipulation of 14-3-3 proteins in the forebrain can help us understand the neurobiological mechanisms underlying the development of schizophrenia.

While our previous results indicate the importance of 14-3-3 proteins in CaMKIIα positive glutamatergic neurons of the forebrain, it remains unknown whether the loss of certain 14-3-3 isoforms is critical to this phenotype. Thus, we aim to investigate the role of specific 14-3-3 isoforms in CaMKIIα positive forebrain neurons.

Among the studies that implicate the 14-3-3 family in schizophrenia, there is strong evidence linking 14-3-3ε specifically to the disorder [11]. This includes the identification of common variants in the *YWHAE* gene that confer risk of schizophrenia and changes in *YWHAE* mRNA and 14-3-3ε protein expression levels [25–31]. *YWHAE* variations can also contribute to neurodevelopmental and psychiatric conditions that are co-morbid or share underlying pathophysiology with schizophrenia; such as attention-deficit/hyperactivity disorder (ADHD) [32], major depressive disorder (MDD) [33], bipolar disorder (BD) [34], suicidal behavior [35], and Alzheimer’s disease [36]. Moreover, microduplications or microdeletions in 17p13.3, the chromosomal location of YWHAE, can cause learning disabilities, autism spectrum disorder (ASD), epilepsy, lissencephaly, and Miller-Dieker syndrome [37–39].

Further, animal models have linked the loss of 14-3-3ε to pathophysiological phenotypes that correlate to symptoms of human psychiatric disorders. A group of studies investigated the anatomical, developmental, and behavioral alterations of whole brain, embryonic *Ywhae* knockout mice [25,40–42]. One study included male and female subjects but did not perform sex specific analyses [41], while the others only included males [25,40,42,43]. Both heterozygous and homozygous *Ywhae* knockouts show neurodevelopmental abnormalities in the cortex and hippocampus. This included a less cellular and disorganized pyramidal layer, shorter and slower neuronal migration, and abnormal tyrosine hydroxylase (TH) positive dendritic spines in the orbital cortex. [40,42]. Unsurprisingly, homozygous knockout mice exhibit a high degree of embryonic mortality. Nevertheless, this evidence mirrors pathology seen in human schizophrenia and suggests a significant role for 14-3-3ε in neuronal development. There are also several behavioral abnormalities observed post-developmentally in this model. Heterozygous *Ywhae* knockout mice from a 129/S6 x NIH Black Swiss background show a weak working memory deficit in the eight-arm radial maze and moderately enhanced anxiety-like behavior in the elevated plus-maze [25]. While heterozygous and homozygous *Ywhae* knockouts from a 129/SVE x C57BL/6J background show locomotor hyperactivity, increased sociability, as well as visual and spatial memory deficits [43].

Our previous results, demonstrating that the loss of 14-3-3 proteins in glutamatergic CA1 neurons leads to psychosis-related phenotypes, and the link between YWAHE/14-3-3ε and psychiatric disorders inspired our generation of a *Ywhae* conditional knockout (CKO) mouse model to better understand the role of 14-3-3ε in glutamatergic forebrain neurons. Within this model, region and cell type specific Cre-driven recombination of the *Ywhae* gene is directed by the CaMKIIα promoter, which is highly expressed in glutamatergic neurons of the forebrain. Because the CaMKIIα promoter is activated postnatally [44–47], this scheme allows us not only to circumvent the embryonic lethality of previous *Ywhae* knockout models but also to study the isoform specific role of 14-3-3 within a specific population of neurons. In this study, we characterized the locomotor, memory, social, and sensorimotor gating behaviors of homozygous *Ywhae* CKO mice compared to their littermate controls. Both male and female mice were included, and sex-specific analyses were performed to assess whether sex contributed as a biological variable to our results.

## Materials and methods

### Animal use

All animal procedures were carried out in accordance with the guidelines for the Care and Use of Laboratory Animals of the Florida State University (FSU) and approved by the FSU Animal Care and Use Committee (Protocol number 202200000018). Prior to handling animals, research staff completed online training in common compliance issues, humane endpoints, pain management in laboratory animals, and post-procedure care of mice and rats in research: minimizing pain and distress. The humane endpoints for this study included animals that showed persistent signs of distress (e.g., 20% or greater loss of body weight, dehydration, skin chewing, discharge around the eyes, loss of appetite, ptosis, or lack of activity) or/and continuous infection. If humane endpoints were reached, the animal would be immediately euthanized using CO2. A total of 112 mice underwent testing for the *Ywhae* behavioral battery which lasted 4–5 weeks. Animals were group housed and behavioral testing was done from least to most stressful to minimize distress. During the testing period, animal wellbeing was monitored daily and there were zero mice that met humane endpoint criteria and zero mice that were found dead. Immediately following the completion of the last behavioral test, all 112 mice were euthanized by either rapid decapitation or by perfusion with 4% paraformaldehyde (animals first anesthetized with an intraperitoneal injection of 100 mg/kg Ketamine + 10 mg/kg Xylazine). A total of 12 mice underwent stereotaxic surgery for viral injection experiments, which lasted 2–3 weeks. Mice were group housed until they underwent stereotaxic surgery, after which they were single housed to allow for recovery from surgery. An intraperitoneal injection of 100 mg/kg Ketamine + 10 mg/kg Xylazine was used for anesthesia prior to surgery and subcutaneous injections of 20 mg/kg Ketoprofen were used for analgesia immediately following surgery and 24 hours later. After surgery, animals were monitored daily for proper recovery for two weeks. There were zero mice that met humane endpoint criteria and zero mice that were found dead. Following the final open field test session, all 12 mice were euthanized by perfusion with 4% paraformaldehyde (animals first anesthetized with an intraperitoneal injection of 100 mg/kg Ketamine + 10 mg/kg Xylazine).

### Generation of 14-3-3ε conditional knockout mice

**Fig 1:**
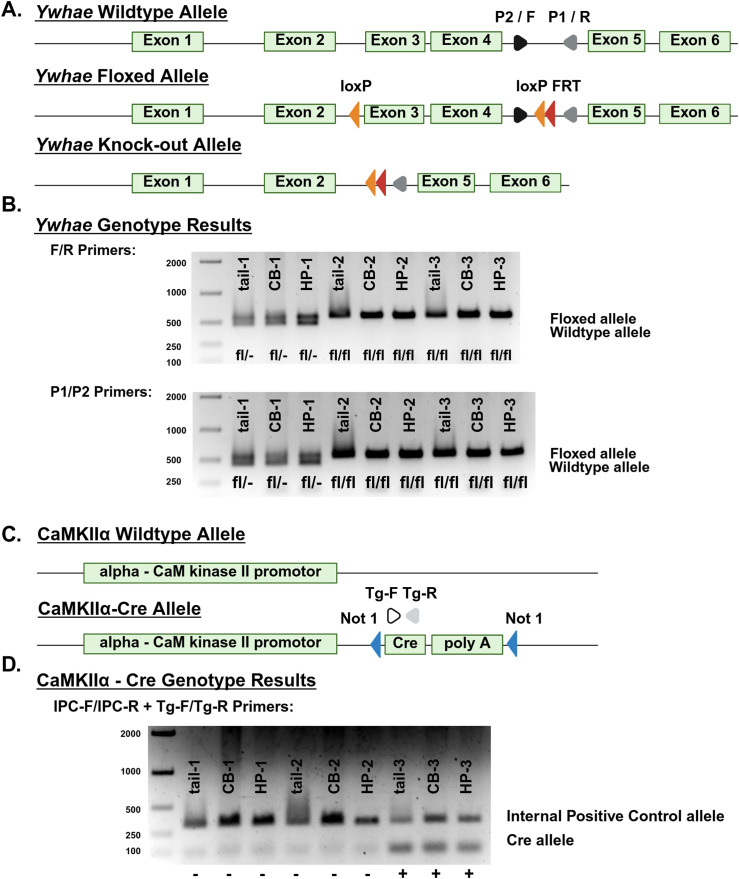
*Ywhae* knockout strategy and genotype validation. *Ywhae*^*flox/flox*^ mice have loxP sites flanking exons 3 and 4 of the *Ywhae* allele. Cre/loxP recombination in *Ywhae*^*flox/flox*^ *Cre*^*+*^ mice results in the excision of exons 3 and 4. The P1/P2 and F/R primer sets recognize an intronic region between exons 4 and 5, allowing for the differentiation of wildtype and floxed *Ywhae* alleles **(A)**. The F/R and P1/P2 primer sets are used to distinguish wildtype (-/-) mice from those that are homozygous (fl/fl) or heterozygous (fl/-) for the *Ywhae* floxed allele in tail, cerebellum (CB), and hippocampus (HP) tissue **(B)**. *CaMKII*α-*Cre* mice have Cre recombinase inserted downstream of the calcium/calmodulin-dependent protein kinase II alpha promoter. The Tg-F/Tg-R primer set recognize a portion of the Cre recombinase transcript **(C)**. The internal positive control (IPC)-F/IPC-R and Tg-F/Tg-R primer sets are used to distinguish mice with the CaMKIIα wildtype (-) and CaMKIIα-Cre (+) allele in tail, cerebellum (CB), and hippocampus (HP) tissue **(D)**.

Transgenic 14-3-3ε conditional knockout (CKO) mice were generated by first backcrossing *Ywhae*^*flox/flox*^ mice (RRID:IMSR_JAX:029055) [48], which possess loxP sites flanking exons 3−4 of the *Ywhae* gene, to wildtype C57BL/6J mice (RRID:IMSR_JAX:000664) for at least eight generations. In order to conditionally knockout the *Ywhae* gene in CaMKIIα expressing neurons in the forebrain, *Ywhae*^*flox/flox*^ mice were then bred with transgenic T29-1 mice from a C57BL/6J background (RRID:IMSR_JAX:005359) [49], in which the CaMKIIα promoter drives expression of Cre recombinase primarily in the pyramidal neuron layer of the hippocampal CA1. Generation of *Ywhae*^*flox/flox*^ *Cre*^*+*^ Conditional Knockout (CKO) mice and their *Ywhae*^*flox/flox*^ *Cre*^*-*^ double-flox control (dFlC) littermates was optimized with a *Ywhae*^*flox/-*^ *Cre*^*+*^ X *Ywhae*^*flox/flox*^ *Cre*^*-*^ mating scheme. Transgenic CKO and dFlC mice were identified by polymerase chain reaction (PCR) genotyping using the following primer sets (Fig 1):

(1) *Ywhae* Forward (F) (CACATACCCACCCACAGTGC)/ *Ywhae* Reverse (R) (CAGTAAGCCATCTCCCTAGTCA),
(2) P1 (AGGTACCAAAACAGTAAGCCATCTCCCTA)/ P2 (GCATGTGTTTGTCTGTCAGAGGAC),
(3) Transgene Forward (Tg-F) (GCGGTCTGGCAGTAAAAACTATC)/ Transgene Reverse (Tg-R) (GTGAAACAGCATTGCTGTCACTT)/ Internal Positive Control Forward (IPC-F) (CTAGGCCACAGAATTGAAAGATCT)/ Internal Positive Control Reverse (IPC-R) (GTAGGTGGAAATTCTAGCATCATCC).

### Body weight analysis

The body weights of a cohort of CKO and dFlC mice that were not used for behavioral experiments were recorded once a week for 8 weeks to track developmental growth (CKO: N = 18, dFlC: N = 12). In addition, two cohorts of mice undergoing 4–5 weeks of behavioral experiments had their body weights recorded after the first and last behavioral tests (CKO-male: N = 12, CKO-female: N = 12, dFlC-male: N = 14, dFlC-female: N = 13).

### Behavioral assays

Animals were group housed and kept on a 12-hour light-dark cycle. Four cohorts of adult male and female CKO mice and their dFlC littermates (total CKO-male: N = 27, CKO-female: N = 21, dFlC-male: N = 21, dFlC-female: N = 26) (age at first behavioral test between 8–17 weeks) underwent a battery of behavioral assays as previously described [10,12,16]. Variation in sample numbers across the different behavioral tests reflects the exclusion of animals because of equipment malfunction as well as certain animals or cohorts not successfully completing all assays.

**Fig 2:**
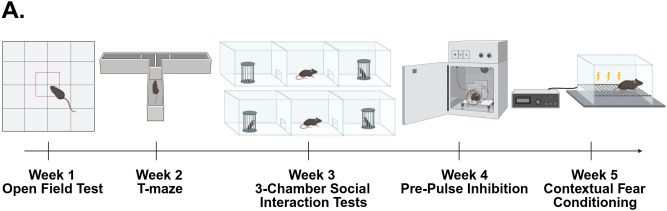
Timeline of behavioral testing. One behavioral test is performed a week over a period of 5 weeks.

Testing was done over a period of 4–5 weeks, during which one behavioral assay was performed during the light cycle each week. Assays were performed in order of least to most stressful for the animal: (week 1) open field test (OFT), (week 2) T-maze, (week 3) 3-chamber social interaction tests, (week 4) pre-pulse inhibition (PPI), (week 5) contextual fear conditioning (Fig 2). Prior to each test, mice were acclimated to the testing room for at least 1 hour. Procedures for each behavioral assay are briefly described below.

For open field testing, mice were placed into the center of a square open field arena (Med Associates Open Field Arena, 43.2 cm × 43.2 cm × 30.5 cm, with IR photo-beam sensors) and allowed to locomote freely for 30 minutes. The total distance traveled and the total number of rearing events in the 30-minute time-period was measured using Med Associates Activity Monitor software.

For the T-maze test, an opaque testing apparatus consisting of three removable arms arranged in the shape of a ‘T’ was used. Three distinct visual cues were placed around the test area, one visible from each arm of the test apparatus. Mice were placed in the middle of the test apparatus with all three arms blocked off for a habituation period of 5-minutes, after which the arms were unblocked, and the animal was allowed to freely explore the maze for 10-minutes while their activity was recorded. Experimenters were blinded to genotype and sex when analyzing data. The order of arm choices was analyzed to determine the total number of complete alternations (three consecutive arm choices) and the total number of correct alternations ((all three arms entered consecutively (i.e., C-B-A). The percentage of correct alternations was calculated as:

$\left[\left(\frac{Total\;\#\;of\;Correct\;Alternations}{Total\;\#\;of\;Alternations}\right)\times 100\right]$For the 3-chamber social interaction tests (sociability and social novelty), the test apparatus consisted of a Plexiglas testing arena (24” x 24” x 12”) divided equally into three chambers by two walls with 3” diameter cut-outs and two wire cups, under which an unfamiliar or familiar conspecific mouse was held. For sociability, the test mouse was habituated to the middle chamber for 5-minutes, after which the test mouse was allowed to freely explore all chambers for 10-minutes. The number of entries into and the time spent in each chamber (empty, middle, containing an unfamiliar mouse) was recorded. For social novelty, the previously empty chamber contained a new unfamiliar mouse. The test mouse was subsequently re-habituated to the middle chamber for 5-minutes, and then allowed to freely explore all three chambers for 10-minutes. Experimenters were blinded to genotype and sex when analyzing data. The number of entries into and the time spent in each chamber (middle, containing either unfamiliar or familiar mouse) was recorded. The chamber (left or right) that contained the empty cup, familiar conspecific, and unfamiliar conspecific were counterbalanced across trials.

For the pre-pulse inhibition test, acoustic startle response and pre-pulse inhibition (PPI) of the acoustic startle response were assessed using Acoustic Startle Reflex package (Med Associates). The test mouse was placed in a clear cylindrical Plexiglas holder within a sound attenuating cubicle, and their responses to acoustic stimuli were recorded with 60 dB background noise. Baseline startle amplitude responses were first measured during a block of ten trials consisting only of startle tone (90 dB). PPI was determined in the subsequent block which consisted of pseudo-randomized delivery of eight startle tone only trials, eight no stimuli trials, eight pulse tone only trials at four different levels (67, 70, 73, or 76 dB), and eight pulse paired with startle tone trials. PPI percentage was calculated as:

$\left\{\left[1-\left(\frac{Mean\;of\;Startle\;with\;Prepulse}{Mean\;of\;Startle\;without\;Prepulse}\right)\right]\times 100\right\}$Due to lower sample numbers, PPI was analyzed for only genotype differences and not sex differences.

The contextual fear conditioning test was performed over a period of two consecutive days using the Contextual NIR Video Fear Conditioning System for Mouse and Video Freeze Software (Med Associates). For training on day one, mice were placed in the testing chamber and their freezing behavior was recorded before (3 minutes), during, and after (2 minutes) three foot-shocks (2 sec, 0.50 mA, separated by a 2-minute inter-shock interval). For testing on day two, mice were placed back into the same chamber and their freezing behavior was recorded for 3 minutes. Percentage of time spent freezing post-fear stimulus on day 1 (post-FS) and during the test session on day 2 (Test) were calculated by the Video Freeze Software (Med Associates).

### Immunohistochemistry

Mice were anesthetized and transcardially perfused with 4% paraformaldehyde in 0.1 M phosphate buffer, pH 7.4 (PBS). After an overnight post-fixation in the same fixative at 4°C, the brains were then cut into 40μm sections on a Vibratome (Leica Microsystems).

*Ywhae* brain sections were immunostained with the polyclonal antibodies against CaMKIIα (1:50) (Abcam Cat# ab22609, Lot No. GR30131−1, RRID:AB_447192) and either 14-3-3ε (1:100) [50] or 14-3-3ζ (1:100) [50] for 48 hours at 4 degrees Celsius, followed by incubation with donkey-anti-mouse Alexa Fluor Plus 594 (1:500) (Thermo Fisher Scientific Cat# A21203, Lot No. 2069656, RRID:AB_2535789) and donkey-anti-rabbit FITC (1:500) (SouthernBiotech Cat# 4050−02, Lot No. G2919-N070E, RRID:AB_2795952) secondary antibodies for 24 hours at room temperature. Brain sections were then incubated with 300 nM DAPI (Invitrogen, P3571) for 10 min before being mounted using an antifade mounting medium (VECTASHIELD). Representative fluorescence images (resolution 512x512 pixels) were acquired using a Andor Revolution Spinning Disk Laser Confocal Microscope (Oxford Instruments) using a 20 × objective performed with identical gain, contrast, exposure, and laser excitation.

Brain sections from viral experiments were incubated with 300 nM DAPI (Invitrogen, P3571) for 10 min before being mounted using an antifade mounting medium (VECTASHIELD). Representative fluorescence images (resolution 121x121 pixels) were acquired using a Zeiss LSM 880 Meta confocal microscope (Zeiss, Germany) using a 20 × objective performed with identical gain, contrast, exposure, and laser excitation.

### Viral vectors

AAV2/9-CaMKIIα-YFP-difopein (titer at 7.82E12 v.g./ml) was constructed and produced by OBiO Technology (Shanghai) Corp., Ltd. Briefly, cDNA encoding YFP-difopein was subcloned into a CaMKIIα-MCS rAAV plasmid. AAV9-CAG-DIO-YFP-difopein (titer at 5E13 g.c/ml) was constructed at Florida State University and produced by Syd Labs, Inc. (Boston, MA). Briefly, cDNA encoding YFP-difopein, was subcloned into a CMV-DIO-EGFP rAAV plasmid, replacing EGFP. The viruses were then produced using the triple transfection method in HEK 293 cells and AAV titers were determined by real-time PCR. Upon arrival, all viral vectors were aliquoted and stored at −80°C prior to stereotaxic injections.

### Stereotaxic surgery for viral injections

Wildtype and transgenic T29-1 mice from a C57BL/6J background were used for viral injection experiments (N = 6 each group). Mice were housed on a 12-hour light/dark cycle and provided access to water and food ad libitum. Mice were housed in groups of 2–4 per cage until they underwent stereotaxic surgery. Following the surgical procedure, mice were single caged until all experimental procedures were finished. Mice were handled daily for 2 weeks prior to behavioral tests, which were conducted during the light cycle. One day after undergoing a baseline OFT session, mice (age 8–16 weeks) (male and female) were anesthetized with intraperitoneal (i.p.) injections of a mixture of ketamine (100 mg/kg)/xylazine (10 mg/kg) and placed in a stereotaxic frame (David Kopf Instruments, Tujunga, CA). The animal’s skull was exposed via a small incision on the scalp. Small burr holes were made directly above the viral injection sites bilaterally using a micro-precision drill. For micro-injections, Hamilton syringes (10 μl, 33-gauge) loaded with AAV virus were slowly lowered into the target area according to the corresponding coordinates: dorsal CA1 (dCA1) (AP: − 2.0 mm, ML: ± 1 mm, DV: −1.1 mm). Virus (0.5–1 ul) was slowly injected at 75 nl/minute. Injection needles were left in place for an additional 10–15 minutes to assure adequate viral delivery before slowly being withdrawn. The scalp incision was then closed and treated with topical neomycin. For postoperative care, ketoprofen (5 mg/kg in 0.05 ml sterilized saline) was used for pain relief immediately following surgery. Mice were given 2 weeks for expression of viral proteins and recovery from surgery before undergoing a second OFT session.

### ARRIVE guidelines (Animal Research: Reporting of In Vivo Experiments)

We chose to use dFlC mice as the control group, rather than wildtype C57BL/6J mice, as they are produced through the same mating scheme as the experimental CKO mice and are only different in that they do not express Cre recombinase. Based on an a-priori calculation using the G*Power3.1.9.7 software, we determined that a total sample size of 128, or 32 animals per group, would be needed to detect a medium effect size of 0.25 with an α = 0.05, power = 0.8, numerator df = 1, and number of groups = 4. Actual sample sizes per group varied after accounting for exclusion of certain animals due to inability to complete behavioral assays or equipment malfunction during testing. Subsequently, the power of each outcome measure varied with the sample sizes. In this study group sizes range from 6−27, which is comparable to the group sizes of 5−15 used in our previously published 14-3-3 functional knockout (FKO) studies, where significant effects were detected in the same behavioral tests used here. Mice were sorted into groups based on their sex and genotype. When analyses were computed by hand, experimenters were blinded to both the sex and genotype of each animal. Outcome measures were determined a-priori based on previous *Ywhae* knockout studies done by us and others. Statistical tests were determined for each experiment a-priori depending on how many variables were compared.

### Statistical analysis

Adolescent weight gain, PPI, and viral injection experiments were analyzed for genotype differences only. All other outcome measures were analyzed for both genotype and sex differences. Between-subjects design was used for each experiment. All data were assessed by mixed-effects model (REML), one-way analysis of variance (ANOVA), two-way ANOVA, or three-way ANOVA using GraphPad Prism version 10.2.1 for Windows (GraphPad Software, Boston, Massachusetts USA, www.graphpad.com). Where there was a significant interaction effect, the relevant means with corresponding 95% confidence intervals are graphed in an interaction plot. A value of *p < 0.05 was considered a statistically significant difference. A post-hoc sensitivity analysis, computed with G*Power 3.1.9.7, indicated that across our behavioral assays we had the ability to detect a small to medium effect size between 0.318–0.400 with a power of 0.8.

## Results

### Generation and validation of *Ywhae* CKO mice

To assess the functional role of the 14-3-3ε protein in the glutamatergic neurons of the forebrain, we constructed a transgenic line of *Ywhae* conditional knockout (CKO) mice. This was achieved by crossing *Ywhae*^*flox/flox*^ mice, which have loxP sites flanking the exons 3 and 4 of the *Ywhae* gene (Fig 1A), to transgenic T29-1 mice, in which the CaMKIIα promoter drives expression of Cre recombinase in the pyramidal layer of the hippocampal CA1 (Fig 1C). The resulting CKO mice exhibited conditional knockout of exons 3 and 4 of the *Ywhae* gene in CaMKIIα positive neurons of the forebrain. Littermates which were negative for Cre were used as double-flox controls (dFlC). Genotypes of all experimental mice were determined by PCR at weaning using tail tissue and validated postmortem using cerebellum (CB) and hippocampus (HP) tissue with three sets of primers (Fig 1B, 1D).

**Fig 3:**
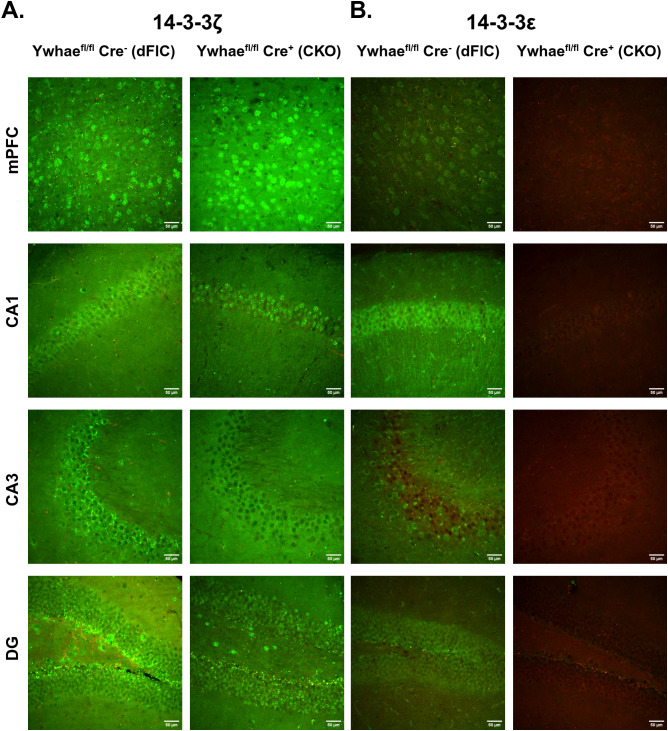
Validation of isoform-specific conditional knockout. Brain slices from dFlC and CKO mice were immunostained with an antibody against CaMKIIα (red) and either 14-3-3ζ (green) (A) or 14-3-3ε (green) **(B)**. Representative 20x confocal images of the medial prefrontal cortex (mPFC) and the hippocampal CA1, CA3, and dentate gyrus (DG) subfields are shown, scale bar = 50 µm.

To visualize the isoform specific knockout of 14-3-3 proteins within the forebrain of CKO animals, we performed immunohistochemistry using antibodies against CaMKIIα and either 14-3-3ε or 14-3-3ζ (S1 Fig). In forebrain areas containing CaMKIIα-positive neurons [the medial prefrontal cortex (mPFC), the CA1, CA2, and CA3 hippocampal subfields, and the dentate gyrus (DG)], both CKO and dFlC mice showed similarly robust 14-3-3ζ staining (Fig 3A). In these same regions, CKO mice showed a significant reduction in 14-3-3ε expression when compared to dFlC mice (Fig 3B) (S2 Fig). Suggesting that 14-3-3ε is in fact conditionally knocked out in the forebrain of CKO mice.

**Fig 4:**
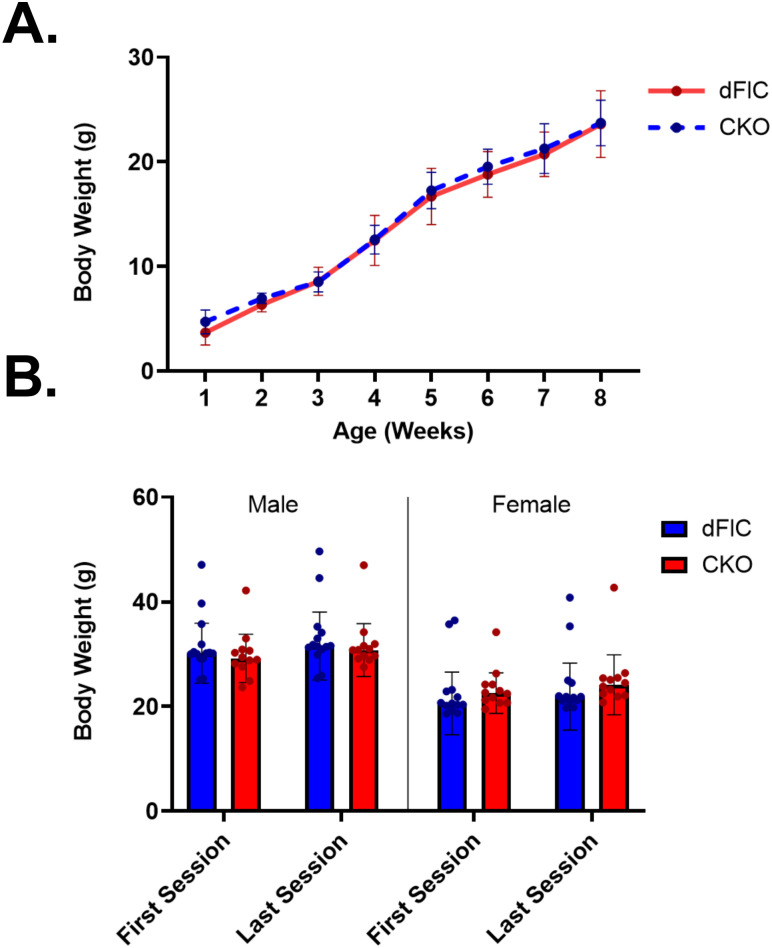
Body weight assessment. CKO mice show normal development and maintain healthy body weight throughout behavioral testing. CKO mice (N = 18) gain weight postnatally at the same developmental trajectory as dFlC mice (N = 12) from 1 week to 8 weeks of age **(A)**. Neither male nor female CKO mice differ from male or female dFlC mice in weight at first behavioral testing session nor weight at last behavioral testing session (CKO-male: N = 12, CKO-female: N = 12, dFlC-male: N = 14, dFlC-female: N = 13) **(B)**.

Whole brain embryonic knockout of *Ywhae* has been shown to be lethal in several studies, with homozygous knockout mice dying before or shortly after birth [40]. The CaMKIIα promoter is expressed mainly in the glutamatergic neurons of the forebrain starting at postnatal day 1 (P1) [51,52]. Using this promoter to drive conditional knockout of 14-3-3ε allowed us to circumvent the embryonic lethality seen in previous models. Based on the mating scheme used here, 25% of each litter is expected to be *Ywhae* CKO mice. In a sampling of 48 litters, the average percentage of CKO mice in each litter was 21.53% (95% CI [15.61%, 27.45%]). Thus, *Ywhae* CKO mice showed normal survival rates. Further, they did not differ in appearance at birth nor differ from dFlC littermates in postnatal developmental trajectory (REML; Week: F(3.007, 42.96)=316.4, p < 0.0001; Genotype; F(1, 28)=4.114, p = 0.0521; Interaction: F(7, 100)=0.8077, p = 0.5829) (Fig 4A), and maintained healthy body weight during behavioral testing (Three-way ANOVA; Genotype: F(1, 47)=0.08935, p = 0.7663; Session: F(1, 47)=54.51, p < 0.0001****; Sex: F(1, 47)=24.57, p < 0.0001****; Session X Sex: F(1, 47)=0.1178, p = 0.7329; Session X Genotype: F(1, 47)=1.210, p = 0.2769; Sex X Genotype; F(1, 47)=0.5873, p = 0.4473; Session X Sex X Genotype: F(1, 47)=0.8296, p = 0.8296) (Fig 4B).

### Evaluation of locomotor activity and anxiety behavior

Several different 14-3-3 knockout models display dysfunction in locomotor activity and anxiety behavior. Functional knockout (FKO) of all 14-3-3 isoforms in the forebrain of mice results in novelty-induced hyperactive locomotor activity in a 30-minute open field test (OFT) [10,16]. Heterozygous *Ywhae* knockout mice show increased vertical activity in the OFT, increased number of entries and increased time in closed arms in the elevated plus-maze [25]. While homozygous *Ywhae* knockout mice show increased locomotor activity, increased vertical activity, and decreased time spent in the center in a 10-minute OFT [43]. Thus, we sought to investigate whether *Ywhae* knockout in glutamatergic forebrain neurons would have similar effects on locomotor behavior and anxiety behavior.

**Fig 5:**
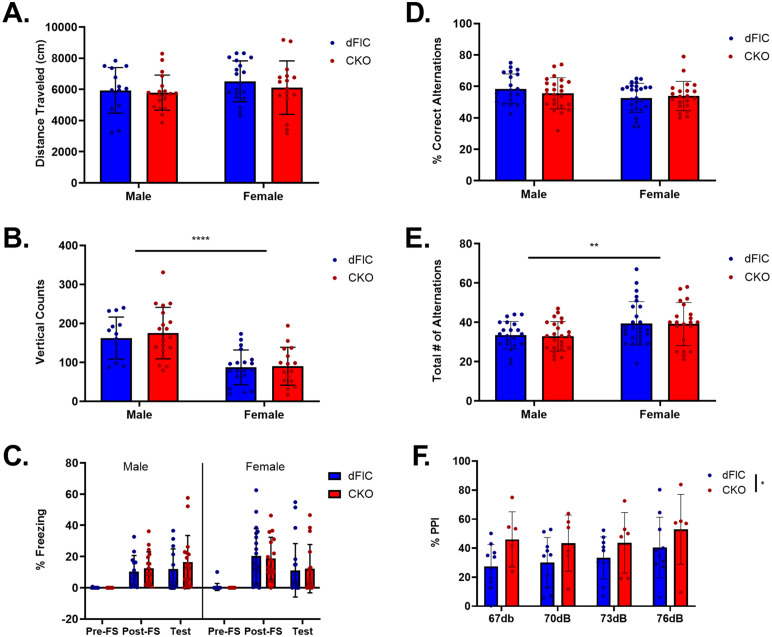
Evaluation of locomotor activity, memory performance, and sensorimotor gating ability. Behaviors were assessed for both genotype and sex differences. There was no significant sex or genotype effect in distance traveled (CKO-male: N = 19, CKO-female: N = 16, dFlC-male: N = 14, dFlC-female: N = 19) **(A)**. There was a significant main effect of sex in vertical rearing events, with males exhibiting a higher number of vertical counts than females (CKO-male: N = 19, CKO-female: N = 16, dFlC-male: N = 14, dFlC-female: N = 19) **(B)**. There is a significant main effect of test session and no main effect of genotype or sex in % freezing in the contextual fear conditioning test (CKO-male: N = 17, CKO-female: N = 16, dFlC-male: N = 13, dFlC-female: N = 20) **(C)**. No sex or genotype effects are observed in the % correct alternations in the T-maze (CKO-male: N = 24, CKO-female: N = 21, dFlC-male: N = 21, dFlC-female: N = 26) **(D)**. There is no genotype difference observed in the total # of alternation in the T-maze, but the main effect of sex suggests that male mice show a significantly lower total # of alternations compared to female mice (CKO-male: N = 24, CKO-female: N = 21, dFlC-male: N = 21, dFlC-female: N = 26) **(E)**. CKO mice (N = 6) show a trend of increased %PPI compared to dFlC mice (N = 10) with no main effect of auditory pre-pulse stimuli level in the PPI test **(F)**.

During a 30-minute OFT session, the distance traveled, and the total number of rearing events (vertical counts) were recorded to assess locomotor activity. There was no statistically significant effect of genotype or sex in distance traveled (Two-way ANOVA; Genotype: F(1, 64)=0.6512, p = 0.4227; Sex: F(1, 64)=1.751, p = 0.1905; Interaction: F(1, 64)=0.1414, p = 0.7082) (Fig 5A). Because other 14-3-3ε knockout models used a 10-minute OFT to assess locomotor activity, it is possible that a genotype difference may be obscured by acclimation during a 30-minute OFT. To assess this possibility, distance traveled was broken down into three 10-minute time-bins and compared. While animals showed a significant difference in distance traveled across the three time-bins, indicating that they did acclimate to the apparatus over the course of 30-minutes, there still was neither a genotype nor a sex difference (S3 Fig).

Rearing activity in the OFT can be interpreted as an indicator of anxiety levels. While there was no genotype effect in vertical counts measured, there was a main effect of sex, with males exhibiting a higher number of vertical counts than females (Two-way ANOVA; Genotype: F(1, 64)=0.3546, p = 0.5536; Sex: F(1, 64)=36.62, p < 0.0001****; Interaction: F(1, 64)=0.1436, p = 0.7059), a sex difference that has been noted in mice from a C57Bl/6J background [53](Fig 5B). In addition, there was a significant effect of age, with mice 3–4 months of age performing more vertical counts than mice 2–3 months of age (S4 Fig), which is concordant with other studies [54]. To further assess whether *Ywhae* CKO mice may display altered anxiety behavior in the OFT, we measured thigmotaxis. There was no genotype difference observed in the ratio of time spent at the edge versus the center of the OFT arena. However, there was a sex difference, indicating that female mice spent more time at the edge of the arena (S5 Fig).

### Assessment of memory performance

Memory impairments are seen in several different 14-3-3 knockout models. 14-3-3 FKO mice display decreased alternation percentage in the T-maze test, as well as decreased freezing behavior in the contextual fear conditioning test [10,12]. Heterozygous *Ywhae* knockout mice show increased number of revisiting errors in the 8-arm radial maze [25], while homozygous *Ywhae* knockout mice display decreased novel object recognition [43]. Therefore, we investigated whether *Ywhae* knockout in glutamatergic forebrain neurons would have similar effects on memory performance.

In the contextual fear conditioning test, animals learn to associate a neutral location (test chamber) with an aversive stimulus (foot shock). The percentage of time spent exhibiting freezing behavior before (pre-FS) and after the fear stimulus (post-FS), as well as during a test session the following day (Test) are recorded. The percent freezing during the test session reflects associative learning and memory abilities [48,55]. Here, we observed a statistically significant main effect of test session in the percentage of time spent freezing (Three-way ANOVA; Genotype: F(1, 62)=0.1816, p = 0.6715; Sex: F(1, 62)=0.7376, p = 0.3937; Test Session: F(1.912, 118.6)=43.76, Geisser-Greenhouse’s epsilon = 0.9562, p < 0.0001****; Test Session x Genotype: F(2, 124)=0.4210, p = 0.6573; Test Session x Sex: F(2, 124)=4.9, p = 0.0090**; Genotype x Sex: F(1, 62)=0.2996, p = 0.5861; Test Session x Genotype x Sex: F(2, 124)=0.1226, p = 0.8847) (Fig 5C). The significant effect of test session suggests that mice of all groups demonstrate a significant amount of freezing behavior post-FS and during the test session when compared to pre-FS freezing. In addition, a significant Test Session x Sex interaction suggests that female mice exhibited a higher percentage of freezing compared to male mice in the post-FS session (S6 Fig). Across each group, there were mice that showed little to no freezing post-FS or during the test session, suggesting that *Ywhae* CKO and dFlC mice can show a varying degree of response to the fear stimulus.

The T-maze is a commonly used test of spatial working memory in rodents. Interestingly, dysfunctions in the hippocampus and cortex have been linked to altered T-maze performance [56]. To test whether *Ywhae* CKO affects spatial working memory, animals were allowed to freely locomote for 15-minutes in a 3-armed maze with unique visual cues at the end of each arm. The total number of alternations (3 consecutive arm entries) and the percent correct alternations (3 consecutive entries into different arms) were measured to assess spatial working memory. A correct alternation reflects the animal’s ability to remember which arms were most recently visited. There was no genotype effect in percent correct alternations nor total number of alternations. Sex was not a significant factor in percent correct alternations but neared a main effect that would have suggested that males display a higher percent correct alternations (% correct, Two-way ANOVA; Genotype: F(1, 88)=0.1560, p = 0.6938; Sex: F(1, 88)=3.615, p = 0.0605; Interaction: F(1, 88)=1.070, p = 0.3039) (Fig 5D). However, there was a main effect of sex in total number of alternations, suggesting females performed a higher number of alternations (total #, Two-way ANOVA; Genotype: F(1, 88)=0.05646, p = 0.8127; Sex: F(1, 88)=9.22, p = 0.0026**; Interaction: F(1, 88)=0.008117, p = 0.9284) (Fig 5E).

These data indicate that *Ywhae* knockout in forebrain glutamatergic neurons alone does not significantly impact locomotor activity, anxiety behavior, working memory, or associative memory performance. However, sex differences occur in this *Ywhae* knockout model, highlighting the need to include males and females in all 14-3-3 knockout studies and the importance of sex specific analyses.

### Assessment of sensorimotor gating abilities

The regulation of sensory information that allows for normal cognition is referred to as sensorimotor gating. Sensorimotor gating deficits are a commonly observed phenotype in schizophrenia patients [57]. The pre-pulse inhibition (PPI) of a startle response is a measure of sensorimotor gating that can be used in both humans and animal models. Previously, we have shown that transgenic and viral induced 14-3-3 FKO mice exhibit a reduction in PPI compared to wildtype controls [10,16]. However, heterozygous *Ywhae* knockout mice did not significantly differ from controls in the PPI test [25].

We measured animals’ startle response following a 90dB acoustic stimulus, both with and without a pre-pulse stimulus of 67, 70, 72, or 76dB. Pre-pulse inhibition (PPI) occurs when an animal exhibits a smaller starter response to an acoustic stimulus when a pre-pulse stimulus is presented first. In this study, we observed increased PPI in *Ywhae* CKO mice compared to their dFlC littermates across all pre-pulse intensities (Two-way RM ANOVA; Pre-Pulse Intensity: F(2.269, 31.76)=1.307, Geisser-Greenhouse’s epsilon = 0.7563, p = 0.2869; Genotype: F(1, 14)=4.71, p = 0.0477*; Subject: F(14, 42)=2.289, p = 0.0195*; Pre-Pulse Intensity x Genotype: F(3, 42)=0.1785, p = 0.9104) (Fig 5F). However, the high subject variability indicated by the significant main effect of subject may limit this conclusion. Interestingly, wildtype C57BL/6J mice displayed an average of 50–70% PPI in our previous studies [10,12]. Here, using the same protocol, the PPI observed in *Ywhae* dFlC mice was on average between 27–41% while the PPI observed for *Ywhae* CKO mice was on average 43–52%. Thus, CKO mice show increased PPI compared to dFlC mice. The difference in behavioral phenotype between wildtype C57BL/6J mice and dFlC mice, which come from transgenic C57BL/6J backgrounds, suggests that the *Ywhae* dFlC and CKO mice cannot be directly compared with wildtype controls.

### Evaluation of social behavior

The three-chamber sociability and social novelty tests are used in rodent models as correlates of asociality, a negative symptom of schizophrenia [58–60]. When given the choice to spend time in a chamber with an unfamiliar conspecific animal and an empty chamber, rodents typically prefer the novel animal (sociability). Further, when given the choice to spend time in a chamber with a familiar conspecific animal and an unfamiliar conspecific animal, again rodents typically prefer the novel animal (social novelty). The 14-3-3 FKO mice, which have all seven 14-3-3 isoforms functionally knocked out in the forebrain, exhibit decreased sociability and no social novelty preference compared to C57BL/6J wildtype controls, as measured by time spent in each chamber [10]. Heterozygous *Ywhae* knockout mice from a 129/S6 x NIH Black Swiss background show no difference in sociability compared to controls [25], while homozygous *Ywhae* knockouts from a mixed 129/SVE x C57BL/6J background show increased sociability compared to controls [43]. It is not surprising to see strain specific differences in the three-chamber social interaction tests across *Ywhae* knockout studies, as strain differences in this test have been observed by others [61].

**Fig 6:**
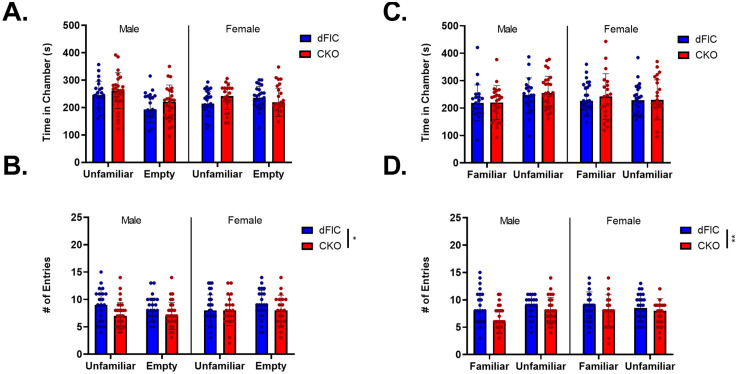
Social behavior evaluation. Neither genotype nor sex have a main effect on time spent in a chamber with an unfamiliar conspecific and an empty chamber (Sociability; CKO-male: N = 27, CKO-female: N = 21, dFlC-male: N = 21, dFlC-female: N = 25) **(A)**. A significant genotype effect suggests CKO mice perform less chamber entries overall compared to dFlC mice (Sociability; CKO-male: N = 27, CKO-female: N = 21, dFlC-male: N = 21, dFlC-female: N = 25) **(B)**. Neither genotype nor sex have a main effect on time spent in a chamber with a familiar conspecific and an unfamiliar conspecific (Social novelty; CKO-male: N = 27, CKO-female: N = 21, dFlC-male: N = 21, dFlC-female: N = 25) **(C)**. A significant genotype effect suggests CKO mice perform less chamber entries overall compared to dFlC mice (Social novelty; CKO-male: N = 26, CKO-female: N = 21, dFlC-male: N = 21, dFlC-female: N = 25) **(D)**.

In this study, we found that neither CKO nor dFlC mice showed preference for sociability, exhibiting no statistically significant difference in the amount of time spent in a chamber with a novel conspecific and an empty chamber (Time, Three-way ANOVA; Genotype: F(1, 90)=3.16, p = 0.0788; Sex: F(1, 90)=0.03729, p = 0.8473; Chamber: F(1, 90)=2.655, p = 0.1067; Chamber x Genotype: F(1, 90)=0.1067, p = 0.7447; Chamber x Sex: F(1, 90)=7.035, p = 0.0094**; Genotype x Sex: F(1, 90)=0.1782, p = 0.6739; Chamber x Genotype x Sex: F(1, 90)=0.1958, p = 0.6592) (Fig 6A). An interaction plot investigating the significant Chamber x Sex interaction reveals that there is a crossover interaction between the Camber and Sex (S7 Fig). Further, there was a significant genotype main effect in the amount of entries into each chamber, suggesting CKO mice performed less chamber entries than dFlC mice regardless of chamber (Entries, Three-way ANOVA; Genotype: F(1, 90)=5.207, p = 0.0249*; Sex: F(1, 90)=0.2404, p = 0.6251; Chamber: F(1, 90)=0.01341, p = 0.9081; Chamber x Genotype: F(1, 90)=0.006722, p = 0.9348; Chamber x Sex: F(1, 90)=0.9896, p = 0.3225; Genotype x Sex: F(1, 90)=0.5124, p = 0.4760; Chamber x Genotype x Sex: F(1, 90)=0.5247, p = 0.4707) (Fig 6B).

Furthermore, neither CKO nor dFlC mice showed preference for social novelty. Mice exhibited no statistically significant difference in the amount of time spent in a chamber with a familiar conspecific and an unfamiliar conspecific (Time, Three-way ANOVA; Genotype: F(1, 90)=1.481, p = 0.2267; Sex: F(1, 90)=0.2525, p = 0.6166; Chamber: F(1, 90)=1.404, p = 0.2392; Chamber x Genotype: F(1, 90)=0.6233, p = 0.4319; Chamber x Sex: F(1, 90)=2.203, p = 0.1412; Genotype x Sex: F(1, 90)=0.6015, p = 0.4400; Chamber x Genotype x Sex: F(1, 90)=0.07167, p = 0.7895) (Fig 6C). Consistent with the Sociability test, there was a main effect of genotype in the number of entries into each chamber with a familiar conspecific and an unfamiliar conspecific, suggesting CKO mice performed less entries overall (Entries, Three-way ANOVA; Genotype: F(1, 89)=7.380, p = 0.0079**; Sex: F(1, 89)=0.06934, p = 0.7929; Chamber: F(1, 89)=0.6024, p = 0.4397; Chamber x Genotype: F(1, 89)=1.369, p = 0.2452; Chamber x Sex: F(1, 89)=1.843, p = 0.1780; Genotype x Sex: F(1, 89)=0.1244, p = 0.7251; Chamber x Genotype x Sex: F(1, 89)=0.3.622, p = 0.0603) (Fig 6D). In addition, there was a significant interaction effect between genotype and age, with CKO and dFlC mice that are 2–3 months of age showing a crossover interaction (S8 Fig).

While genotype did not influence the time spent in each chamber, CKO mice showed less chamber entries overall compared to dFlC mice. Collectively, these results suggest that the knockout of *Ywhae* in glutamatergic forebrain neurons does not significantly impact social behavior but may influence the level of locomotor activity in this assay. However, it is important to note that wildtype C57BL/6J mice display preferences for sociability and social novelty. The lack of sociability and social novelty displayed by our control dFlC mice is inconsistent with the social behaviors we would expect from a control group. We compared the social behavior of a separate group of dFlC mice to a group of wildtype C57BL/6J mice and found that dFlC mice significantly differed from wildtype controls and again did not display sociability preference (S9 Fig). This may indicate that the behaviors observed in this study may be influenced by factors other than the conditional knockout of 14-3-3ε, such as the mouse strains or the Cre/loxP targeting scheme used to generate the *Ywhae* conditional knockout model.

### Comparison of the efficiency of viral difopein expression directed by a CaMKIIα promoter versus a DIO promoter

Both transgenic and viral 14-3-3 FKO mice exhibit significantly increased novelty-induced locomotor behavior in the OFT, a response that is correlated to the positive symptoms of schizophrenia [10,16]. In the viral 14-3-3 FKO model, we used a difopein expressing virus with a CaMKIIα promoter to target CaMKIIα positive forebrain neurons for 14-3-3 FKO in wildtype mice. Thus, our previous results suggest that modulation of 14-3-3 proteins in CaMKIIα neurons can affect locomotor activity. However, in this study 14-3-3ε CKO is achieved via Cre/loxP recombination in a subset of Cre expressing CaMKIIα positive neurons in mice from a T29-1 background, and *Ywhae* CKO mice did not show the same hyperlocomotor phenotype in the OFT. We reasoned that there are three key differences between the 14-3-3 FKO model and the *Ywhae* CKO model that may explain the differing locomotor activity results; the knockout of all seven 14-3-3 isoforms versus the knockout of only 14-3-3ε, the use of genetic versus viral methodology used to achieve 14-3-3 knockout, and the use of promoter driven expression versus Cre/loxP recombination driven expression. To test whether the behavioral difference between the two models could have been affected by the efficiency of promoter driven versus Cre/loxP recombination driven expression, we compared two viral strategies for cell type specific difopein expression, Cre/loxP recombination in a CaMKIIα-Cre transgenic mouse line and direct targeting with a CaMKIIα promoter.

**Fig 7:**
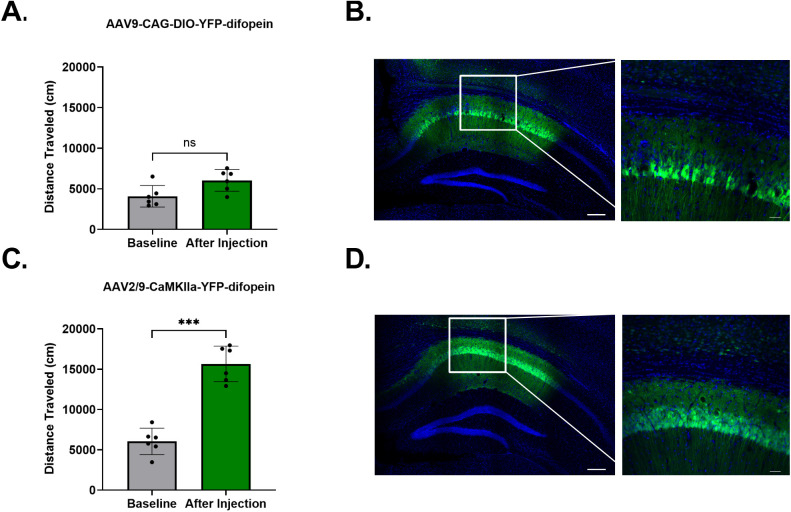
Comparison of two viral 14-3-3 knockout strategies. Virally expressed difopein mediated 14-3-3 FKO in the CA1 is more efficient when directed by a CaMKIIα promoter in wildtype mice than when directed by Cre/loxP recombination in T29-1 mice. T29-1 mice do not show a change from baseline in distance traveled during a 30 minute OFT session after bilateral injection of AAV9-CAG-DIO-YFP-difopein into the CA1 (N = 6) **(A)**. Wildtype mice show a significant increase from baseline in distance traveled during a 30 minute OFT session after bilateral injection of AAV2/9-CaMKIIa-YFP-difopein into the CA1 (N = 6) **(C)**. Representative 5x tile images (scale bar = 200 µm) and enlarged regions of interest (ROI) (scale bar = 50 µm) of the CA1 of T29-1 (B) and wildtype (D) mice.

After completing a baseline OFT session, we injected an AAV9-CAG-DIO-YFP-difopein virus into the CA1 of T29-1 C57BL/6J mice, and we injected an AAV2/9-CaMKIIα-YFP-difopein virus into the CA1 of wildtype C57BL/6J mice. We then compared their locomotor behavior in a second OFT session two-weeks following viral injection. In line with our previously published results, the AAV2/9-CaMKIIα-YFP-difopein virus induced a significant increase in locomotor activity in wildtype C57BL/6J mice (paired t-test, t = 10.87, df = 5, p = 0.0001**) (Fig 7C). However, no significant change in locomotor activity was observed in the T29-1 C57BL/6J mice that received the AAV9-CAG-DIO-YFP-difopein virus (paired t-test, t = 2.29, df = 5, p = 0.0706) (Fig 7A). Although fluorescence imaging showed that difopein is expressed throughout the CA1 in both groups, expression appears to be less robust in the mice that received AAV9-CAG-DIO-YFP-difopein. Suggesting that Cre/loxP recombination driven expression was less efficient compared to promoter driven expression (Fig 7B, 7D). Thus, it is possible that the lower efficiency strategy of using Cre/loxP recombination to induce the conditional knockout of 14-3-3ε in the *Ywhae* CKO mouse model may have influenced the behavioral results observed in this study.

## Discussion

In this study, we reported the behavioral analyses of a mouse model in which 14-3-3ε is conditionally knocked out in a subset of CaMKIIα positive forebrain neurons. Our previous work shows that the functional inhibition of all 14-3-3 isoforms in this neuronal population leads to several behavioral alterations associated with psychiatric disorders; including increased distance traveled in the OFT, decreased percent correct alternations in the T-maze, decreased sociability and social novelty preferences in the 3-chamber social interactions tests, decreased PPI response, and enhanced freezing response in the fear conditioning test [10,12,16]. We have also shown that 14-3-3 deficiency, specifically in the forebrain pyramidal neurons, leads to both hippocampal hyperactivity and increased dopamine release from the ventral tegmental area (VTA), phenotypes observed in human schizophrenia patients [17]. However, all seven isoforms of 14-3-3 are non-specifically inhibited in the 14-3-3 FKO model, therefore we are unable to differentiate the role of individual isoforms in the observed phenotypes. Although the 14-3-3 isoforms may share some binding partners and functions, the fact that each isoform is encoded by a unique gene and that there is variation in both the regional distribution and timing of their expression suggests that there are also isoform specific functions.

We chose to specifically examine the role of 14-3-3ε in psychiatric related behaviors because of the implication of *YWHAE* mutations in human neurodevelopmental and psychiatric conditions, as well as the impact of 14-3-3ε deficits on murine behavior and cortical/hippocampal development shown in previous animal models. Based on our 14-3-3 viral FKO model, we conditionally knocked out 14-3-3ε in CaMKIIα-Cre positive forebrain pyramidal neurons [49]. Although our behavioral battery consists of tests widely used to study schizophrenia-like behavior and was sensitive enough to detect subtle sex differences, we did not see a genotype difference between knockout and control animals in many of the behaviors investigated here. Further, our results differed from what would be expected based on previous *Ywhae* knockout studies. Interestingly, in the social behavior and sensorimotor gating tests, where we observed significant behavioral differences between *Ywhae* CKO mice and their dFlC control littermates, the results were discordant with the behavior typically observed in C57BL/6J wildtype mice. This may suggest that something other than 14-3-3ε CKO may be affecting behavior in this model. In our opinion, several experimental and molecular factors may have contributed to these results. These include the mouse strain used, the timing and extent of 14-3-3ε knockout strategy, the Cre/loxP targeting scheme, and compensation by other 14-3-3 isoforms. The consideration for each of these factors will be discussed below.

It is well-known that mouse models from different genetic backgrounds can show strain specific differences in behavioral tests [62,63]. Thus, the mouse strain used in a study can impact the results, and intra-strain differences limit our ability to interpret a body of research that utilizes multiple strains. In other *Ywhae* knockout models, mice are from mixed 129/S6 x NIH Black Swiss [40], 129/SVE x C57BL/6J[43], or C57BL/6N x 129S5/SvEvBrd/Wtsi backgrounds [64]. As was discussed above, homozygous mice could not always be produced in these models and heterozygous mice were often used for behavioral analyses. Further, sub-strains of C57BL/6 mice can also show inconsistent behavioral results and mismatching sub-strains can confound behavioral analyses [65–67]. When we compared our *Ywhae* dFlC mice to wildtype C57BL/6J, they showed inconsistent social behavior preferences. Supporting the conclusion that our dFlC mice cannot be viewed as a true control group akin to using wildtype mice. Although each of the three mouse lines used in the creation of the *Ywhae* CKO model are from a C57BL/6J background, different sub-strains may have contributed to their genetic makeup and altered the behavioral phenotype. This factor may reduce the validity of a direct comparison between any one of the previous *Ywhae* studies and our *Ywhae* CKO model and may explain why our control dFlC mice showed unexpected behaviors as well.

In addition, the timing and extent of 14-3-3ε knockout varies among different studies. In our model, a conditional knockout allele was created and used to target a specific subset of neurons in a defined region of the brain with the CaMKIIα promoter. Because this promoter is expressed following birth, the resulting animals go through normal prenatal neurodevelopment and any resulting behavioral differences can be attributed to the knockout of 14-3-3ε in CaMKIIα positive forebrain neurons. In previous models, a knockout allele was targeted in embryonic stem cells for germline transmission, effectively knocking out *Ywhae* throughout the body from the beginning of development [40,64]. The resulting animals show neurodevelopmental defects and often do not survive to adulthood. Therefore, any behavioral difference observed in adult mice cannot be attributed to specific brain areas or cell types, and we cannot discern whether the differences are due to improper development or the lack of functional 14-3-3ε protein in mature neurons. Thus, it is difficult to compare the behavioral results observed here to previous *Ywhae* knockout studies.

Another factor that may have influenced our results is the Cre/loxP system that was used to conditionally knockout *Ywhae* in CaMKIIα positive neurons. There are several commonly used CaMKIIα-Cre driver mouse lines, including the CaMKIIα-iCre, nestin-Cre, and CaMKIIα-Cre T29-1 lines. Due to the toxicity associated with high Cre expression, CaMKIIα-iCre and nestin-Cre mice show significantly increased levels of microglia marker Iba1 and astrocytic protein GFAP in the hippocampus, indicative of neuroinflammation, as well as changes in expression of synaptic proteins [68]. CaMKIIα-Cre T29-1 mice, on the other hand, are more similar to wildtype mice in these measures, as the Cre expression in this line is lower compared to the CaMKIIα-iCre and nestin-Cre lines [68]. When crossed with a lacZ reporter mouse line, T29-1 mice show 98% Cre/loxP recombination efficiency in the CA1 region of the hippocampus, as measured by the percentage of X-gal positive cells [49]. However, it is possible that either the recombination efficiency was lower in our hands due to breeding factors [69], or that manipulating the group of CaMKIIα neurons that have Cre expression in T29-1 mice is not sufficient to cause significant behavioral alterations. To begin exploring this possibility, we compared the locomotor behavior following functional inhibition of all 14-3-3 proteins using a AAV2/9-CaMKIIα-YFP-difopein virus in wildtype mice and a AAV9-CAG-DIO-YFP-difopein virus in T29-1 mice. Virally induced difopein expression directed by the CaMKIIα promoter in wildtype mice was sufficient to induce hyperlocomotor behavior in the OFT, while no significant change was observed in the behavior of T29-1 mice that had difopein expression directed by Cre/loxP recombination. Although YFP-difopein expression is noted throughout the CA1 of both groups in this experiment, it appears as though less neurons fluoresce green in the T29-1 mice, suggesting a less efficient 14-3-3 FKO. It is possible that using the DIO construct to direct difopein expression in T29-1 mice restricts 14-3-3 FKO to the subset of CaMKIIα neurons that express Cre, whereas using the CaMKIIα promoter to direct difopein expression in wildtype mice reaches a broader population of CaMKIIα positive neurons. Recently, we have shown that difopein induced locomotor hyperactivity and increased VTA dopamine release is mediated by glutamatergic CA1 neurons that project to the lateral septum (LS) [17]. Therefore, the subset of Cre positive CaMKIIα neurons in the CA1 of T29-1 mice may not overlap significantly with the population of glutamatergic neurons that mediate these phenotypes. This could partially explain why we did not observe behavioral alterations, particularly locomotor hyperactivity, following *Ywhae* CKO or 14-3-3 FKO in T29-1 mice.

Finally, the lack of significant differences in several behaviors between CKO and dFlC animals in this study may be explained by other 14-3-3 isoforms compensating for the loss of 14-3-3ε. Previous studies have shown that mammalian 14-3-3ε readily forms heterodimers with the β, η, γ, and ζ 14-3-3 isoforms, but did not observe significant 14-3-3ε homodimer formation [70,71]. Neuronal functions dependent on 14-3-3ε heterodimers would be less severely affected by *Ywhae* CKO than functions of 14-3-3ε homodimers, as there is redundancy in the roles of each 14-3-3 isoform. For example, one of the major roles of 14-3-3ε is in the promotion of surface expression of NMDA receptors. While initial studies highlighted the specific interaction between 14-3-3ε and the GluN2C subunit [72], it was later shown that all 14-3-3 isoforms other than 14-3-3σ could bind to the same Ser1096 residue of GluN2C [73]. Thus, it is possible that the knockout of 14-3-3ε in one anatomical location/ cell type is not sufficient to induce significant behavioral changes. 14-3-3ε and 14-3-3ζ both show relatively high expression in the forebrain and share some of the same binding partners [74]. Interestingly, in a 14-3-3ε/ 14-3-3ζ double knockout mouse model, double knockout mice show more severe defects than single knockout mice [47]. Further, we have shown that the complete FKO of 14-3-3 proteins in CaMKIIα neurons of the CA1 causes significant molecular and behavioral disturbances. Therefore, it may be that in this context multiple 14-3-3 isoforms will have to be lost in glutamatergic forebrain neurons before a behavioral alteration is observed. Others have found that 14-3-3 isoforms can interact with binding partners in a context-specific manner. For example, 14-3-3η co-precipitates with the Maged1 protein in glutamatergic neurons of the thalamus specifically following cocaine administration [75]. Although we did not observe behavioral changes following the loss of 14-3-3ε in CaMKIIα glutamatergic neurons of the CA1 in this study, it remains a possibility that results would differ under a different context. Thus, more work studying isoform-specific roles of 14-3-3 proteins across different brain regions, neuronal population, and contexts would provide valuable insight to a range of neuropsychiatric conditions.

Although we did not observe robust behavioral differences within this model, we did find sex differences in several of our experiments. Historically, there has been a lack of inclusion of female subjects in both human and animal based biomedical research. In a survey of neuroscience studies published in 2017; just 52% of papers reported including both male and female subjects, 16% of papers did not report which sex was used, and only 15% reported assessing sex as a biological variable in the analyses [76]. In fact, many of the 14-3-3 animal model studies done in the past only included male subjects while those that included males and females did not assess potential sex differences. There are known to be sex differences in both the development and presentation of schizophrenia in humans [77]. Therefore, including sex as a biological variable in animal model research is important to strengthen the translatability to humans. Thus, we included both male and female mice in each of our experimental groups so that we could determine if sex played a role in our findings. We observed a significant difference between male and female mice in the OFT and T-maze tests. Each of these tests have been used in previous 14-3-3 knockout models. Without the analyses of sex as a biological variable, it remains uncertain whether sex influenced the behavioral results of past *Ywhae* knockout studies. Sex is therefore just as important a consideration as genetic strain is when analyzing the results of a study and comparing them to similar studies in the field. The results reported here highlight the need to include both males and females and to analyze sex as a biological variable in animal models of schizophrenia.

## Conclusions

Both human and animal studies have provided evidence linking the 14-3-3 proteins to several psychiatric conditions, making the 14-3-3 knockout models valuable tools to study the underlying neural mechanisms of disease. Here, we created an isoform, region, and cell-type specific mouse model in which 14-3-3ε is conditionally knocked out of the mouse forebrain glutamatergic neurons and assessed the resulting behavioural endophenotypes. Interestingly, knockout mice did not significantly differ from controls in many of the behavioural measures that were studied. Our data were unexpected given the results seen in previous 14-3-3 knockout models. While we discussed several factors that may have contributed to our results, we hypothesize that the genetic background and Cre/loxP targeting scheme of our *Ywhae* CKO model respectively influence the unexpected behavioural phenotype of our dFlC group and lack of behavioural difference following 14-3-3ε conditional knockout. These conclusions are supported by our comparisons of dFlC and wildtype social behaviour as well as our viral targeting strategies. We believe that these technical limitations of our model prevent proper assessment of whether the loss of 14-3-3ε in CaMKIIα forebrain neurons contributes to schizophrenia-like behavioural phenotypes. However, this study contributes to an important discourse of how the strategies used to create an animal model can influence the results observed, how we compare the results received from similar yet distinct models, and the importance of analysing sex as biological variable. In the future, continued generation, and study of *Ywhae* models may help us better understand the role of 14-3-3ε in the healthy and disordered brain.

## Supporting information

S1 Table Summary of phenotypes associated with 14-3-3 knockout animal models. Adapted from (Navarrete, et al. 2022) (18).
(DOCX)

S1 Raw Images Annotated raw images of PCR gels. Uncropped images of the PCR gels shown in Fig. 1 are annotated to show molecular weight markers, sample loading order, sample identity, and which figure panel was generated from the image. Images were taken using the Bio-Rad Molecular Imager Gel Doc XR System and Quantity One software (version 4.6.5).
(PDF)

S1 Fig Analysis of mean fluorescence intensity. Means are plotted in units of Arbitrary Fluorescence Units (AFU). Brain slices from dFlC and CKO mice underwent IHC for co-labeling of either CaMKIIα/ 14-3-3ζ (dFlC N = 17, CKO N = 17) (A, B) or CaMKIIα/ 14-3-3ε (dFlC N = 10, CKO N = 7) (C, D). 20x fluorescence images from the CA1, CA3, DG, and mPFC were taken and the mean fluorescence intensity was measured for each image. While there was a slight statistically significant difference in CaMKIIα expression between dFlC and CKO mice, this difference is likely non-meaningful as suggested by a p-value of 0.046 (A). Expression of 14-3-3ζ was consistent across dFlC and CKO mice (B). CaMKIIα expression was consistent across dFlC and CKO mice (C), while there was a significant reduction in 14-3-3ε expression in CKO mice compared to dFlC mice (D). These results are consistent with the isoform specific conditional knockout of 14-3-3ε in our CKO model.
(DOCX)

S2 Fig Split and merged representative IHC images. Representative fluorescence images used in Fig. 3 are shown split by color channel and merged (scale bar = 50µm). Brain slices from a dFlC (A) and a CKO (B) animal were co-labeled with dapi (blue), CaMKIIα (red), and 14-3-3ζ (green). Brain slices from a dFlC (C) and a CKO (D) animal were co-labeled with dapi (blue), CaMKIIα (red), and 14-3-3ε (green).
(DOCX)

S3 Fig Acclimation in the Open Field Test. Distance traveled during three time-bins, consisting of 10-minute periods, is plotted to determine if animals acclimate to the test arena and locomote differently as time goes on. There is a significant difference in distance traveled across the different time-bins, indicating that animals locomote less as time goes on. However, there is neither a sex nor genotype difference. (Three-way ANOVA; Genotype: F(1, 62)=0.6717, p = 0.4156; Sex: F(1, 62)=1.498, p = 0.2255; Time Bin: F(1.820, 112.8)=7870, Geisser-Greenhouse’s epsilon = 0.9099, p < 0.0001****; Time Bin x Genotype: F(2, 124)=0.5571, p = 0.5743; Time Bin x Sex: F(2, 124)=0.7497, p = 0.4746; Genotype x Sex: F(1, 62)=0.005178, p = 0.9429; Chamber x Genotype x Sex: F(2, 124)=0.3429, p = 0.7104) (CKO-male: N = 19, CKO-female: N = 16, dFlC-male: N = 14, dFlC-female: N = 17).
(DOCX)

S4 Fig Effect of age on rearing activity. There was a significant main effect of sex in vertical rearing events, with males exhibiting a higher number of vertical counts than females. Further, there was a main effect of age, with mice 3–4 months of age exhibiting a higher number of vertical counts than mice 2–3 months of age (CKO-male: N = 19, CKO-female: N = 16, dFlC-male: N = 14, dFlC-female: N = 19).
(DOCX)

S5 Fig Evaluation of thigmotaxis. While no genotype difference was observed in the ratio of time spent at the Edge over the time spent in the Center of the arena in the 30-minute OFT, there was a significant sex difference, indicating that females spent more time at the edge of the arena (ratio calculated as [(Time spent at the edge)/ (Time spent in the center)]) (Two-way ANOVA; Genotype: F(1, 38)=3.117, p = 0.0855; Sex: F(1, 38)=4.964, p = 0.0319*; Interaction: F(1, 38)=0.4455, p = 0.5085) (CKO-male: N = 9, CKO-female: N = 11, dFlC-male: N = 8, dFlC-female: N = 14).
(DOCX)

S6 Fig An interaction plot investigating the significant Test Session x Sex interaction in the Fear Conditioning test. Means are plotted along with 95% confidence intervals. There is a crossover interaction between the Test Session and Sex.
(DOCX)

S7 Fig An interaction plot investigating the significant Chamber x Sex interaction in the Sociability Test. Means are plotted along with 95% confidence intervals. There is a crossover interaction between the Camber and Sex.
(DOCX)

S8 Fig Interaction plots investigating the significant Genotype x Age interaction in the Social Novelty Test. Means are plotted along with 95% confidence intervals. There is a crossover interaction between the Genotype and Age for mice 2–3 months of age (A) but not mice 3–4 months of age (B).
(DOCX)

S9 Fig Comparing dFlC social behavior to B6 controls. Because dFlC mice did not exhibit normal social behavior when compared to CKO mice, a new group of dFlC mice (N = 12) and a group of wildtype C57BL/6J mice (N = 13) were put through the sociability and social novelty tests to evaluate their social behavior. dFlC and B6 groups were age and sex matched. In the sociability test, B6 mice showed preference for an unfamiliar mouse over an empty cup, demonstrating normal sociability behavior. In alignment with the main body of this study, dFlC mice did not show a preference for sociability (Time, Two-way ANOVA; Genotype: F(1, 20)=0.5279, p = 0.2372; Chamber: F(1, 20)=7.990, p = 0.0104*; Chamber x Genotype: F(1, 20)=6.886, p = 0.0163*; Subject: F(20, 20)=0.1665, p > 0.9999) (A). While there was no main effects of Chamber or Genotype on entries performed in the sociability test, there was a highly significant variability amongst subjects (Time, Two-way ANOVA; Genotype: F(1, 20)=0.02387, p = 0.8788; Chamber: F(1, 20)=3.800, p = 0.0654; Chamber x Genotype: F(1, 20)=8.140, p = 0.0098**; Subject: F(20, 20)=4.364, p = 0.0009***) (B). In the social novelty test, B6 mice showed preference for an unfamiliar mouse over a familiar mouse, demonstrating normal social novelty behavior. However, in discordance with the main body of this study, dFlC mice also showed social novelty preference (Time, Two-way ANOVA; Genotype: F(1, 20)=0.6154, p = 0.4420; Chamber: F(1, 20)=12.57, p = 0.0020**; Chamber x Genotype: F(1, 20)=0.7032, p = 0.4116; Subject: F(20, 20)=0.1635, p > 0.9999) (C). While there was no main effects of Chamber or Genotype on entries performed in the social novelty test, there was a highly significant variability amongst subjects (Time, Two-way ANOVA; Genotype: F(1, 20)=0.8066, p = 0.3798; Chamber: F(1, 20)=2.762, p = 0.1121; Chamber x Genotype: F(1, 20)=0.7469, p = 0.3977; Subject: F(20, 20)=3.647, p = 0.0028**) (D). We then compared the social behavior results from dFlC mice in our main analysis to the new group of dFlC mice used in this experiment. The old and new groups of dFlC mice did not differ in their performance in the sociability test (Time, Two-way ANOVA; New vs Old: F(1, 56)=1.781, p = 0.1875; Chamber: F(1, 56)=0.2594, p = 0.6125; Chamber x New vs Old: F(1, 56)=0.08555, p = 0.7710; Subject: F(56, 56)=0.2536, p > 0.9999) (E). In the social novelty test, there was no main effect of New vs Old group. While the new group of dFlC mice spent a significantly shorter amount of time with a familiar mouse in the social novelty test, they did not differ from the old group of dFlC mice in the amount of time spent with an unfamiliar mouse. This may suggest that the difference between the groups of dFlC mice is influenced by the sampling of a smaller group in this experiment (Time, Two-way ANOVA; New vs Old: F(1, 57)=1.318, p = 0.2558; Chamber: F(1, 57)=6.893, p = 0.0111*; Chamber x New vs Old: F(1, 57)=5.864, p = 0.0187*; Subject: F(57, 57)=0.1448, p > 0.9999) (F).
(DOCX)

S1 File Dataset. (PDF)
