# Supplementary material for: Behavioral analyses of a forebrain glutamatergic neuron specific Ywhae conditional knockout mouse model
Source: PLoS One. 2025 Nov 11;20(11):e0335427. doi: 10.1371/journal.pone.0335427 (PMC12604760; doi:10.1371/journal.pone.0335427)
Supplement: S1 Table — Adapted from (Navarrete, et al. 2022) (18). (DOCX) [file pone.0335427.s001.docx]

| **S1 Table. Summary of phenotypes associated with 14-3-3 knockout animal models** | | | | | | | | | | |
| --- | --- | --- | --- | --- | --- | --- | --- | --- | --- | --- |
|  | | |  | |  | | |  | |  |
| **Mouse Strain / Line**  **Partial / Full Knockout**  **Age Studied** | **Behavioral Changes** | | | | | **Molecular / Synaptic / Anatomical Changes** | | | **Citation** | |
| **14-3-3ζ** | |  | |  | | |  | | | |
| SV129 / 14-3-3 ζGt(OST062)Lex  Homozygous  5-40 weeks | Hyperactive, lowered anxiety, impaired recognition memory, defect in spatial working memory, defects in sensorimotor gating | | | | | Abnormal mossy fiber navigation and glutaminergic synapse formation, 14-3-3 binding to DISC1 | | | [1] | |
| SV129 / 14-3-3 ζGt(OST062)Lex  Homozygous  30, 35 weeks | Hyperactivity rescued by clozapine, hypersensitive to amphetamine | | | | | TH preserved, reduced and mis-localized DAT | | | [2] | |
| BALB/c / 14-3-3 ζGt(OST062)Lex  Homozygous  12 weeks, 28-35 weeks | No hyperactivity, no anxiety changes, subtle learning problems, impaired spatial memory | | | | | Mis-localized hippocampal cells with aberrant connectivity, reduced spine density, normal DA signaling | | | [3] | |
| SV129 / 14-3-3 ζGt(OST062)Lex  Homozygous  28-32 weeks | Clozapine affects despair behavior in KOs, anxiety-like behavior not affected by KO or clozapine, clozapine had opposing affects in KO and WT in the Y maze | | | | | Dendritic spine defects in the hippocampus and cortex, anatomical differences not rescued by clozapine | | | [4] | |
| **14-3-3ε** |  | | | | |  | | |  | |
| Mixed (129/S6 and NIH Black Swiss)  Heterozygous / Homozygous  E 18.5 | / | | | | | Hippocampal defects, cortical thinning, neuronal migration defects, mis-localization of NDEL / LIS1 | | | [5] | |
| Mixed (129/S6 and NIH Black Swiss)  Heterozygous  9-10 weeks | Weak defect in working memory, moderately enhanced anxiety like behavior | | | | | / | | | [6] | |
| Mixed (129/S6 and NIH Black Swiss)  Heterozygous  12-15 weeks | / | | | | | Increased VMAT2 expression in the hippocampus | | | [7] | |
| Mixed (129/S6 and NIH Black Swiss)  Heterozygous  15 weeks | / | | | | | Serpentine, thin, short TH immunopositive fibers, few and sparse dendritic spine like immunopositive varices, significant decrease in TH positive fibers | | | [8] | |
| Mixed (129SVE and C57BL/6)  Heterozygous / Homozygous  12-16 weeks | Hyperactivity, decreased working memory, increased sociability | | | | | / | | | [9] | |
| Mixed (C57BL/6N and 129S5/SvEvBrd/Wtsi)  Homozygous  4-16 weeks |  | | | | | Reduced body weight, shortened skull and a concave nasal spline, microcephaly | | | [10] | |
| **14-3-3 ζ/ε** |  | | | | |  | | |  | |
| 129/SvEv  Heterozygous  E15.5-18.5 | / | | | | | Abnormal proliferation / differentiation of neuronal progenitors in culture, increased differentiation into neurons, cortical neuronal migration defects, abnormal activity of the Rho family and its effectors | | | [11] | |
|  |  |  |  |  |  |  |  |  |  |  |
| **14-3-3γ** |  | | | | |  | | |  | |
| 129SV  Heterozygous / Homozygous | Normal cage behavior | | | | | Several differentially expressed proteins, normal anatomy | | | [12] | |
| ICR outbred  In utero electroporation of shRNA inhibitor  E17-18.5, P3-15 | / | | | | | Neuronal migration delay of cerebral pyramidal neurons, thicker / highly branched leading processes, impaired ability of the leading process to enter the MZ | | | [13] | |
| C57BL/6 / B6;CBYwhagGt(pU-21W)266Card  Heterozygous  10 weeks | Hyperactivity, depressive like behavior, sensitive to acute stress | | | | | / | | | [14] | |
| **14-3-3 FKO** |  | | | | |  | | |  | |
| C57BL/6  Transgenic expressed inhibitor  12-24 weeks | Deficit in associative learning and memory | | | | | Defects in hippocampal LTP, reduced synaptic NMDARs | | | [15] | |
| C57BL/6  Transgenic expressed inhibitor  12-24 weeks | Hyperactivity, unaltered anxiety response, deficit in working memory, deficit in sensorimotor gating, social withdrawal | | | | | Cortical neurotransmission deficit, morphological alterations, reduced phospho- cofilin, increase delta catenin | | | [16] | |
| C57BL/6  AAV delivered shRNA  12-24 weeks | Behaviors recapitulated through acute 14-3-3 inhibition in the PFC and HP | | | | | / | | | [17] | |
| C57BL/6  Transgenic expressed inhibitor  12-24 weeks | / | | | | | Altered neural oscillations in theta / gamma frequency ranges | | | [18] | |
| B6.SJLSlc6a3tm 1.1(cre)Bkmn/J B6.Cg-Tg(Camk2a-cre)T29-1Stl/J  AAV delivered YFP-difopein  12-24 weeks | / | | | | | Increased activation of LS neurons is necessary for over-activation of DA neurons and psychomotor behavior induced by 14-3-3 inhibition in the dCA1 | | | [19] | |
| C57BL/6  Transgenic expressed inhibitor  12-24 weeks | / | | | | | Increased intrinsic excitability, firing pattern shift, altered action potential shape, altered calcium channel currents, and reduced dendritic complexity in CA1 pyramidal neurons | | | [20] | |

**S1 Table. Summary of phenotypes associated with 14-3-3 knockout animal models.** Adapted from [21].

**References**

1. Cheah PS, Ramshaw HS, Thomas PQ, Toyo-Oka K, Xu X, Martin S, et al. Neurodevelopmental and neuropsychiatric behaviour defects arise from 14-3-3zeta deficiency. Mol Psychiatry. 2012;17(4):451-66.

2. Ramshaw H, Xu X, Jaehne EJ, McCarthy P, Greenberg Z, Saleh E, et al. Locomotor hyperactivity in 14-3-3zeta KO mice is associated with dopamine transporter dysfunction. Transl Psychiatry. 2013;3:e327.

3. Xu X, Jaehne EJ, Greenberg Z, McCarthy P, Saleh E, Parish CL, et al. 14-3-3zeta deficient mice in the BALB/c background display behavioural and anatomical defects associated with neurodevelopmental disorders. Sci Rep. 2015;5:12434.

4. Jaehne EJ, Ramshaw H, Xu X, Saleh E, Clark SR, Schubert KO, et al. In-vivo administration of clozapine affects behaviour but does not reverse dendritic spine deficits in the 14-3-3zeta KO mouse model of schizophrenia-like disorders. Pharmacol Biochem Behav. 2015;138:1-8.

5. Toyo-oka K, Shionoya A, Gambello MJ, Cardoso C, Leventer R, Ward HL, et al. 14-3-3epsilon is important for neuronal migration by binding to NUDEL: a molecular explanation for Miller-Dieker syndrome. Nat Genet. 2003;34(3):274-85.

6. Ikeda M, Hikita T, Taya S, Uraguchi-Asaki J, Toyo-oka K, Wynshaw-Boris A, et al. Identification of YWHAE, a gene encoding 14-3-3epsilon, as a possible susceptibility gene for schizophrenia. Hum Mol Genet. 2008;17(20):3212-22.

7. Iritani S, Sekiguchi H, Habuchi C, Hikita T, Taya S, Kaibuchi K, et al. Immunohistochemical study of vesicle monoamine transporter 2 in the hippocampal region of genetic animal model of schizophrenia. Synapse. 2010;64(12):948-53.

8. Sekiguchi H, Iritani S, Habuchi C, Torii Y, Kuroda K, Kaibuchi K, et al. Impairment of the tyrosine hydroxylase neuronal network in the orbitofrontal cortex of a genetically modified mouse model of schizophrenia. Brain Res. 2011;1392:47-53.

9. Wachi T, Cornell B, Toyo-Oka K. Complete ablation of the 14-3-3epsilon protein results in multiple defects in neuropsychiatric behaviors. Behav Brain Res. 2017;319:31-6.

10. Denomme-Pichon AS, Collins SC, Bruel AL, Mikhaleva A, Wagner C, Vancollie VE, et al. YWHAE loss of function causes a rare neurodevelopmental disease with brain abnormalities in human and mouse. Genet Med. 2023;25(7):100835.

11. Toyo-oka K, Wachi T, Hunt RF, Baraban SC, Taya S, Ramshaw H, et al. 14-3-3epsilon and zeta regulate neurogenesis and differentiation of neuronal progenitor cells in the developing brain. J Neurosci. 2014;34(36):12168-81.

12. Steinacker P, Schwarz P, Reim K, Brechlin P, Jahn O, Kratzin H, et al. Unchanged survival rates of 14-3-3gamma knockout mice after inoculation with pathological prion protein. Mol Cell Biol. 2005;25(4):1339-46.

13. Wachi T, Cornell B, Marshall C, Zhukarev V, Baas PW, Toyo-oka K. Ablation of the 14-3-3gamma Protein Results in Neuronal Migration Delay and Morphological Defects in the Developing Cerebral Cortex. Dev Neurobiol. 2016;76(6):600-14.

14. Kim DE, Cho CH, Sim KM, Kwon O, Hwang EM, Kim HW, et al. 14-3-3gamma Haploinsufficient Mice Display Hyperactive and Stress-sensitive Behaviors. Exp Neurobiol. 2019;28(1):43-53.

15. Qiao H, Foote M, Graham K, Wu Y, Zhou Y. 14-3-3 proteins are required for hippocampal long-term potentiation and associative learning and memory. J Neurosci. 2014;34(14):4801-8.

16. Foote M, Qiao H, Graham K, Wu Y, Zhou Y. Inhibition of 14-3-3 Proteins Leads to Schizophrenia-Related Behavioral Phenotypes and Synaptic Defects in Mice. Biol Psychiatry. 2015;78(6):386-95.

17. Graham K, Zhang J, Qiao H, Wu Y, Zhou Y. Region-specific inhibition of 14-3-3 proteins induces psychomotor behaviors in mice. NPJ Schizophr. 2019;5(1):1.

18. Jones ZB, Zhang J, Wu Y, Zhou Y. Inhibition of 14-3-3 Proteins Alters Neural Oscillations in Mice. Front Neural Circuits. 2021;15:647856.

19. Zhang J, Navarrete M, Wu Y, Zhou Y. 14-3-3 dysfunction in the dorsal hippocampus CA1 (dCA1) induces psychomotor behavior via a dCA1-lateral septum-ventral tegmental area pathway. Frontiers in Molecular Neuroscience. 2022:(In Press).

20. Logue JB, Vilmont V, Zhang J, Wu Y, Zhou Y. Inhibition of 14-3-3 proteins increases the intrinsic excitability of mouse hippocampal CA1 pyramidal neurons. Eur J Neurosci. 2024.

21. Navarrete M, Zhou Y. The 14-3-3 Protein Family and Schizophrenia. Frontiers in Molecular Neuroscience. 2022;15.
