# Supplementary figures and images for: Behavioral analyses of a forebrain glutamatergic neuron specific Ywhae conditional knockout mouse model
Source: PLoS One. 2025 Nov 11;20(11):e0335427. doi: 10.1371/journal.pone.0335427 (PMC12604760; doi:10.1371/journal.pone.0335427)

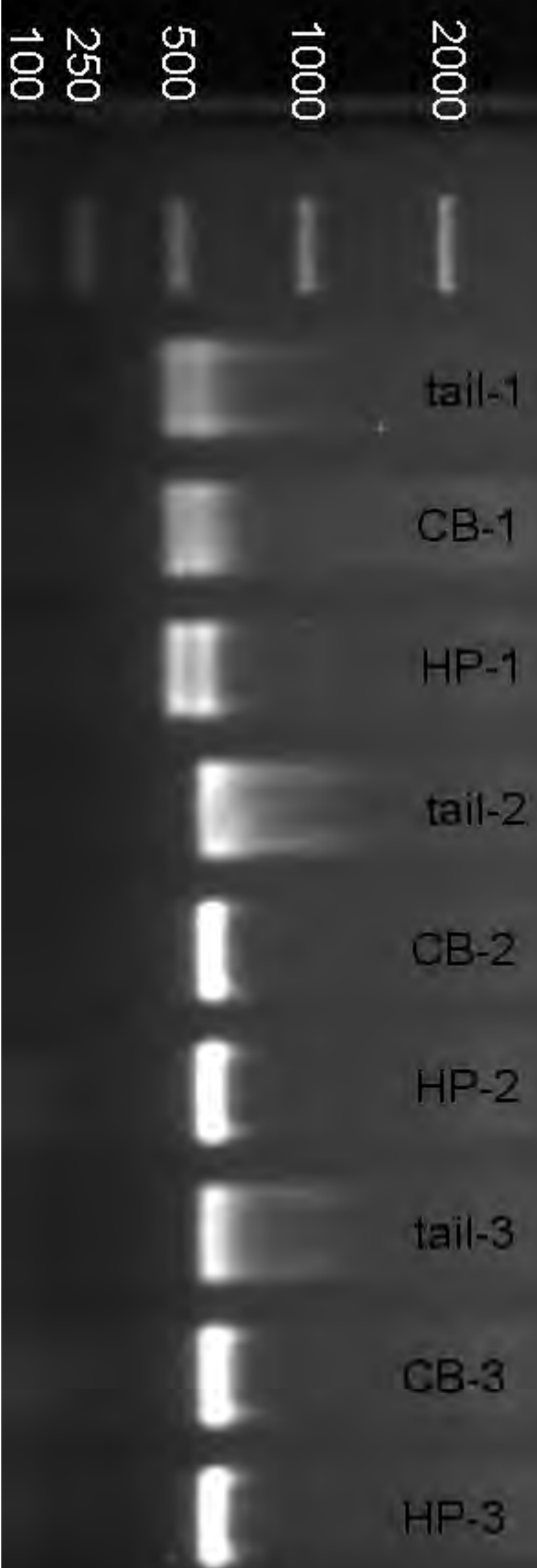

Figure 1B  
F/R Primers

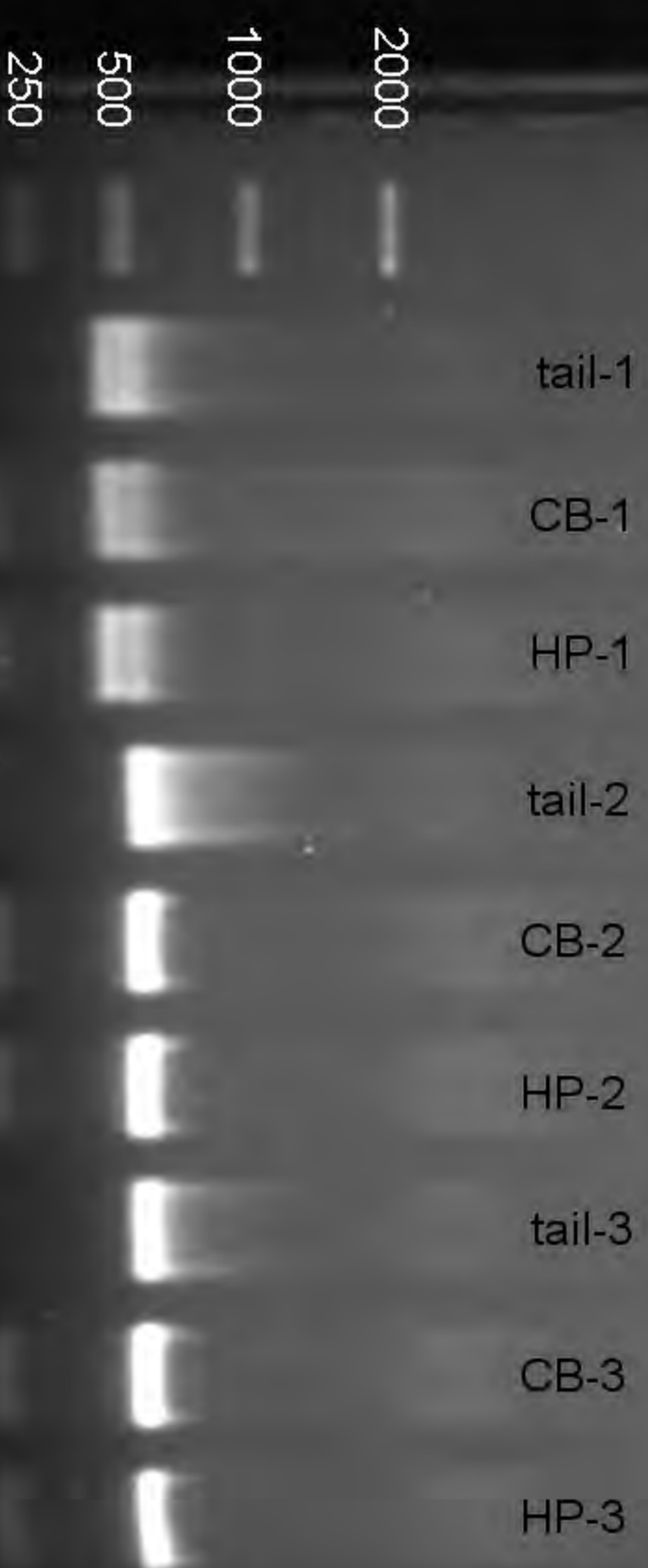

Figure 1B  
P1/P2 Primers

Figure 1D

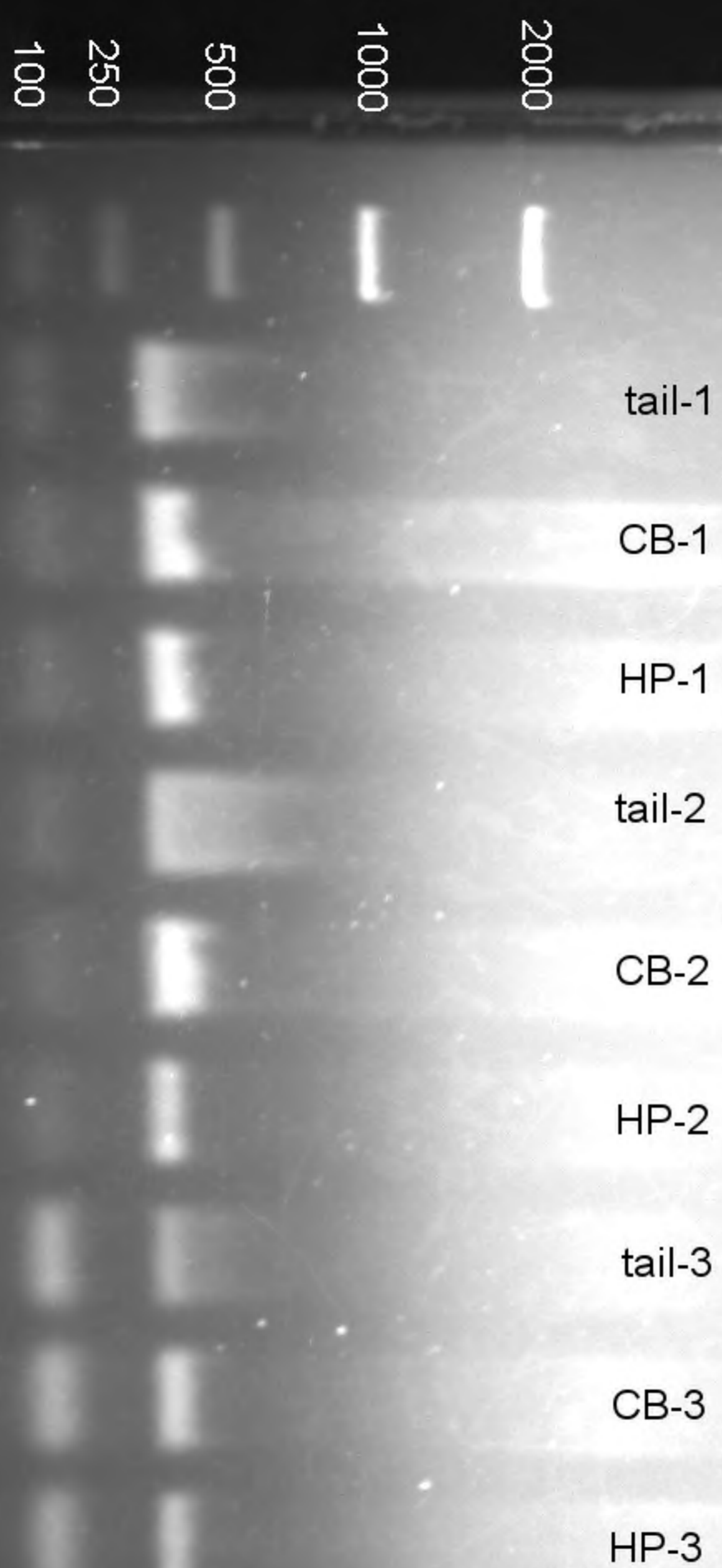

Supplement: S1 Raw Images — Uncropped images of the PCR gels shown in Fig. 1 are annotated to show molecular weight markers, sample loading order, sample identity, and which figure panel was generated from the image. Images were taken using the Bio-Rad Molecular Imager Gel Doc XR System and Quantity One software (version 4.6.5). (PDF) [file pone.0335427.s002.pdf]
