# Supplementary material for: Behavioral analyses of a forebrain glutamatergic neuron specific Ywhae conditional knockout mouse model
Source: PLoS One. 2025 Nov 11;20(11):e0335427. doi: 10.1371/journal.pone.0335427 (PMC12604760; doi:10.1371/journal.pone.0335427)
Supplement: S1 Fig — Means are plotted in units of Arbitrary Fluorescence Units (AFU). Brain slices from dFlC and CKO mice underwent IHC for co-labeling of either CaMKIIα/ 14-3-3ζ (dFlC N = 17, CKO N = 17) (A, B) or CaMKIIα/ 14-3-3ε (dFlC N = 10, CKO N = 7) (C, D). 20x fluorescence images from the CA1, CA3, DG, and mPFC were taken and the mean fluorescence intensity was measured for each image. While there was a slight statistically significant difference in CaMKIIα expression between dFlC and CKO mice, this difference is likely non-meaningful as suggested by a p-value of 0.046 (A). Expression of 14-3-3ζ was consistent across dFlC and CKO mice (B). CaMKIIα expression was consistent across dFlC and CKO mice (C), while there was a significant reduction in 14-3-3ε expression in CKO mice compared to dFlC mice (D). These results are consistent with the isoform specific conditional knockout of 14-3-3ε in our CKO model. (DOCX) [file pone.0335427.s003.docx]

**A.**

| **Brain Region** | **Dapi** | **14-3-3ζ** | **CaMKIIα** | **Merge** |
| --- | --- | --- | --- | --- |
| **mPFC** | **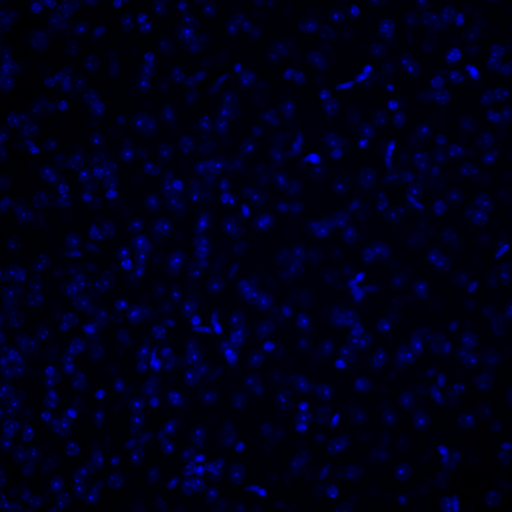** | **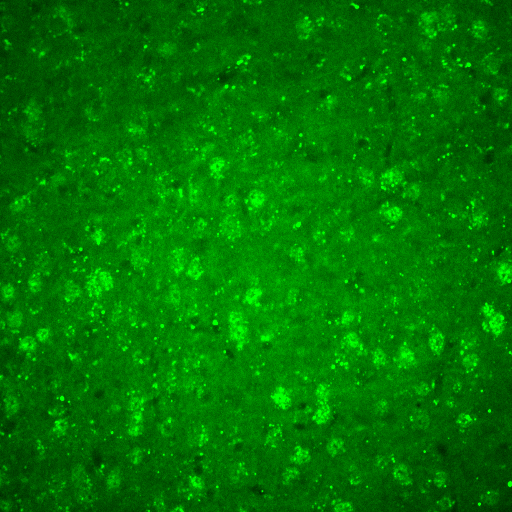** | **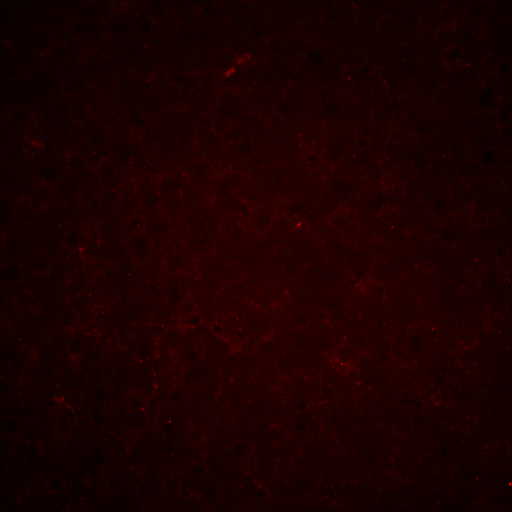** | **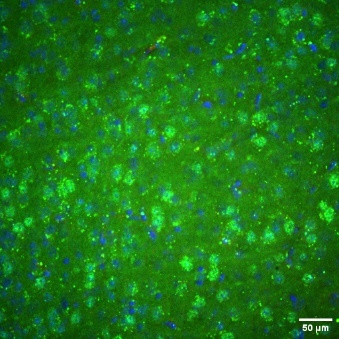** |
| **CA1** | **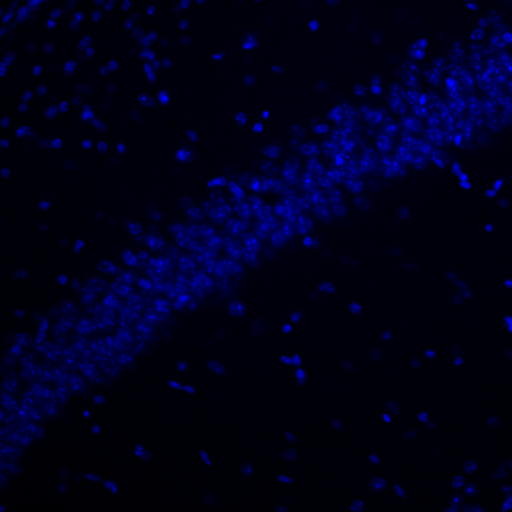** | **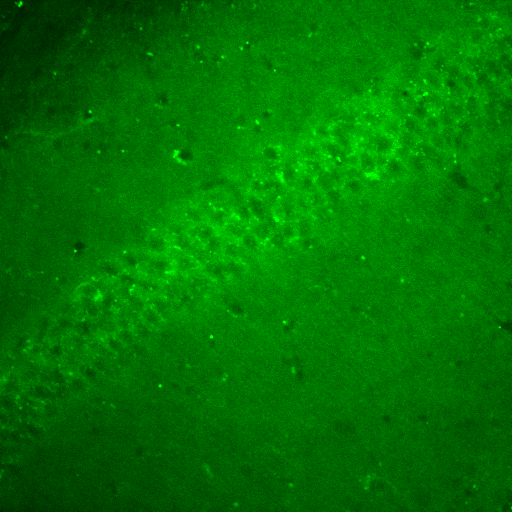** | **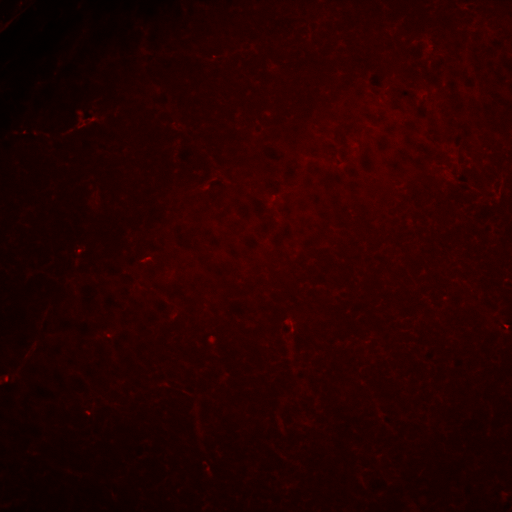** | **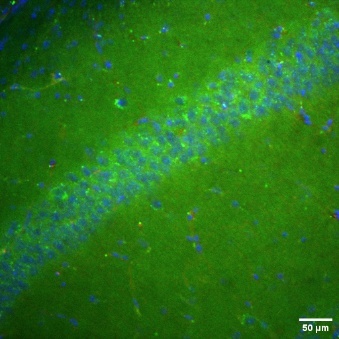** |
| **CA3** | **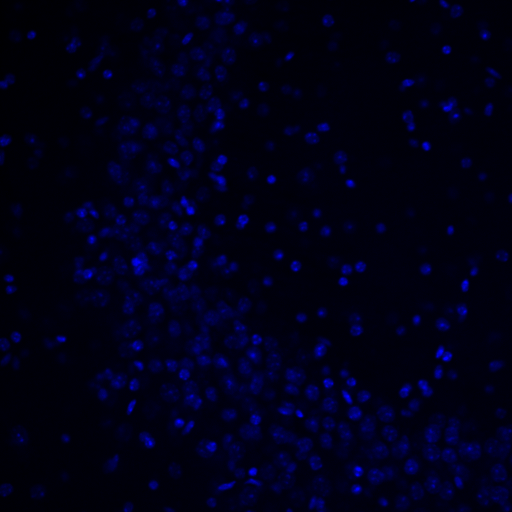** | **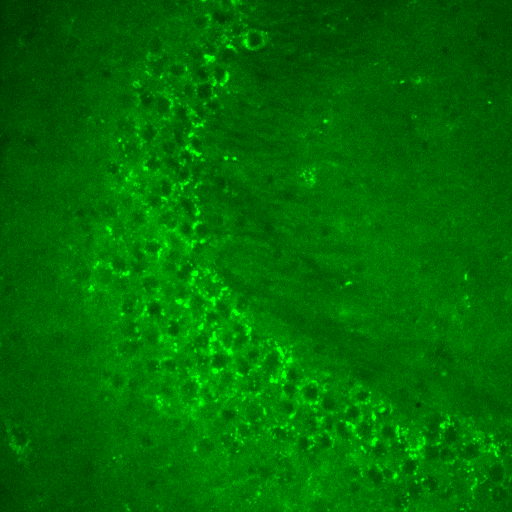** | **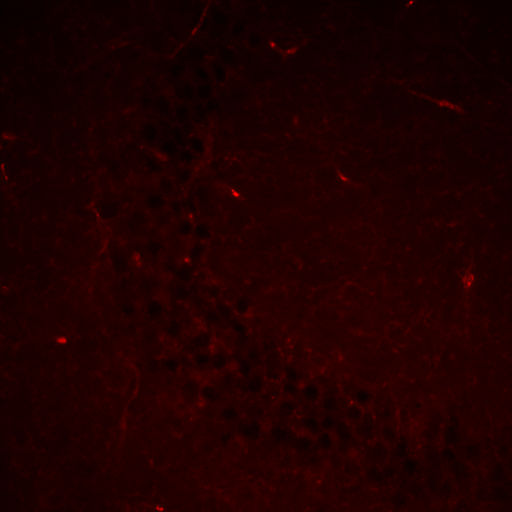** | **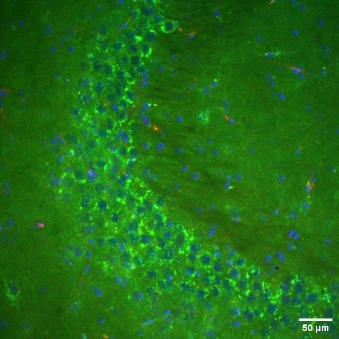** |
| **DG** | **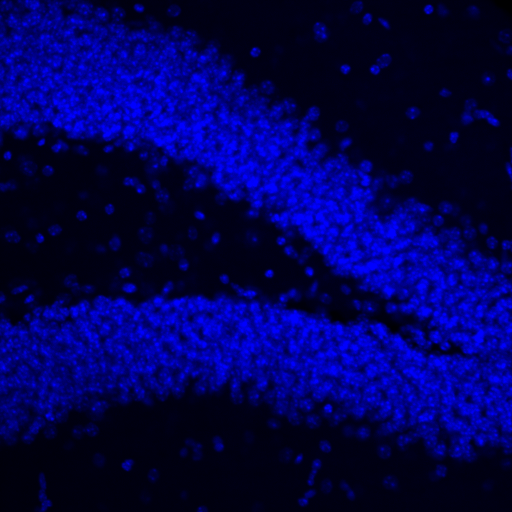** | **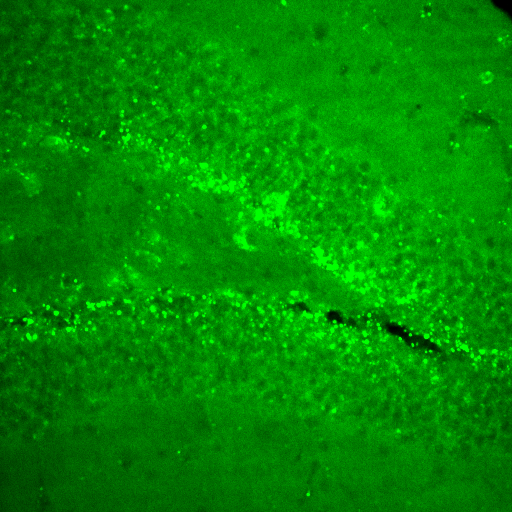** | **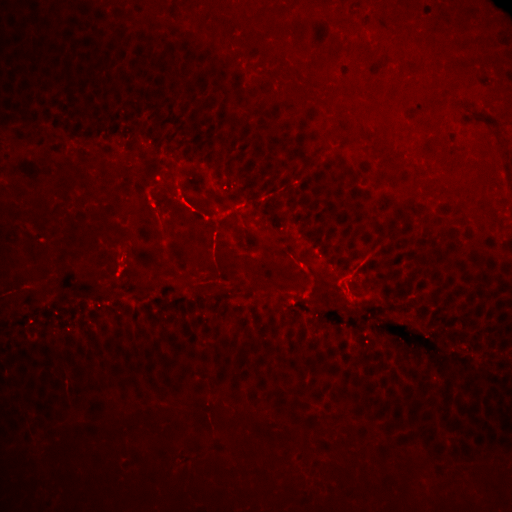** | **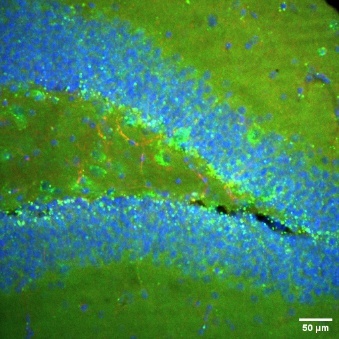** |

**B.**

| **Brain Region** | **Dapi** | **14-3-3ζ** | **CaMKIIα** | **Merge** |
| --- | --- | --- | --- | --- |
| **mPFC** | **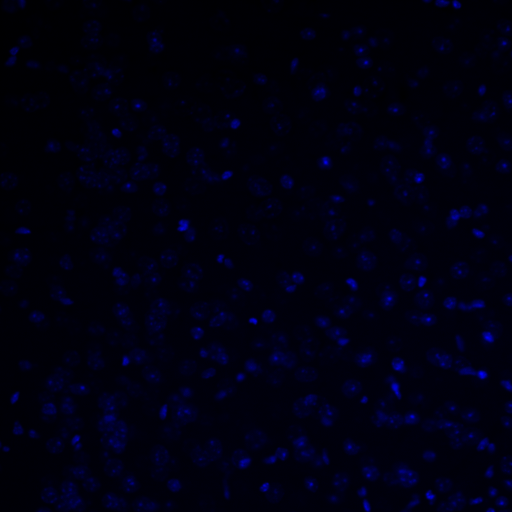** | **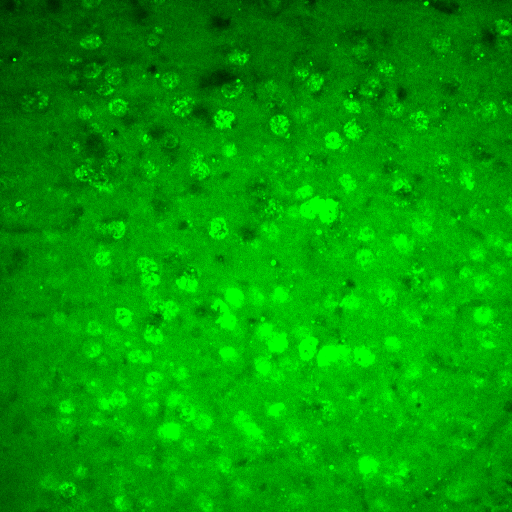** | **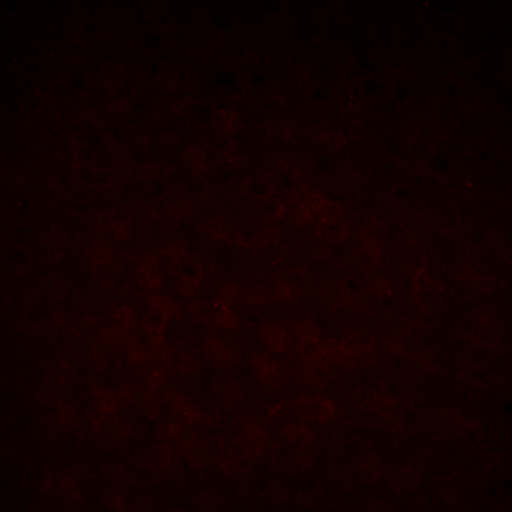** | **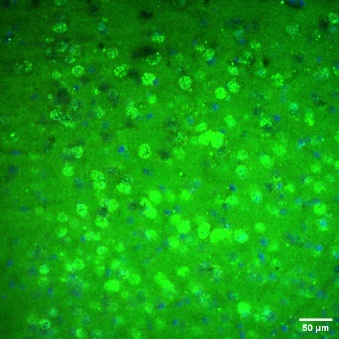** |
| **CA1** | **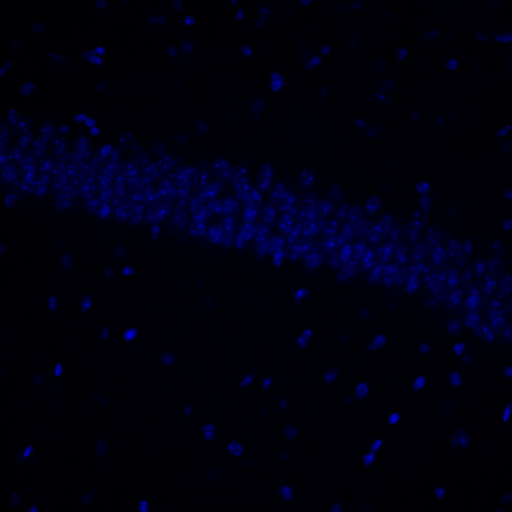** | **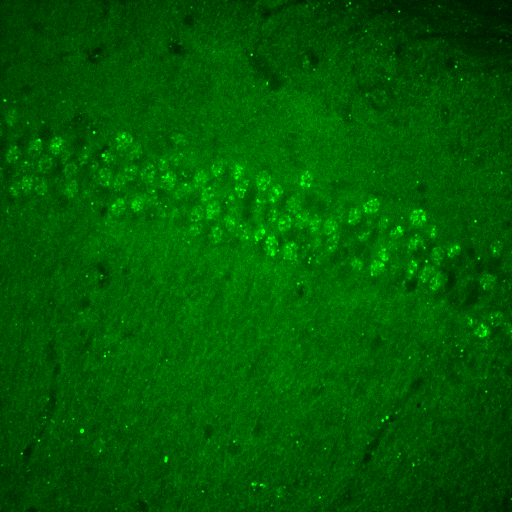** | **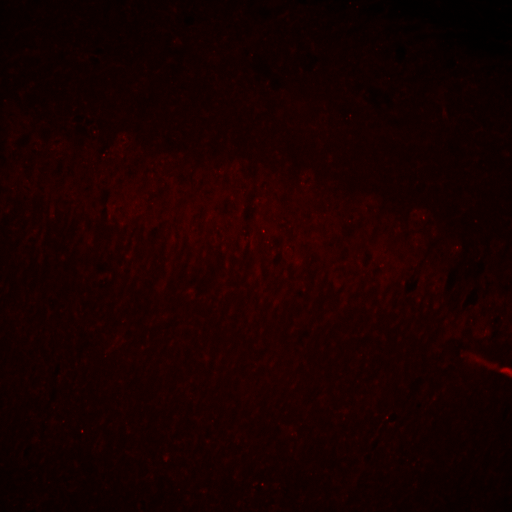** | **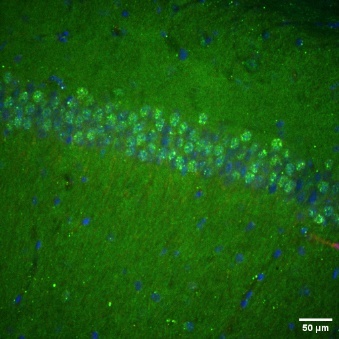** |
| **CA3** | **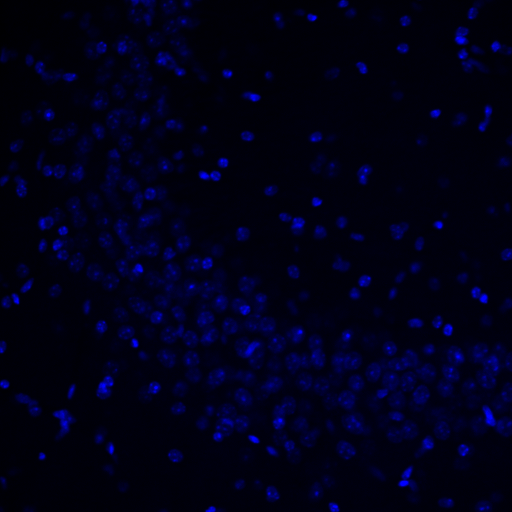** | **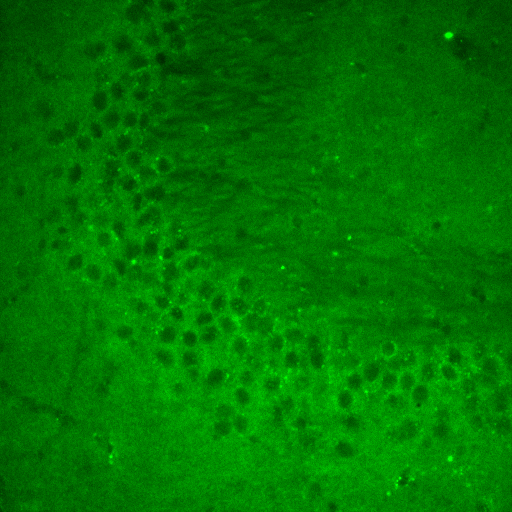** | **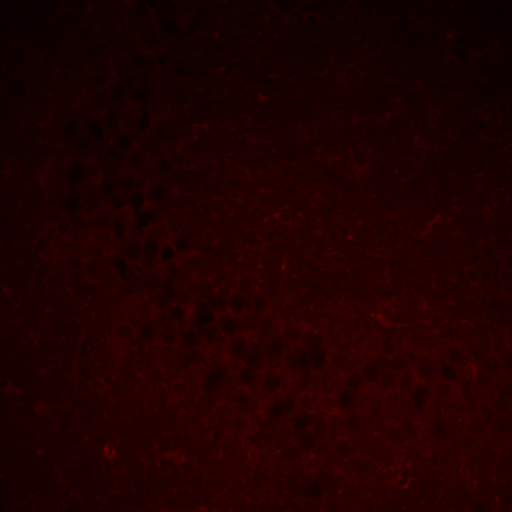** | **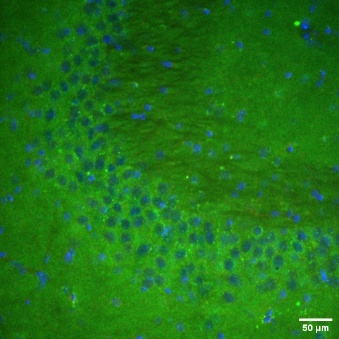** |
| **DG** | **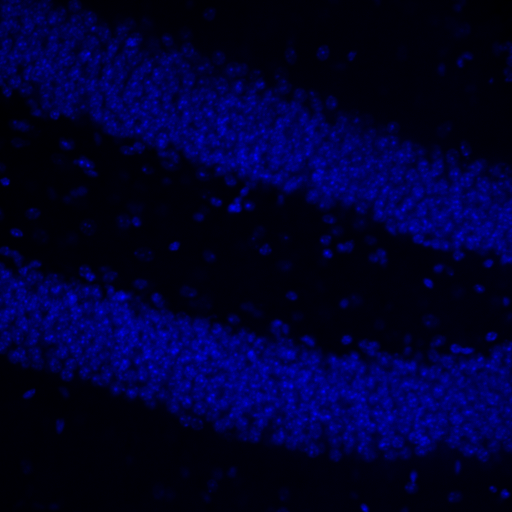** | **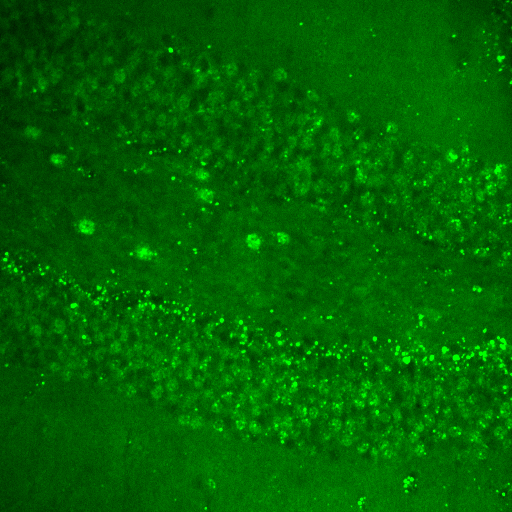** | **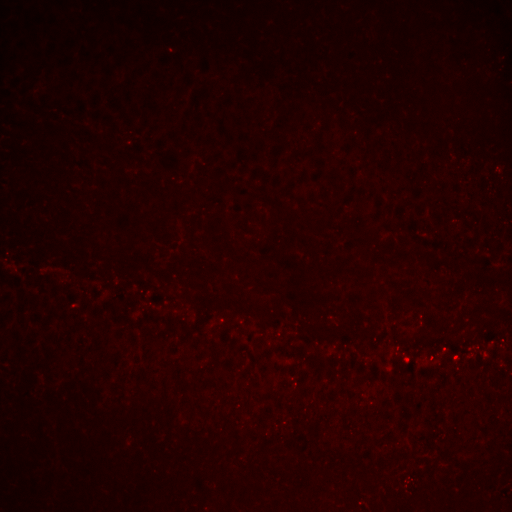** | **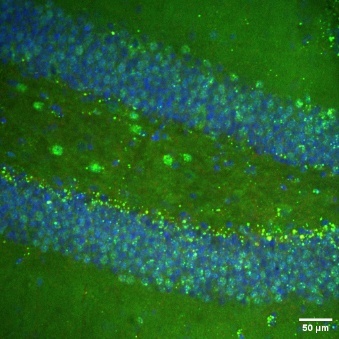** |

**C.**

| **Brain Region** | **Dapi** | **14-3-3ε** | **CaMKIIα** | **Merge** |
| --- | --- | --- | --- | --- |
| **mPFC** | **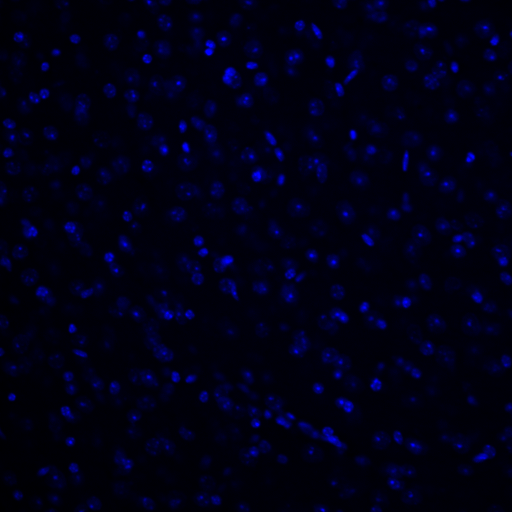** | **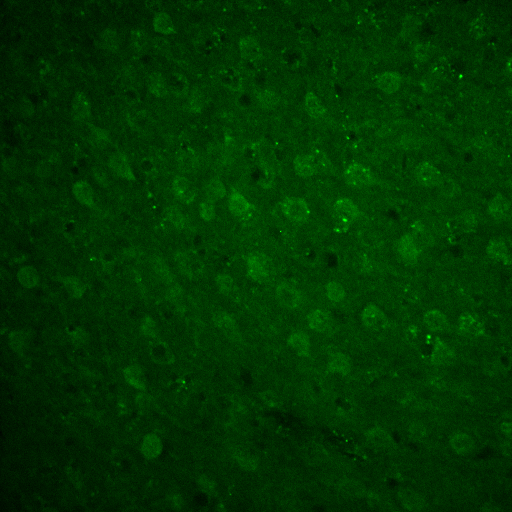** | **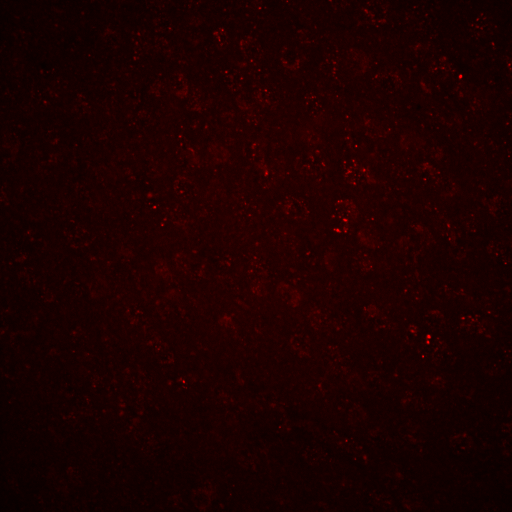** | **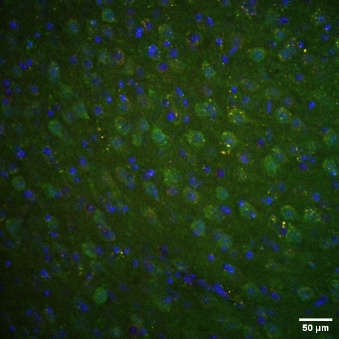** |
| **CA1** | **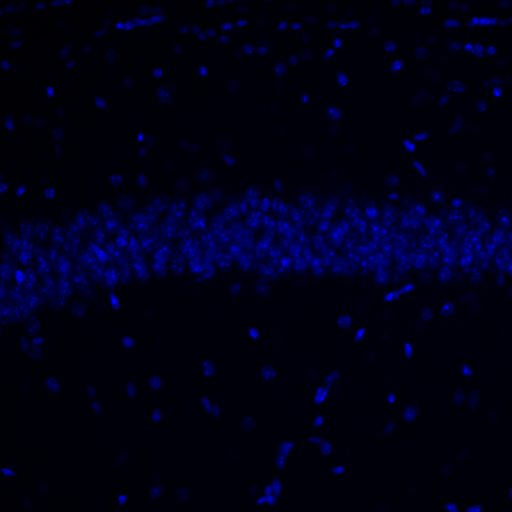** | **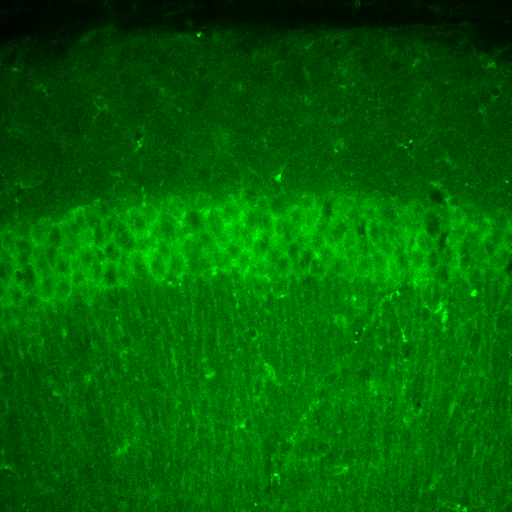** | **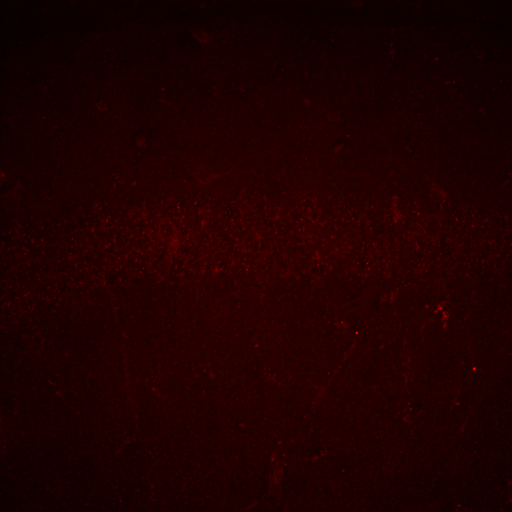** | **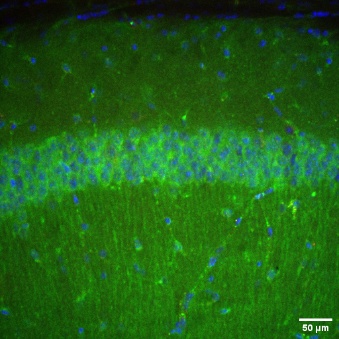** |
| **CA3** | **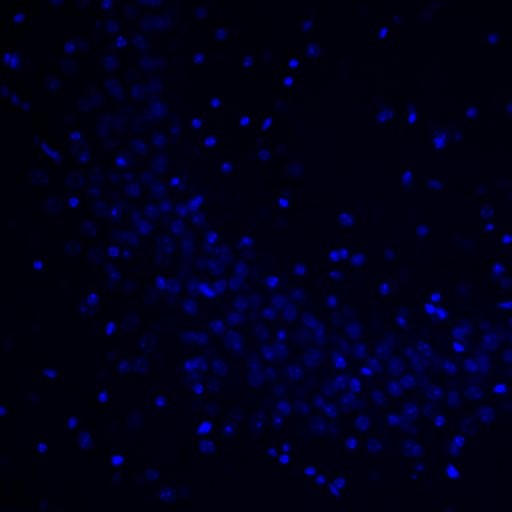** | **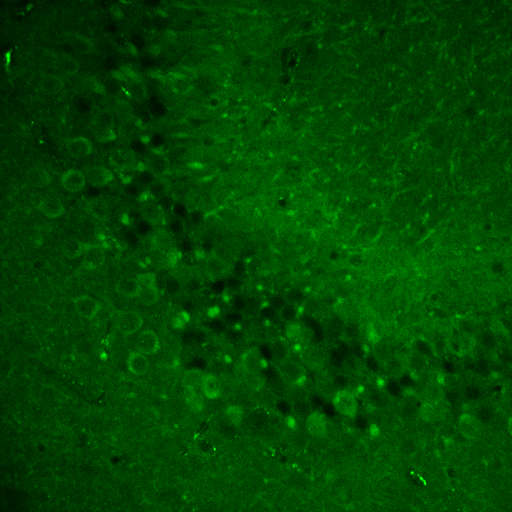** | **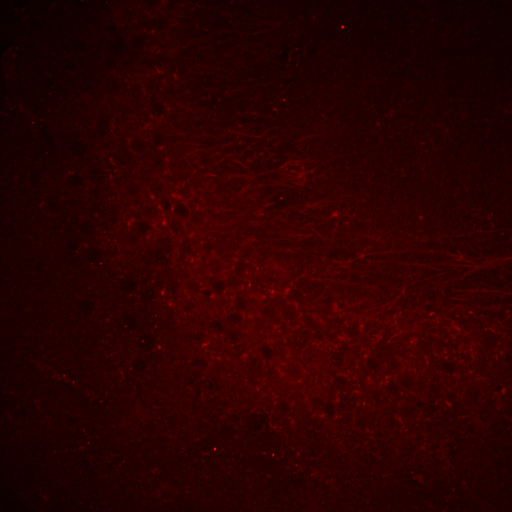** | **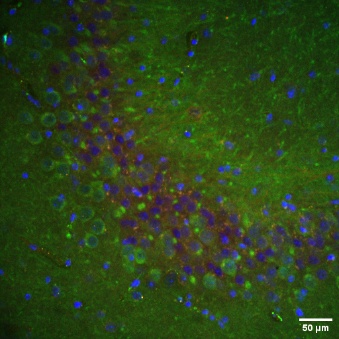** |
| **DG** | **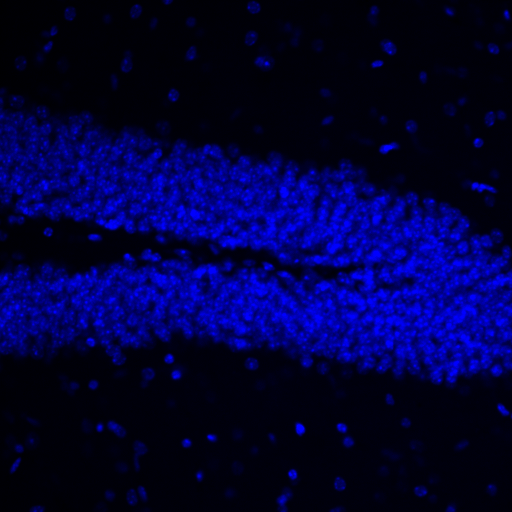** | **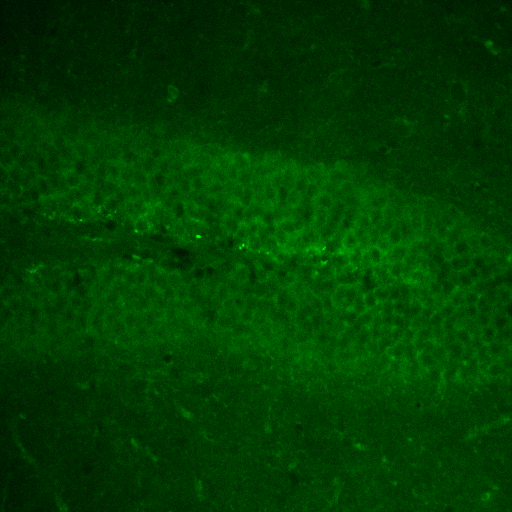** | **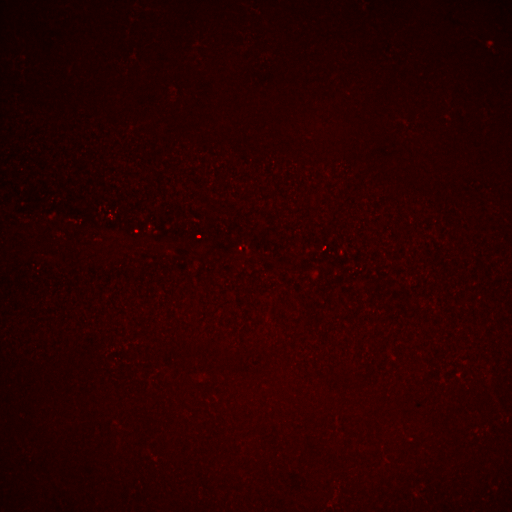** | **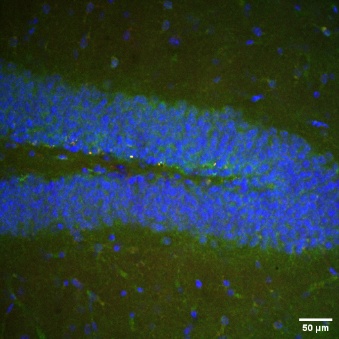** |

**D.**

| **Brain Region** | **Dapi** | **14-3-3ε** | **CaMKIIα** | **Merge** |
| --- | --- | --- | --- | --- |
| **mPFC** | **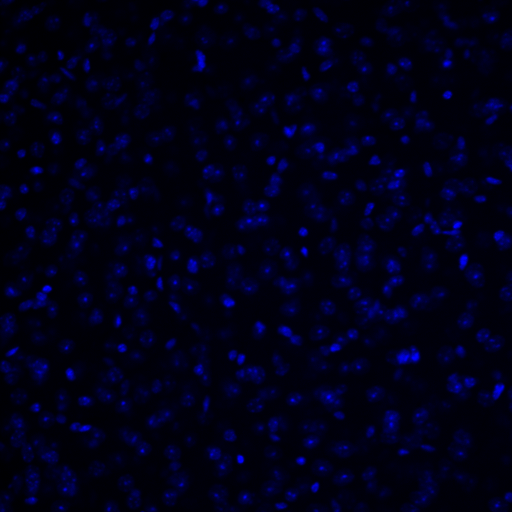** | **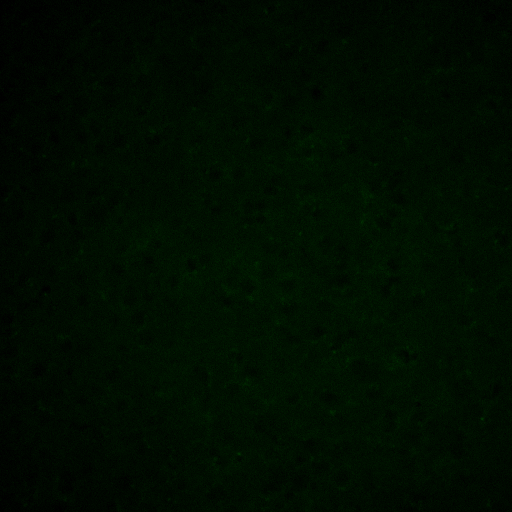** | **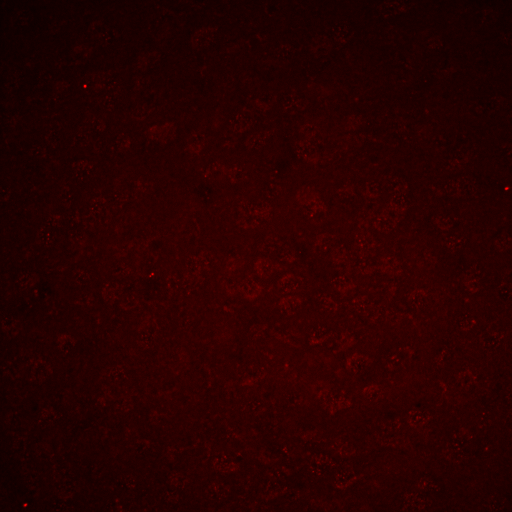** | **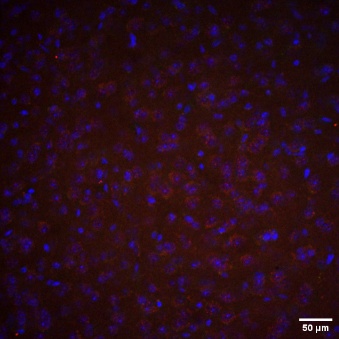** |
| **CA1** | **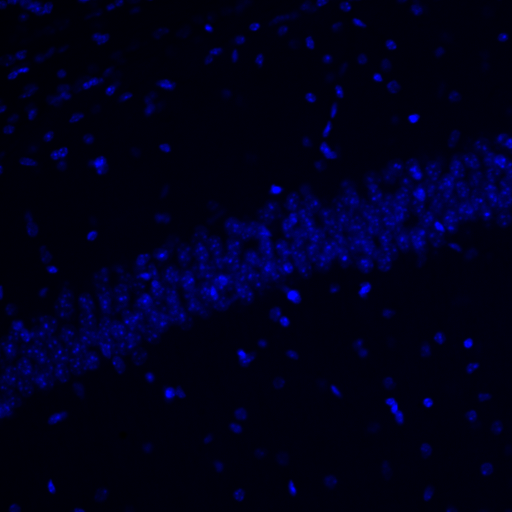** | **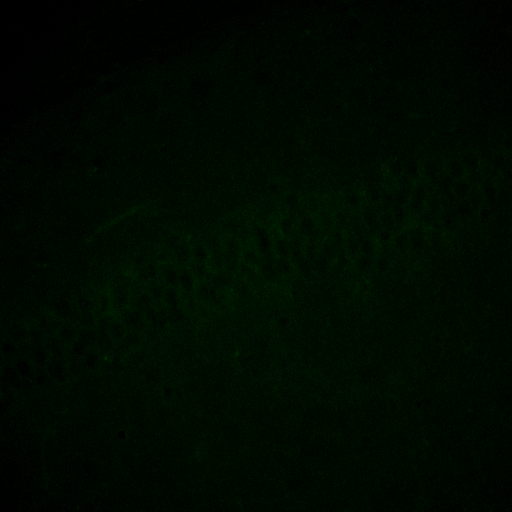** | **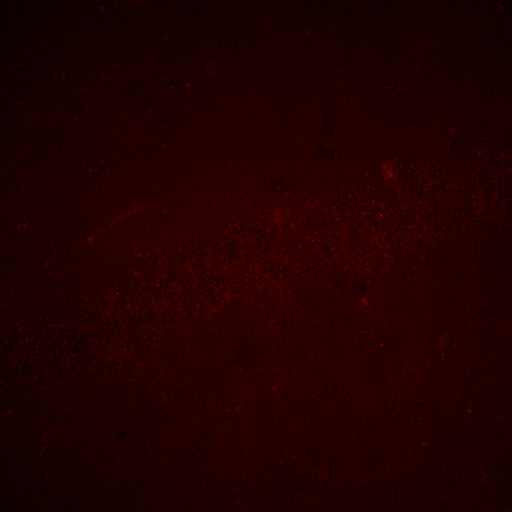** | **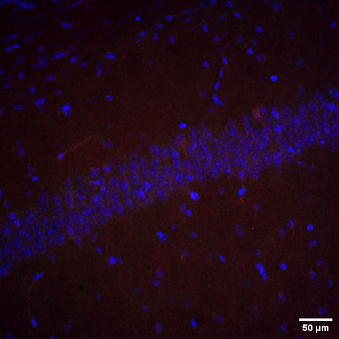** |
| **CA3** | **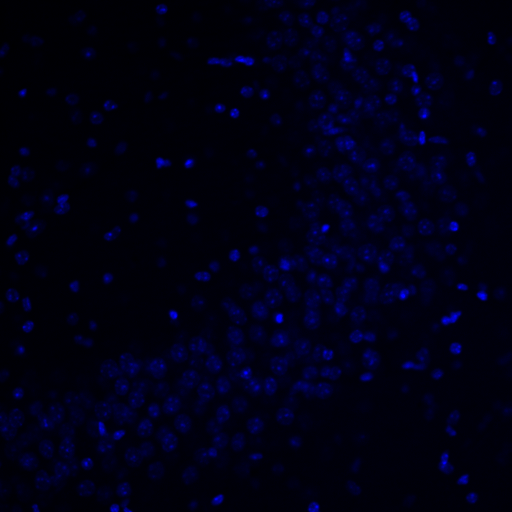** | **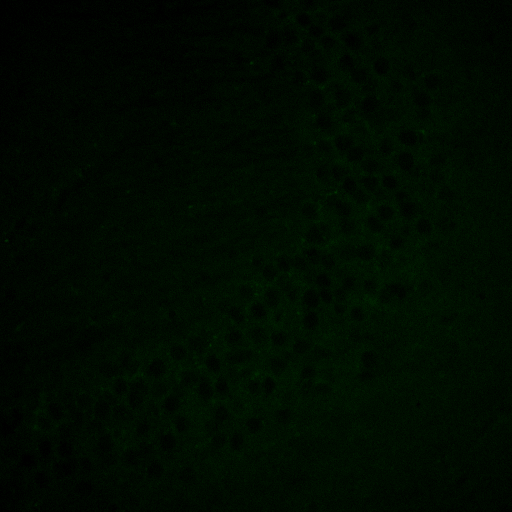** | **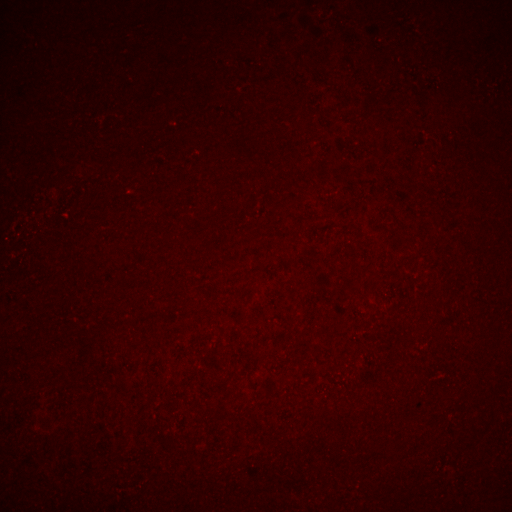** | **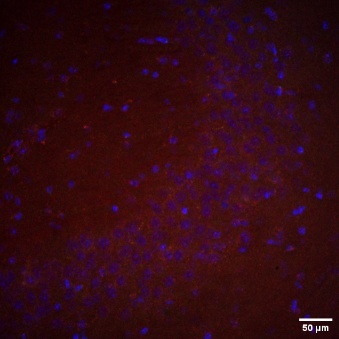** |
| **DG** | **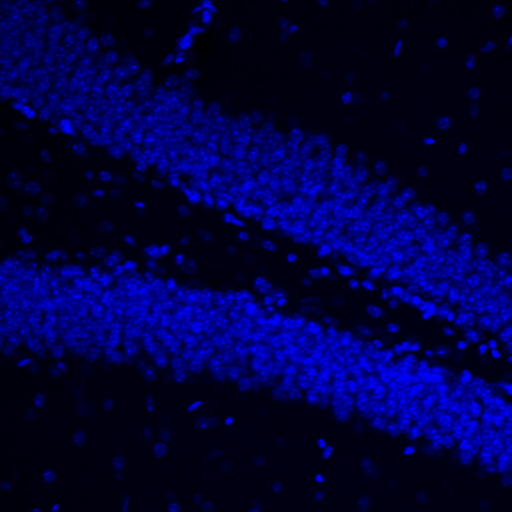** | **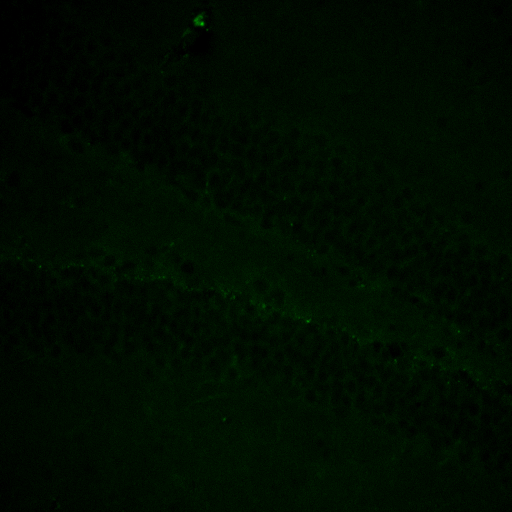** | **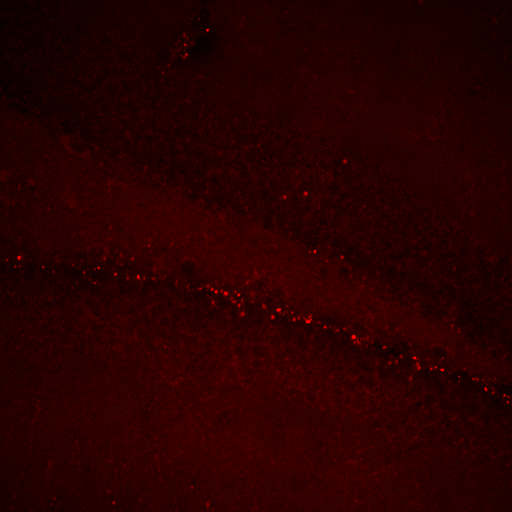** | **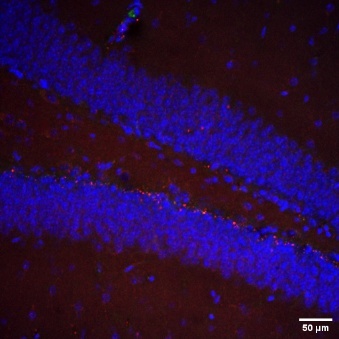** |

**S1 Fig. Split and merged representative IHC images.** Representative fluorescence images used in Fig. 3 are shown split by color channel and merged (scale bar = 50µm). Brain slices from a dFlC (A) and a CKO (B) animal were co-labeled with dapi (blue), CaMKIIα (red), and 14-3-3ζ (green). Brain slices from a dFlC (C) and a CKO (D) animal were co-labeled with dapi (blue), CaMKIIα (red), and 14-3-3ε (green).
