# Supplementary material for: Behavioral analyses of a forebrain glutamatergic neuron specific Ywhae conditional knockout mouse model
Source: PLoS One. 2025 Nov 11;20(11):e0335427. doi: 10.1371/journal.pone.0335427 (PMC12604760; doi:10.1371/journal.pone.0335427)
Supplement: S2 Fig — Representative fluorescence images used in Fig. 3 are shown split by color channel and merged (scale bar = 50µm). Brain slices from a dFlC (A) and a CKO (B) animal were co-labeled with dapi (blue), CaMKIIα (red), and 14-3-3ζ (green). Brain slices from a dFlC (C) and a CKO (D) animal were co-labeled with dapi (blue), CaMKIIα (red), and 14-3-3ε (green). (DOCX) [file pone.0335427.s004.docx]

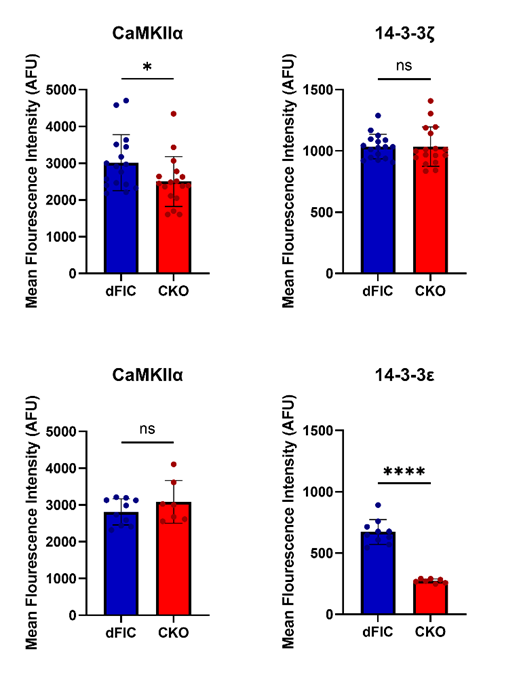

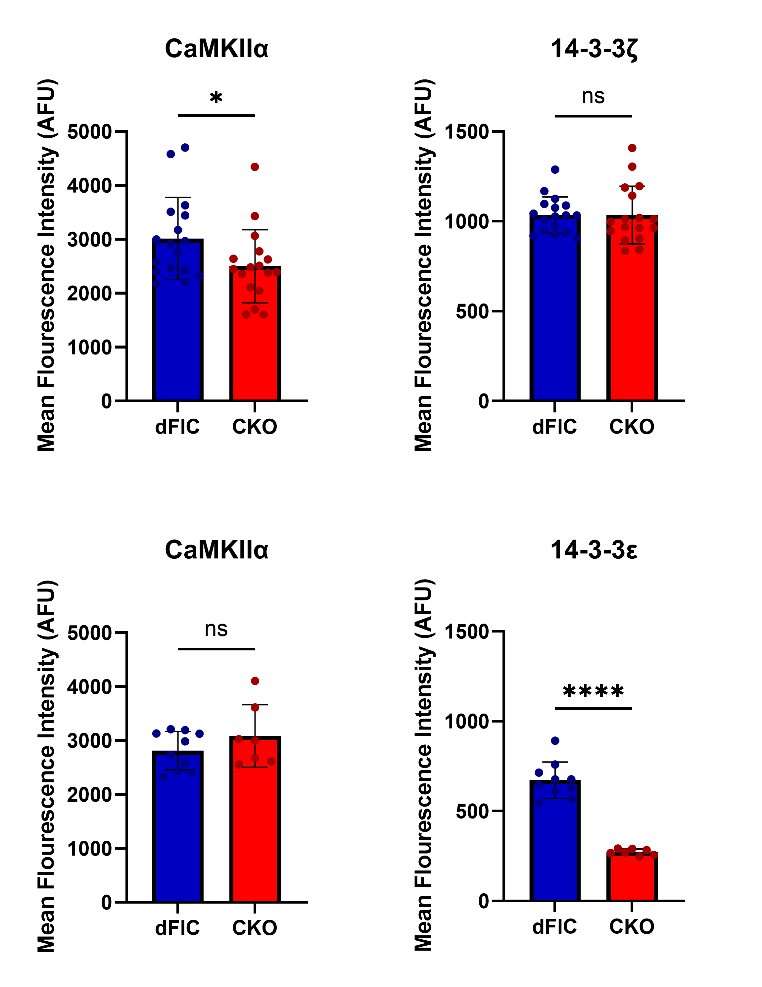


**A. B. C. D.**

**S2 Fig. Analysis of mean fluorescence intensity.** Means are plotted in units of Arbitrary Fluorescence Units (AFU). Brain slices from dFlC and CKO mice underwent IHC for co-labeling of either CaMKIIα / 14-3-3ζ (dFlC N=17, CKO N=17) (A, B) or CaMKIIα / 14-3-3ε (dFlC N=10, CKO N=7) (C, D). 20x fluorescence images from the CA1, CA3, DG, and mPFC were taken and the mean fluorescence intensity was measured for each image. While there was a slight statistically significant difference in CaMKIIα expression between dFlC and CKO mice, this difference is likely non-meaningful as suggested by a p-value of 0.046 (A). Expression of 14-3-3ζ was consistent across dFlC and CKO mice (B). CaMKIIα expression was consistent across dFlC and CKO mice (C), while there was a significant reduction in 14-3-3ε expression in CKO mice compared to dFlC mice (D). These results are consistent with the isoform specific conditional knockout of 14-3-3ε in our CKO model.
