# Supplementary material for: Behavioral analyses of a forebrain glutamatergic neuron specific Ywhae conditional knockout mouse model
Source: PLoS One. 2025 Nov 11;20(11):e0335427. doi: 10.1371/journal.pone.0335427 (PMC12604760; doi:10.1371/journal.pone.0335427)
Supplement: S3 Fig — Distance traveled during three time-bins, consisting of 10-minute periods, is plotted to determine if animals acclimate to the test arena and locomote differently as time goes on. There is a significant difference in distance traveled across the different time-bins, indicating that animals locomote less as time goes on. However, there is neither a sex nor genotype difference. (Three-way ANOVA; Genotype: F(1, 62)=0.6717, p = 0.4156; Sex: F(1, 62)=1.498, p = 0.2255; Time Bin: F(1.820, 112.8)=7870, Geisser-Greenhouse’s epsilon = 0.9099, p < 0.0001****; Time Bin x Genotype: F(2, 124)=0.5571, p = 0.5743; Time Bin x Sex: F(2, 124)=0.7497, p = 0.4746; Genotype x Sex: F(1, 62)=0.005178, p = 0.9429; Chamber x Genotype x Sex: F(2, 124)=0.3429, p = 0.7104) (CKO-male: N = 19, CKO-female: N = 16, dFlC-male: N = 14, dFlC-female: N = 17). (DOCX) [file pone.0335427.s005.docx]

**
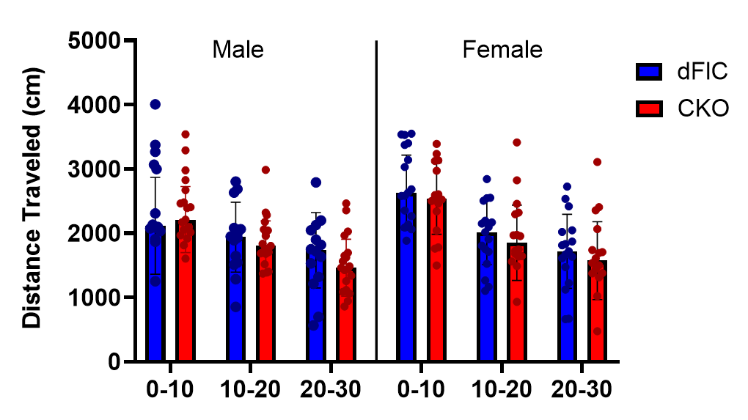
**

**S3 Fig. Acclimation in the Open Field Test.** Distance traveled during three time-bins, consisting of 10-minute periods, is plotted to determine if animals acclimate to the test arena and locomote differently as time goes on. There is a significant difference in distance traveled across the different time-bins, indicating that animals locomote less as time goes on. However, there is neither a sex nor genotype difference. (Three-way ANOVA; Genotype: F(1, 62)=0.6717, p=0.4156; Sex: F(1, 62)=1.498, p=0.2255; Time Bin: F(1.820, 112.8)=7870, Geisser-Greenhouse’s epsilon=0.9099, p<0.0001****; Time Bin x Genotype: F(2, 124)=0.5571, p=0.5743; Time Bin x Sex: F(2, 124)=0.7497, p=0.4746; Genotype x Sex: F(1, 62)=0.005178, p=0.9429; Chamber x Genotype x Sex: F(2, 124)=0.3429, p=0.7104) (CKO-male: N=19, CKO-female: N=16, dFlC-male: N=14, dFlC-female: N=17).
