# Supplementary material for: Behavioral analyses of a forebrain glutamatergic neuron specific Ywhae conditional knockout mouse model
Source: PLoS One. 2025 Nov 11;20(11):e0335427. doi: 10.1371/journal.pone.0335427 (PMC12604760; doi:10.1371/journal.pone.0335427)
Supplement: S4 Fig — There was a significant main effect of sex in vertical rearing events, with males exhibiting a higher number of vertical counts than females. Further, there was a main effect of age, with mice 3–4 months of age exhibiting a higher number of vertical counts than mice 2–3 months of age (CKO-male: N = 19, CKO-female: N = 16, dFlC-male: N = 14, dFlC-female: N = 19). (DOCX) [file pone.0335427.s006.docx]

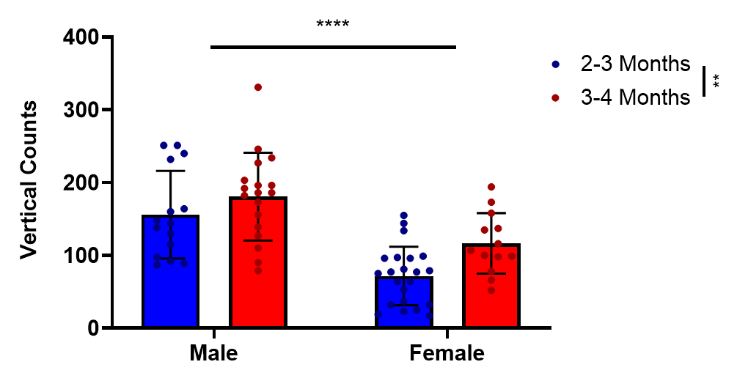


**S4 Fig.** **Effect of age on rearing activity.** There was a significant main effect of sex in vertical rearing events, with males exhibiting a higher number of vertical counts than females. Further, there was a main effect of age, with mice 3-4 months of age exhibiting a higher number of vertical counts than mice 2-3 months of age (CKO-male: N=19, CKO-female: N=16, dFlC-male: N=14, dFlC-female: N=19).
