# Supplementary material for: Behavioral analyses of a forebrain glutamatergic neuron specific Ywhae conditional knockout mouse model
Source: PLoS One. 2025 Nov 11;20(11):e0335427. doi: 10.1371/journal.pone.0335427 (PMC12604760; doi:10.1371/journal.pone.0335427)
Supplement: S5 Fig — While no genotype difference was observed in the ratio of time spent at the Edge over the time spent in the Center of the arena in the 30-minute OFT, there was a significant sex difference, indicating that females spent more time at the edge of the arena (ratio calculated as [(Time spent at the edge)/ (Time spent in the center)]) (Two-way ANOVA; Genotype: F(1, 38)=3.117, p = 0.0855; Sex: F(1, 38)=4.964, p = 0.0319*; Interaction: F(1, 38)=0.4455, p = 0.5085) (CKO-male: N = 9, CKO-female: N = 11, dFlC-male: N = 8, dFlC-female: N = 14). (DOCX) [file pone.0335427.s007.docx]

**
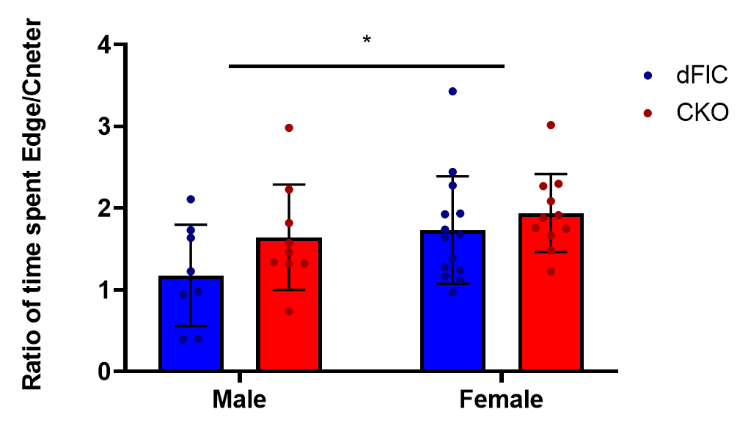
**

**S5 Fig.** **Evaluation of thigmotaxis.** While no genotype difference was observed in the ratio of time spent at the Edge over the time spent in the Center of the arena in the 30-minute OFT, there was a significant sex difference, indicating that females spent more time at the edge of the arena (ratio calculated as [(Time spent at the edge) / (Time spent in the center)]) (Two-way ANOVA; Genotype: F(1, 38)=3.117, p=0.0855; Sex: F(1, 38)=4.964, p=0.0319*; Interaction: F(1, 38)=0.4455, p=0.5085) (CKO-male: N=9, CKO-female: N=11, dFlC-male: N=8, dFlC-female: N=14).
