# Supplementary material for: Behavioral analyses of a forebrain glutamatergic neuron specific Ywhae conditional knockout mouse model
Source: PLoS One. 2025 Nov 11;20(11):e0335427. doi: 10.1371/journal.pone.0335427 (PMC12604760; doi:10.1371/journal.pone.0335427)
Supplement: S6 Fig — Means are plotted along with 95% confidence intervals. There is a crossover interaction between the Test Session and Sex. (DOCX) [file pone.0335427.s008.docx]

**
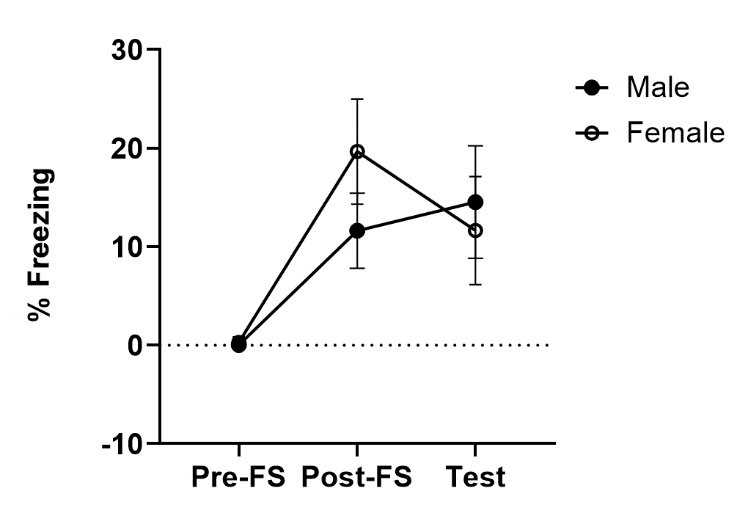
**

**S6 Fig. An interaction plot investigating the significant Test Session x Sex interaction in the Fear Conditioning test.** Means are plotted along with 95% confidence intervals. There is a crossover interaction between the Test Session and Sex.
