# Supplementary material for: Behavioral analyses of a forebrain glutamatergic neuron specific Ywhae conditional knockout mouse model
Source: PLoS One. 2025 Nov 11;20(11):e0335427. doi: 10.1371/journal.pone.0335427 (PMC12604760; doi:10.1371/journal.pone.0335427)
Supplement: S7 Fig — Means are plotted along with 95% confidence intervals. There is a crossover interaction between the Camber and Sex. (DOCX) [file pone.0335427.s009.docx]

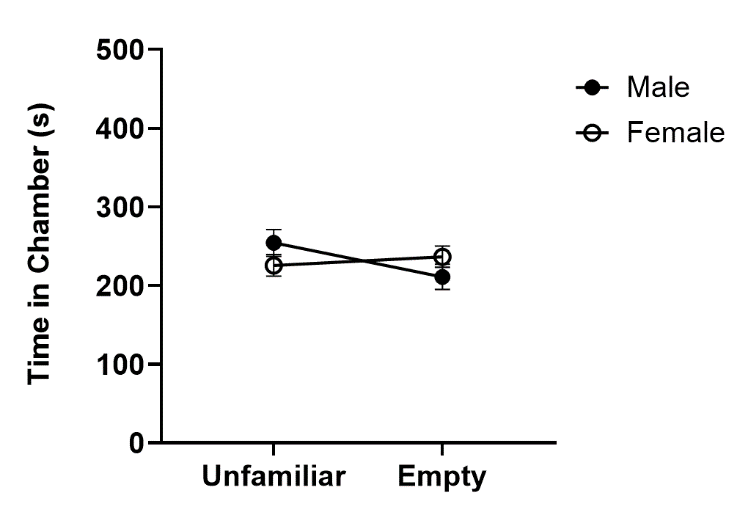


**S7 Fig. An interaction plot investigating the significant Chamber x Sex interaction in the Sociability Test.** Means are plotted along with 95% confidence intervals. There is a crossover interaction between the Camber and Sex.
