# Supplementary material for: Behavioral analyses of a forebrain glutamatergic neuron specific Ywhae conditional knockout mouse model
Source: PLoS One. 2025 Nov 11;20(11):e0335427. doi: 10.1371/journal.pone.0335427 (PMC12604760; doi:10.1371/journal.pone.0335427)
Supplement: S8 Fig — Means are plotted along with 95% confidence intervals. There is a crossover interaction between the Genotype and Age for mice 2–3 months of age (A) but not mice 3–4 months of age (B). (DOCX) [file pone.0335427.s010.docx]

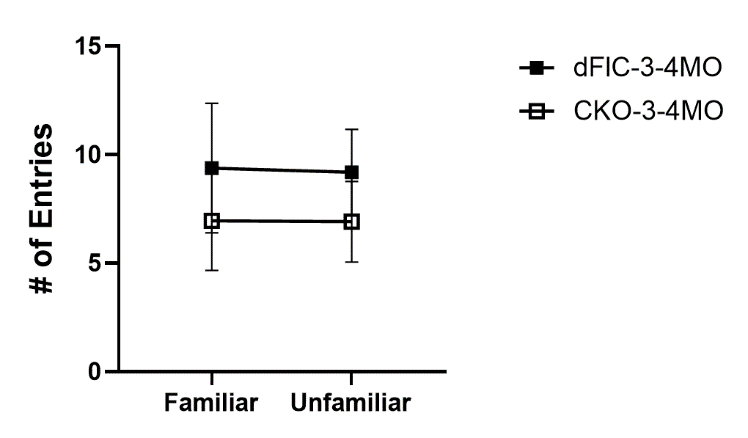

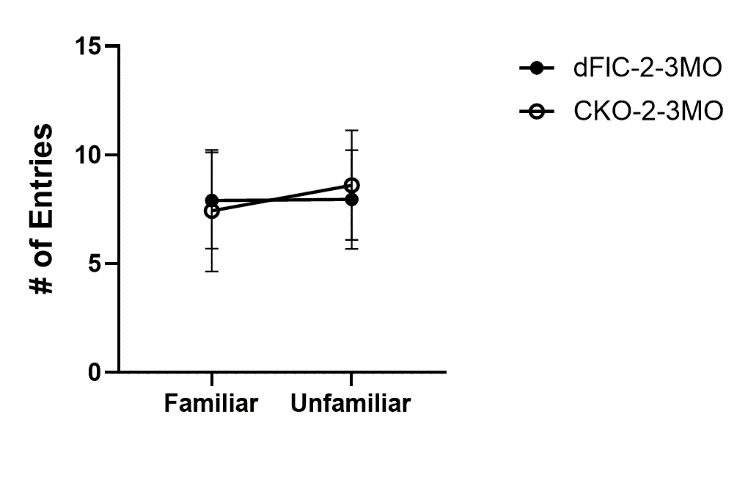


**A. B.**

**S8 Fig. Interaction plots investigating the significant Genotype x Age interaction in the Social Novelty Test.** Means are plotted along with 95% confidence intervals. There is a crossover interaction between the Genotype and Age for mice 2-3 months of age (A) but not mice 3-4 months of age (B).
