# Supplementary material for: Behavioral analyses of a forebrain glutamatergic neuron specific Ywhae conditional knockout mouse model
Source: PLoS One. 2025 Nov 11;20(11):e0335427. doi: 10.1371/journal.pone.0335427 (PMC12604760; doi:10.1371/journal.pone.0335427)
Supplement: S9 Fig — Because dFlC mice did not exhibit normal social behavior when compared to CKO mice, a new group of dFlC mice (N = 12) and a group of wildtype C57BL/6J mice (N = 13) were put through the sociability and social novelty tests to evaluate their social behavior. dFlC and B6 groups were age and sex matched. In the sociability test, B6 mice showed preference for an unfamiliar mouse over an empty cup, demonstrating normal sociability behavior. In alignment with the main body of this study, dFlC mice did not show a preference for sociability (Time, Two-way ANOVA; Genotype: F(1, 20)=0.5279, p = 0.2372; Chamber: F(1, 20)=7.990, p = 0.0104*; Chamber x Genotype: F(1, 20)=6.886, p = 0.0163*; Subject: F(20, 20)=0.1665, p > 0.9999) (A). While there was no main effects of Chamber or Genotype on entries performed in the sociability test, there was a highly significant variability amongst subjects (Time, Two-way ANOVA; Genotype: F(1, 20)=0.02387, p = 0.8788; Chamber: F(1, 20)=3.800, p = 0.0654; Chamber x Genotype: F(1, 20)=8.140, p = 0.0098**; Subject: F(20, 20)=4.364, p = 0.0009***) (B). In the social novelty test, B6 mice showed preference for an unfamiliar mouse over a familiar mouse, demonstrating normal social novelty behavior. However, in discordance with the main body of this study, dFlC mice also showed social novelty preference (Time, Two-way ANOVA; Genotype: F(1, 20)=0.6154, p = 0.4420; Chamber: F(1, 20)=12.57, p = 0.0020**; Chamber x Genotype: F(1, 20)=0.7032, p = 0.4116; Subject: F(20, 20)=0.1635, p > 0.9999) (C). While there was no main effects of Chamber or Genotype on entries performed in the social novelty test, there was a highly significant variability amongst subjects (Time, Two-way ANOVA; Genotype: F(1, 20)=0.8066, p = 0.3798; Chamber: F(1, 20)=2.762, p = 0.1121; Chamber x Genotype: F(1, 20)=0.7469, p = 0.3977; Subject: F(20, 20)=3.647, p = 0.0028**) (D). We then compared the social behavior results from dFlC mice in our main analysis to the new group of dFlC mice [file pone.0335427.s011.docx]

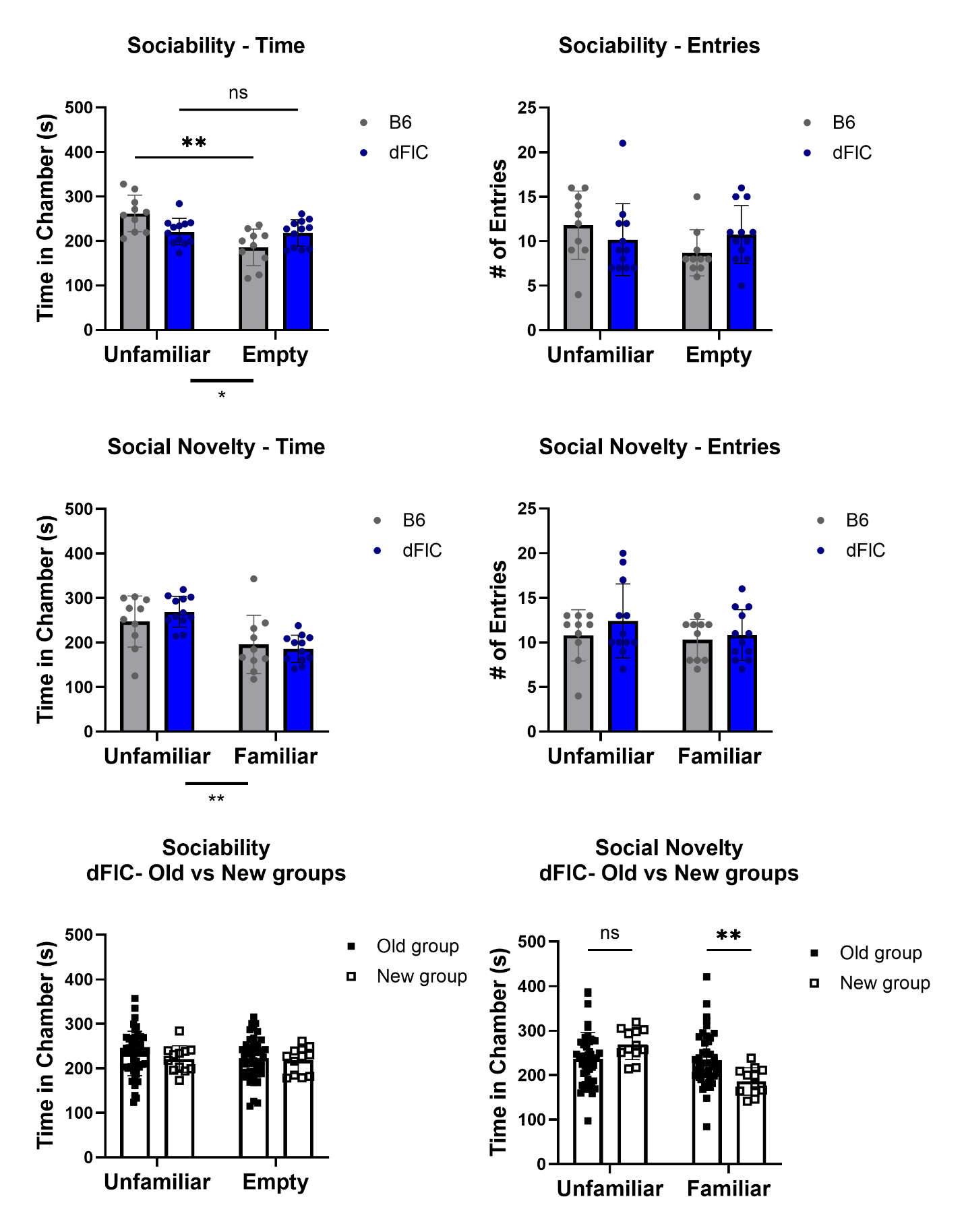


**A. B.**

**C. D.**

**E. F.**

**S9 Fig. Comparing dFlC social behavior to B6 controls.** Because dFlC mice did not exhibit normal social behavior when compared to CKO mice, a new group of dFlC mice (N=12) and a group of wildtype C57BL/6J mice (N=13) were put through the sociability and social novelty tests to evaluate their social behavior. dFlC and B6 groups were age and sex matched. In the sociability test, B6 mice showed preference for an unfamiliar mouse over an empty cup, demonstrating normal sociability behavior. In alignment with the main body of this study, dFlC mice did not show a preference for sociability (Time, Two-way ANOVA; Genotype: F(1, 20)=0.5279, p=0.2372; Chamber: F(1, 20)=7.990, p=0.0104*; Chamber x Genotype: F(1, 20)=6.886, p=0.0163*; Subject: F(20, 20)=0.1665, p>0.9999) (A). While there was no main effects of Chamber or Genotype on entries performed in the sociability test, there was a highly significant variability amongst subjects (Time, Two-way ANOVA; Genotype: F(1, 20)=0.02387, p=0.8788; Chamber: F(1, 20)=3.800, p=0.0654; Chamber x Genotype: F(1, 20)=8.140, p=0.0098**; Subject: F(20, 20)=4.364, p=0.0009***) (B). In the social novelty test, B6 mice showed preference for an unfamiliar mouse over a familiar mouse, demonstrating normal social novelty behavior. However, in discordance with the main body of this study, dFlC mice also showed social novelty preference (Time, Two-way ANOVA; Genotype: F(1, 20)=0.6154, p=0.4420; Chamber: F(1, 20)=12.57, p=0.0020**; Chamber x Genotype: F(1, 20)=0.7032, p=0.4116; Subject: F(20, 20)=0.1635, p>0.9999) (C). While there was no main effects of Chamber or Genotype on entries performed in the social novelty test, there was a highly significant variability amongst subjects (Time, Two-way ANOVA; Genotype: F(1, 20)=0.8066, p=0.3798; Chamber: F(1, 20)=2.762, p=0.1121; Chamber x Genotype: F(1, 20)=0.7469, p=0.3977; Subject: F(20, 20)=3.647, p=0.0028**) (D). We then compared the social behavior results from dFlC mice in our main analysis to the new group of dFlC mice used in this experiment. The old and new groups of dFlC mice did not differ in their performance in the sociability test (Time, Two-way ANOVA; New vs Old: F(1, 56)=1.781, p=0.1875; Chamber: F(1, 56)=0.2594, p=0.6125; Chamber x New vs Old: F(1, 56)=0.08555, p=0.7710; Subject: F(56, 56)=0.2536, p>0.9999) (E). In the social novelty test, there was no main effect of New vs Old group. While the new group of dFlC mice spent a significantly shorter amount of time with a familiar mouse in the social novelty test, they did not differ from the old group of dFlC mice in the amount of time spent with an unfamiliar mouse. This may suggest that the difference between the groups of dFlC mice is influenced by the sampling of a smaller group in this experiment (Time, Two-way ANOVA; New vs Old: F(1, 57)=1.318, p=0.2558; Chamber: F(1, 57)=6.893, p=0.0111*; Chamber x New vs Old: F(1, 57)=5.864, p=0.0187*; Subject: F(57, 57)=0.1448, p>0.9999) (F).
