# Supplementary material for: Behavioral analyses of a forebrain glutamatergic neuron specific Ywhae conditional knockout mouse model
Source: PLoS One. 2025 Nov 11;20(11):e0335427. doi: 10.1371/journal.pone.0335427 (PMC12604760; doi:10.1371/journal.pone.0335427)
Supplement: S1 File — (PDF) [file pone.0335427.s012.pdf]

BODY WEIGHT WEEK 1-8

Week	dFIC										
1	5.3	3.4								5.432		
2	7.1	7.53	7.4	6.2	6.6	7.3				6.599		
3	8.5	8.25	7.65	8	7.625	8.76	9.55	9.39	10.09	7.6	7.3	9.7
4	11.3	11.5	13.97	14.7	12.38	11.88				11.425	11.65	14.36
5	15.9	17.1	18.269	18.02	20.225	17.78	18.2			14.77	15.183	
6	19.52	18.4375	21.45	20.22	20.678	21.24				16.7	18.075	
7	23.3	22.9	23.1	20.4	22.9					17.6	18.7	
8	25.86	25.03	25.09	24.612	25.74	21.49				20.42	21.64	

Week	CKO																	
1	3.6857	3.1	3.594	1.955	4.6668									3.1571	5.7			
2	7.3501	6.2	6.6	5.7	5.4	5.62	7.0857	5.9	6.3125					6.4	7.0571	5.425	7.275	6.5334
3	12.4334	9	7.4	8.6714	8.02	9.8666	8.5452	9.27	9.1	7.625	8.6125	6.925	7.2375	8.7	7.35	10.3334	7.52	8.0857
4	18.3286	14.1	12.6	13.125	11.5625	11.675	10.1	11.025						12.6	10.7125	14.6714	9.54	
5	20.6	18.1	19.9311	20.3	16.9	16.1	15.1625	13.1	13.825					15.6	14.1			
6	22.55	20.82	19.95	19.52	19.42	16.86	16.56							17.28	16.28			
7	23.4	21.5888	21.975	20.6875	19.375									17.3776				
8	25.4222	25.8854	24.18											18.9436				

BODY WEIGHT FIRST-LAST BEHAVIOR

	Male-dFIC													
**First Session**	35.77	47.09	30.41	30.06	39.68	31.86	30.14	29.19	25.1	25.3	30.2	30.3	30	29.2
**Last Session**	35.25	49.65	33.01	31.61	44.54	34.13	31.49	30.95	25.4	25.9	31.4	31.8	31.3	29.9

	Male-CKO													
**First Session**	28.96	23.67	24.88	30.68	30.26	42.16	28.83	29.4	27.6	28.2	33	30.9		
**Last Session**	30.76	29.15	27.52	31.94	31.02	46.98	29.73	30.8	29.7	28.9	34.2	31.6		

	Female-dFIC													
**First Session**	36.44	35.71	20.36	20.16	18.73	20.7	20.3	19.2	23.2	22.9	21.8	18.6	20.6	
**Last Session**	40.8	35.31	21.89	21.88	21.73	21.1	21.2	19.7	25	24.5	22	19.8	20.9	

	Female-CKO													
**First Session**	34.2	22.46	21.7	22.64	21.2	20.7	19.5	24.2	26.3	24.2	20.7	23		
**Last Session**	42.72	25.41	23.22	23.81	22.4	21.9	20.8	26.4	25.5	24.5	22.1	25.1		

OPEN FIELD TEST

Group 1					
Animal ID #			Distance	Vertical counts	
735	dFIC	Male	7655.845	221	
738	dFIC	Male	6170.232	140	X removed for being over p150 on behavioral start date
840	dFIC	Male	6180.895	173	
841	dFIC	Male	5689.902	192	
845	dFIC	Male	7366.628	110	
824	dFIC	Male	5945.976	127	
875	dFIC	Male	5952.577	232	
876	dFIC	Male	6047.34	97	
842	dFIC	Female	8318.553	173	
843	dFIC	Female	7514.898	107	
850	dFIC	Female	5760.346	66	
881	dFIC	Female	5491.465	32	
888	dFIC	Female	5813.096	144	
891 (renamed 815)	CKO	Male	4753.303	139	
856	CKO	Male	7884.698	251	
883	CKO	Male	5855.565	144	
886	CKO	Male	7131.83	115	
880	CKO	Male	5742.825	130	
825	CKO	Male	6858.37	331	
826	CKO	Male	5884.545	203	
831	CKO	Male	4889.436	79	
817	CKO	Female	5602.803	99	
854	CKO	Female	5416.474	52	
867	CKO	Female	6858.698	96	
871	CKO	Female	7279.954	155	
736	CKO	Female	4738.489	46	X removed for being over p150 on behavioral start date
739	CKO	Male	5916.42	147	X removed for being over p150 on behavioral start date
846	CKO	Male	5630.535	156	
866	CKO	Male	8293.492521	251	

Group 3					
Animal ID #			Distance	Vertical counts	
670	CKO	Male	5289.89	92	
609	CKO	Male	8388.42	189	
653	CKO	Male	5790.66	186	
634	CKO	Male	5622.68	186	
635	CKO	Male	5398.09	196	
638	CKO	Male	3863.71	227	
608	CKO	Male	6493.36	131	
669	CKO	Female	3171.46	37	
673	CKO	Female	3716.79	75	
611	CKO	Female	5082.68	58	
600	CKO	Female	6400.05	53	X removed for being over p150 on behavioral start date
633	CKO	Female	9170.47	137	
657	CKO	Female	6052.55	32	
671	dFIC	Male	4962.45	148	
692	dFIC	Male	7841.67	160	
693	dFIC	Male	4762	87	
652	dFIC	Male	3224.63	90	
654	dFIC	Male	3335.29	196	
664	dFIC	Female	4829.21	25	
674	dFIC	Female	6387.25	64	
682	dFIC	Female	4542.67	79	
683	dFIC	Female	5407.92	23	
684	dFIC	Female	5835.96	19	
613	dFIC	Female	8699.6	67	
614	dFIC	Female	5417.43	61	
655	dFIC	Female	8045.92	158	
680	dFIC	Female	4305.57	134	
681	dFIC	Female	5992.31	77	

Group 4					
Animal ID #			Distance	Vertical Counts	
719	CKO	Male	5719.36	164	
696	CKO	Male	6215.89	246	
724	CKO	Male	4761.11	89	
734	CKO	Male	4364.22	138	
746	CKO	Male	3563.14	124	X removed for incorrect genotype
738	CKO	Female	5668.14	53	
740	CKO	Female	5779.2	64	
718	CKO	Female	6484.73	77	
761	CKO	Female	3412	17	
707	CKO	Female	6557.7	116	
709	CKO	Female	6760.82	194	
710	CKO	Female	6769.94	98	
687	CKO	Female	9080.62	135	
694	dFIC	Male	7504.22	234	
717	dFIC	Male	6770.39	182	
729	dFIC	Male	7499.36	240	
720	dFIC	Female	7098.72	96	
763	dFIC	Female	6988.7	99	
688	dFIC	Female	8316.3	100	
697	dFIC	Female	19722.46	124	
737	dFIC	Female	7849.38	81	
723	dFIC	Female	8058	97	
708	dFIC	Female	7260.73	78	

Y MAZE

Group 1

Animal ID #			Alterations	% Correct Alterations	
840	Male	dFlC	19	70.58823529	
738	Male	dFlC	25	56.52173913	X removed for being over p150 on behavioral start date
841	Male	dFlC	29	55.55555556	
735	Male	dFlC	38	50	
876	Male	dFlC	34	56.25	
875	Male	dFlC	31	68.96551724	
824	Male	dFlC	26	75	
845	Male	dFlC	44	54.76190476	
850	Female	dFlC	19	64.70588235	
843	Female	dFlC	34	59.375	
842	Female	dFlC	29	59.25925926	
881	Female	dFlC	31	44.82758621	
815	Male	CKO	37	62.85714286	
883	Male	CKO	33	61.29032258	
886	Male	CKO	21	57.89473684	
866	Male	CKO	30	57.14285714	
825	Male	CKO	27	60	
831	Male	CKO	27	64	
880	Male	CKO	25	73.91304348	
826	Male	CKO	31	55.17241379	
856	Male	CKO	23	61.9047619	
817	Female	CKO	25	60.86956522	
867	Female	CKO	21	78.94736842	
854	Female	CKO	23	57.14285714	
871	Female	CKO	25	47.82608696	

Group 2

Animal ID #			Alterations	% Correct Alterations
82	CKO	Female	25	44
132	CKO	Female	31	61.29
155	CKO	Female	40	42.5
162	CKO	Female	49	40.82
175	CKO	Female	58	51.72
129	dFlC	Female	33	57.57
87	dFlC	Female	32	34.37
138	dFlC	Female	37	45.95
149	dFlC	Female	53	39.6
153	dFlC	Female	40	52.5
151	dFlC	Female	56	62.5
154	dFlC	Female	37	59.46
128	CKO	Male	39	43.15
131	CKO	Male	34	64.71
157	CKO	Male	47	44.68
135	CKO	Male	33	48.48
177	CKO	Male	43	48.84
178	CKO	Male	40	47.5
142	dFlC	Male	44	65.91
148	dFlC	Male	34	67.65
150	dFlC	Male	40	50
152	dFlC	Male	39	51.28
133	dFlC	Male	36	58.33
160	dFlC	Male	43	48.84
163	dFlC	Male	36	69.44

Group 3

Animal ID #			Alterations	% Correct Alterations	
670	CKO	Male	45	48.89	
609	CKO	Male	38	50	
653	CKO	Male	22	31.82	
634	CKO	Male	27	66.66	
635	CKO	Male	35	51.43	
638	CKO	Male	40	45	
608	CKO	Male	34	44.12	
669	CKO	Female	40	47.5	
673	CKO	Female	51	56.86	
611	CKO	Female	34	35.29	
600	CKO	Female	40	62.5	X removed for being over p150 on behavioral start date
633	CKO	Female	38	50	
657	CKO	Female	40	40	
671	dFlC	Male	40	57.5	
692	dFlC	Male	36	72.22	
693	dFlC	Male	28	60.71	
652	dFlC	Male	29	48.28	
654	dFlC	Male	32	50	
664	dFlC	Female	36	58.33	
674	dFlC	Female	48	60.42	
682	dFlC	Female	34	47.06	
683	dFlC	Female	40	65	
684	dFlC	Female	43	62.79	
613	dFlC	Female	38	42.11	
614	dFlC	Female	34	47.06	
655	dFlC	Female	38	57.89	
680	dFlC	Female	29	48.28	
681	dFlC	Female	29	34.48	

Group 4

Animal ID #			Alterations	% Correct Alterations	
719	CKO	Male	42	54.76	
696	CKO	Male	26	57.69	
724	CKO	Male	28	53.57	
734	CKO	Male	33	72.73	
746	CKO	Male	20	50	X removed for incorrect genotype
718	CKO	Female	39	58.97	
761	CKO	Female	40	70	
707	CKO	Female	43	53.49	
738	CKO	Female	44	56.82	
709	CKO	Female	38	55.26	
710	CKO	Female	52	53.85	
687	CKO	Female	57	50.88	
740	CKO	Female	42	52.38	
717	dFlox C	Male	29	48.28	
694	dFlox C	Male	33	42.42	
729	dFlox C	Male	21	55	
720	dFlox C	Female	36	50	
763	dFlox C	Female	35	37.14	
688	dFlox C	Female	60	60	
697	dFlox C	Female	67	50.75	
737	dFlox C	Female	48	58.33	
708	dFlox C	Female	29	51.72	
723	dFlox C	Female	51	45.1	

3 CHAMBER SOCIAL INTERACTION TESTS

Group 1			Socialbility			entries				Novelty			entries				
**Animal ID #**			**Unfamiliar**	**Center**	**Empty**	**Unfamiliar**	**Center**	**empty**	**Total**	**Familiar**	**Center**	**Unfamiliar**	**Familiar**	**Center**	**Unfamiliar**	**Total**	
735	Male	dFIC	285	129	186	12	24	13	49	316	82	202	15	25	11	51	
738	Male	dFIC	227	88	285	10	21	12	43	277	151	172	6	16	10	32	X removed for being over p150 on behavioral start date
840	Male	dFIC	200	157	243	11	22	11	44	253	149	198	11	21	11	43	
841	Male	dFIC	239	193	168	8	14	6	28	242	175	183	10	16	6	32	
845	Male	dFIC	187	225	188	10	18	7	35	160	152	288	11	27	15	53	
824	Male	dFIC	314	118	168	11	21	9	41	208	141	251	9	23	14	46	
875	Male	dFIC	287	131	182	12	19	7	38	218	167	215	11	24	13	48	
876	Male	dFIC	161	124	315	5	13	8	26	360	156	84	6	11	5	22	
881	Female	dFIC	275	100	225	7	15	8	30	179	146	275	7	16	9	32	
888	Female	dFIC	269	120	211	7	18	11	36	221	85	294	9	15	6	30	
850	Female	dFIC	204	113	283	7	15	8	30	281	94	225	12	21	9	42	
842	Female	dFIC	240	95	265	9	22	13	44	175	139	286	11	23	12	46	
843	Female	dFIC	170	129	301	9	17	8	34	239	149	212	10	23	13	46	
856	Male	CKO	262	111	227	9	20	10	39	286	170	144	10	18	8	36	
886	Male	CKO	225	112	263	14	28	14	56	373	135	92	23	32	9	64	
880	Male	CKO	385	121	94	10	17	6	33	198	160	242	12	22	10	44	
825	Male	CKO	249	119	232	6	12	6	24	353	106	141	8	13	5	26	
826	Male	CKO	169	118	313	5	13	8	26	204	132	264	9	14	5	28	
831	Male	CKO	253	104	243	7	14	7	28	254	79	267	8	14	6	28	
866	Male	CKO	263	66	271	8	17	9	34	219	142	239	14	25	11	50	
815	Male	CKO	155	130	315	6	17	11	34	303	65	232	7	16	9	32	
883	Male	CKO	304	94	202	7	14	7	28	287	86	227	11	20	9	40	
867	Female	CKO	242	106	252	8	15	7	30	314	156	130	9	17	8	34	
817	Female	CKO	271	115	214	9	18	8	35	219	113	268	7	17	10	34	
854	Female	CKO	225	111	264	9	19	10	38	331	97	172	10	20	10	40	
871	Female	CKO	271	115	214	9	19	11	39	268	144	188	12	26	14	52	

Group 2			Socialbility			entries				Novelty			entries				
**Animal ID #**			**Unfamiliar**	**Center**	**Empty**	**Unfamiliar**	**Center**	**Empty**	**Total**	**Familiar**	**Center**	**Unfamiliar**	**Familiar**	**Center**	**Unfamiliar**	**Total**	
87	Female	dFIC	139	174	287	4	12	7	23	247	78	275	5	12	7	24	
129	Female	dFIC	267	118	215	8	12	4	24	311	120	169	8	13	5	26	
138	Female	dFIC	124	175	301	5	15	10	30	195	168	237	5	13	8	26	
149	Female	dFIC	203	106	291	9	18	9	36	201	85	314	10	21	11	42	
153	Female	dFIC	260	112	228	7	16	9	31	195	181	224	6	16	10	32	
151	Female	dFIC	201	163	236	10	23	12	45	205	208	187	11	21	10	42	
82	Female	CKO	253	156	191	6	13	6	25	153	155	292	5	14	9	28	
132	Female	CKO	144	147	309	7	18	12	37	242	128	230	12	21	9	42	
155	Female	CKO	163	158	279	2	7	4	13	119	131	350	3	10	7	20	
162	Female	CKO	144	108	348	6	16	10	32	205	191	204	8	17	9	34	
240	Female	CKO	293	97	210	10	17	6	33	207	71	322	8	15	6	29	X
241	Female	CKO	270	182	148	3	8	5	16	303	81	216	5	10	5	20	
142	Male	dFIC	245	164	191	11	18	7	36	240	136	224	11	22	11	44	
133	Male	dFIC	335	96	169	6	11	5	22	181	112	307	12	22	10	44	
293 (renamed 160)	Male	dFIC	171	165	264	5	18	13	36	199	138	263	10	20	10	40	
163	Male	dFIC	216	130	254	4	14	10	28	219	159	222	6	16	10	32	
152	Male	dFIC	254	99	242	13	23	10	46	239	89	272	10	19	9	38	
150	Male	dFIC	266	99	235	5	14	10	29	228	87	285	8	16	8	32	
148	Male	dFIC	238	165	193	9	18	9	36	190	163	247	6	15	9	30	
178	Male	CKO	239	153	208	12	21	9	42	270	105	225	11	22	11	44	
177	Male	CKO	270	141	189	5	15	10	30	257	100	243	8	14	6	28	
296	Male	CKO	220	112	268	7	15	8	30	155	143	302	6	17	11	34	
292	Male	CKO	268	171	161	5	13	7	25	247	148	205	4	11	7	22	
291	Male	CKO	226	141	233	5	12	7	24	210	144	246	8	18	9	35	
157	Male	CKO	181	191	228	4	11	7	22	297	111	192	5	12	7	24	
128	Male	CKO	279	149	172	5	12	6	23	185	170	245	6	11	5	22	
131	Male	CKO	281	98	221	8	14	6	28	214	98	288	5	13	8	26	
135	Male	CKO	311	164	125	6	9	3	18	192	152	256	6	10	4	20	

Group 3			Socialbility				entries			Novelty			entries				
**Animal ID #**			**Unfamiliar**	**Center**	**Empty**	**Unfamiliar**	**Center**	**Empty**	**Total**	**Familiar**	**Center**	**Unfamiliar**	**Familiar**	**Center**	**Unfamiliar**	**Total**	
670	CKO	Male	123	250	227	8	16	7	31	147	197	256	4	11	6	21	
609	CKO	Male	270	87	243	9	18	9	36	only got 6 min camera died						0	X removed for equipment malfunction
653	CKO	Male	283	145	172	4	9	4	17	128	179	293	3	11	8	22	
634	CKO	Male	276	158	166	8	14	6	28	199	54	347	7	15	8	30	
635	CKO	Male	262	139	199	8	16	8	32	239	185	176	6	12	6	24	
638	CKO	Male	142	108	350	9	17	8	34	146	76	378	6	12	6	24	
608	CKO	Male	262	204	134	8	16	8	32	286	138	176	10	20	9	39	
669	CKO	Female	178	119	303	6	11	5	22	246	138	216	9	18	9	36	
673	CKO	Female	216	98	286	13	24	10	47	204	91	305	8	16	8	32	
611	CKO	Female	113	101	386	3	7	4	14	video didn't work camera issue						0	X removed for equipment malfunction
600	CKO	Female	212	151	327	6	12	6	24	245	95	260	8	15	7	30	X removed for being over p150 on behavioral start date
633	CKO	Female	229	166	205	11	21	9	41	344	161	95	11	18	6	35	
657	CKO	Female	306	121	173	7	10	3	20	283	116	201	2	8	5	15	
671	dFIC	Male	237	131	234	10	19	9	38	168	241	191	8	15	6	29	
692	dFIC	Male	246	107	247	7	16	8	31	179	34	387	3	7	4	14	
693	dFIC	Male	357	121	122	11	16	5	32	252	105	243	8	16	7	31	
652	dFIC	Male	299	186	115	6	12	6	24	204	119	277	8	18	9	35	
654	dFIC	Male	255	158	187	15	28	13	56	421	82	97	11	16	5	32	
664	dFIC	Female	133	341	126	3	8	4	15	148	173	279	7	14	7	28	
674	dFIC	Female	271	150	179	13	22	9	44	172	160	268	8	21	13	42	
682	dFIC	Female	228	171	201	5	13	8	26	227	128	245	9	19	10	38	
683	dFIC	Female	237	110	253	7	16	9	32	272	124	204	9	15	6	30	
684	dFIC	Female	only got 8 min total on this one						0	283	144	173	11	16	5	32	X removed for equipment malfunction
613	dFIC	Female	324	127	149	7	14	6	27	160	332	108	4	7	3	14	
614	dFIC	Female	189	190	221	4	9	5	18	165	165	270	6	16	9	31	
655	dFIC	Female	184	174	242	8	20	12	40	209	178	213	9	18	9	36	
680	dFIC	Female	239	167	194	7	12	5	24	295	137	168	9	20	11	40	
681	dFIC	Female	210	186	204	6	13	6	25	206	151	243	7	16	8	31	

Group 4			Socialbility				entries			Novelty			entries				
**Animal ID #**			**Unfamiliar**	**Center**	**Empty**	**Unfamiliar**	**Center**	**Empty**	**Total**	**Familiar**	**Center**	**Unfamiliar**	**Familiar**	**Center**	**Unfamiliar**	**Total**	
719	CKO	Male	239	102	259	9	20	11	40	203	85	312	8	18	10	36	
696	CKO	Male	337	104	159	11	16	5	32	377	62	161	6	10	5	21	
724	CKO	Male	392	79	129	7	11	4	22	*no video						0	X removed for equipment malfunction
734	CKO	Male	322	128	150	8	13	5	26	245	173	182	8	14	7	29	
746	CKO	Male	236	74	290	6	18	12	36	268	29	303	6	13	8	27	X removed for incorrect genotype
718	CKO	Female	178	118	304	6	20	14	40	*no video						0	X removed for equipment malfunction
761	CKO	Female	*video cut short						0	382	54	164	5	9	4	18	X removed for equipment malfunction
707	CKO	Female	271	110	219	8	14	6	28	214	129	257	8	15	8	31	
738	CKO	Female	284	108	208	11	20	9	40	142	88	370	6	14	8	28	
709	CKO	Female	270	104	226	7	15	8	30	443	45	112	5	7	3	15	
710	CKO	Female	237	122	241	8	16	8	32	297	95	208	9	18	9	36	
687	CKO	Female	225	188	187	7	18	10	35	251	138	211	7	12	5	24	
740	CKO	Female	258	141	201	13	20	7	40	277	79	244	8	14	6	28	
717	dFIC	Male	270	113	217	12	21	10	43	235	118	247	9	18	9	36	
694	dFIC	Male	270	150	180	9	17	8	34	234	92	274	8	18	10	36	
729	dFIC	Male	243	117	240	9	18	8	35	323	95	182	6	14	8	28	
720	dFIC	Female	293	97	210	13	22	9	44	331	92	177	8	16	9	33	
763	dFIC	Female	251	118	231	11	19	8	38	172	44	384	6	13	7	26	
688	dFIC	Female	234	156	210	13	23	11	47	247	119	234	14	26	12	52	
697	dFIC	Female	194	153	253	10	22	12	44	227	201	172	9	17	8	34	
737	dFIC	Female	209	146	245	12	26	14	52	222	129	249	8	15	7	30	
708	dFIC	Female	203	129	268	7	13	7	27	210	101	289	4	9	5	18	
723	dFIC	Female	214	139	247	12	13	11	36	360	82	158	10	15	6	31	

Supplemental Group			Socialbility				entries			Novelty			entries			
**Animal ID #**			**Unfamiliar**	**Center**	**Empty**	**Unfamiliar**	**Center**	**Empty**	**Total**	**Familiar**	**Center**	**Unfamiliar**	**Familiar**	**Center**	**Unfamiliar**	**Total**
24699	B6	Male	317	121	162	13	23	9	45	185	138	277	12	24	12	48
24604	B6	Male	265	123	212	12	20	8	40	211	86	303	12	22	10	44
24605	B6	Male	219	145	236	4	12	8	24	244	102	252	8	19	11	38
24581	B6	Female	205	167	228	15	30	15	60	118	182	300	8	21	13	42
24580	B6	Female	220	169	211	10	17	7	34	343	132	125	7	11	4	22
24579	B6	Female	248	152	200	9	17	8	34	135	249	216	8	16	8	32
B1	B6	Female	258	152	190	16	25	8	49	167	157	276	12	25	12	49
B2	B6	Female	287	197	116	14	21	7	42	164	194	242	13	26	13	52
B3	B6	Female	271	153	176	16	27	11	54	160	144	296	11	24	13	48
B4	B6	Female	328	148	124	9	15	6	30	232	182	186	12	23	12	47
157	dFIC	Male	240	111	249	7	15	8	30	146	135	319	10	19	9	38
155	dFIC	Male	241	143	216	7	18	10	35	161	141	298	8	19	10	37
156	dFIC	Male	218	155	227	10	21	11	42	166	132	302	14	27	13	54
146	dFIC	Male	231	184	185	7	16	9	32	141	154	305	9	16	7	32
141	dFIC	Male	173	166	261	7	17	10	34	180	127	293	9	20	11	40
143	dFIC	Female	194	176	230	9	17	8	34	199	184	217	11	22	10	43
152	dFIC	Female	196	160	244	21	38	16	75	211	122	267	16	37	20	73
153	dFIC	Female	238	123	239	12	24	11	47	164	179	257	11	31	19	61
154	dFIC	Female	203	170	227	12	27	15	54	217	169	214	14	31	17	62
176	dFIC	Female	284	137	179	8	13	5	26	209	141	250	7	20	13	40
174	dFIC	Female	234	188	178	13	28	15	56	238	111	251	13	23	10	46
175	dFIC	Female	199	219	182	9	21	11	41	200	142	258	8	17	10	35

PRE-PULSE INHIBITION

	dFIC									
**67db**	25.87044	0.339681	16.67506	34.96037	42.95708	19.14538	50.1404	33.90143	36.81972	12.54528
**70dB**	35.37908	41.00618	5.698992	7.092199	38.15492	51.31717	12.98758	34.61614	52.86907	21.093
**73dB**	38.32787	52.35461	12.85894	29.53692	6.915398	47.54499	40.72025	30.31612	43.82067	30.21365
**76dB**	29.3396	52.93361	27.05919	40.0292	5.909786	31.04941	80.27673	38.5431	64.53292	34.67694

	CKO					
**67db**	41.5694	53.39474	74.92798	54.53537	27.68058	23.82918
**70dB**	11.61054	54.18197	63.79641	38.06831	58.02593	34.8094
**73dB**	19.13759	53.00734	72.52721	19.04155	47.83762	50.04299
**76dB**	58.52506	83.81978	56.98624	9.57899	57.38477	51.27544

FEAR CONDITIONING

Group 1						
Animal ID #	% Freezing			Post-FS	Test	
840	Male	dFIC	0	16.3	21.4	
738	Male	dFIC	0	5.6	13.5	X removed for being over p150 on behavioral start date
735	Male	dFIC	0	37.4	21.6	
845	Male	dFIC	0	11.2	5.9	
875	Male	dFIC	0	16	31.3	
824	Male	dFIC	0.7	7.5	14.7	
876	Male	dFIC	0	32.7	26.7	
841	Male	dFIC	92.3	98.3	34.6	X removed for being an outlyer
842	Female	dFIC	0	0.3	0.8	
850	Female	dFIC	0	28.5	14.8	
843	Female	dFIC	0	8.9	4.9	
881(renamed 815)	Female	dFIC	0	34.9	38.4	
888	Female	dFIC	0	12.2	1.5	
886	Male	CKO	0	2	0.6	
883	Male	CKO	0	24.3	12.9	
891	Male	CKO	0	19.5	22.9	
880	Male	CKO	0	12.6	22.4	
825	Male	CKO	0	15.6	52.2	
856	Male	CKO	0	15.4	19.2	
866	Male	CKO	0	2.1	5.5	
831	Male	CKO	0	4.8	1.2	
826	Male	CKO	0	8.9	15.5	
817	Female	CKO	0	46.3	36.3	
867	Female	CKO	0	10.2	0	
854	Female	CKO	0	35.8	38.5	
871	Female	CKO	0	4.4	1.2	

Group 3						
Animal ID #	% Freezing			Post-FS	Test	
670	CKO	Male	0	27.6	26.3	
609	CKO	Male	0	3.5	2.1	
635	CKO	Male	0	36.2	57.6	
638	CKO	Male	0	8.4	3.5	
608	CKO	Male	0	0.3	0	
653	CKO	Male	0	21.5	5.5	
634	CKO	Male	0	1	0.6	X removed for equipment malfunction
669	CKO	Female	0	22	5.8	
673	CKO	Female	0	10.4	13	
611	CKO	Female	0	12.4	32.1	X removed for being over p150 on behavioral start date
600	CKO	Female	0	7.6	0	
633	CKO	Female	0	19.8	0	
657	CKO	Female	0	31.1	1.4	
671	dFIC	Male	0	2	1.2	
692	dFIC	Male	0	0.5	3.3	
693	dFIC	Male	0	17.7	10.1	
652	dFIC	Male	0	23.2	36.6	
654	dFIC	Male	0	5.8	3.3	
664	dFIC	Female	0	36	3.3	
674	dFIC	Female	0	22	2.9	
682	dFIC	Female	0	13.7	1.1	
683	dFIC	Female	10.1	62.5	51.5	
684	dFIC	Female	0	12.6	18.3	
613	dFIC	Female	0	39.2	74.4	
614	dFIC	Female	0	44.7	62.7	
655	dFIC	Female	0	10.9	0	
680	dFIC	Female	0	15.6	8.2	
681	dFIC	Female	0	32.3	14.9	

Group 4						
Animal ID #	% Freezing			Post-FS	Test	
719	CKO	Male	0	2.4	0.7	
696	CKO	Male	0	0.6	0	
724	CKO	Male	0	2.3	21.4	
734	CKO	Male	0	9.2	12.4	
746	CKO	Male	0	18.2	40.7	X removed for incorrect genotype
738	CKO	Female	0	29.2	0.6	
740	CKO	Female	0	4	11	
718	CKO	Female	0	11.6	23	
761	CKO	Female	0	13.1	46.6	
707	CKO	Female	0	18	0	
709	CKO	Female	0	36.6	12.1	
710	CKO	Female	0	9.2	3.3	
687	CKO	Female	0	0	3.3	
694	dFIC	Male	0	1	0	
717	dFIC	Male	0	0.3	0	
729	dFIC	Male	0	0.9	1.5	
720	dFIC	Female	0	0	0.7	
763	dFIC	Female	0	48.7	0	
688	dFIC	Female	0	1.5	0	
697	dFIC	Female	0	0.9	2.7	
737	dFIC	Female	0	0.9	0	
723	dFIC	Female	0	38.5	54.9	
708	dFIC	Female	0	24.8	3.9	

AAV2/9 CaMKIIa-YFP-difopein

	Baseline	After Injection
**Animal 1**	5427.52	13660.53
**Animal 2**	6653.38	17318.71
**Animal 3**	3465.37	12919.99
**Animal 4**	6526.93	17971.68
**Animal 5**	8421.3	14512.99
**Animal 6**	5773.44	17539.21

AAV9-CAG-DIO-YFP-difopein

	Baseline	After Injection
**Animal 1**	6522.01	5022.46
**Animal 2**	3122.97	7544.64
**Animal 3**	4010.18	6837.08
**Animal 4**	4442.12	6918.21
**Animal 5**	3431.99	3994.57
**Animal 6**	2932.56	5934.42
